# Harnessing local and system immune profiling delineating differential responders to first-line sintilimab (anti-PD-1 antibody) combined with chemotherapy in extensive-stage small cell lung cancer: an exploratory biomarker analysis of a phase II study

**DOI:** 10.1038/s41392-025-02252-5

**Published:** 2025-05-23

**Authors:** Mengqing Xie, Minwei Bao, Xiaorong Dong, Lin Wu, Li Liu, Jing Zhao, Xiangling Chu, Yan Wu, Xianxiu Ji, Yujia Fang, Xin Yu, Shiji Zhang, Qi Wang, Tao Hu, Jin Wang, Changbin Zhu, Chunxia Su

**Affiliations:** 1 Department of Medical Oncology, Shanghai Pulmonary Hospital, Tongji University School of Medicine, Tongji University, Shanghai, China; 2 Department of Thoracic Surgery, Shanghai Pulmonary Hospital, Tongji University School of Medicine, Tongji University, Shanghai, China; 3 Cancer Center, Union Hospital, Tongji Medical College, Huazhong University of Science and Technology, Wuhan, China; 4 Hunan Cancer Hospital, The Affiliated Cancer Hospital of Xiangya School of Medicine, Changsha, China; 5 Amoy Diagnostics Co., Ltd., Xiamen, Fujian China

**Keywords:** Cancer microenvironment, Prognostic markers

## Abstract

While chemo-immunotherapy has been established as the frontline therapeutic regimen for extensive-stage small cell lung cancer (ES-SCLC), durable responses persist predominantly in a minor population of patients. This underscores critical need to elucidate underlying local tumor microenvironment and systematic immune profiles for biomarker discovery. In this phase II trial (ChiCTR2000038354), the efficacy, safety, and immune-genomic signatures of sintilimab (anti-PD-1 antibody) synergized with chemotherapy as first-line regimen for ES-SCLC were evaluated. The regimen demonstrated a median progression-free survival (PFS) of 6.9 months and median overall survival of 17.1 months, accompanied by a 12-month PFS rate of 16.9%, fulfilling the primary endpoint. Manageable grade 3 or 4 treatment-related adverse events developed in 27.3% (12/44) of patients. The exploratory study indicated a higher infiltration of CD4^+^/CD8^+^ CXCR5^+^ T follicle helper cells, CD8^+^CD103^+^ tissue-resident memory T cells, and B cells in tumor tissue, associated with better response and prognosis. The study also indicated the presence of tumor macrophages (CD68^+^CD163^+^CSF1R^+^SIGLEC5^+^) associated with immunotherapy resistance. Higher levels of monocyte-dendritic cells in pre-treatment peripheral blood mononuclear cells were found in durable clinical benefit group. Also, higher CD83, CD244, IL-12, and CD70, which are hallmarks of dendritic cells and activated T cells, were discovered by plasma proteomics to be connected with enhanced outcomes, while chemoattractant of macrophage, CSF-1, CCL3, CCL4, and IL-8, were found to predict a worse prognosis. Furthermore, a multimodal model was constructed and validated for stratifying ES-SCLC into high or low risk to predict the immunotherapy efficacy. This study sheds light on harnessing local and systematic immune profiles to better stratify patients with ES-SCLC for immunotherapy and putative combinational treatment.

## Introduction

Small cell lung cancer (SCLC) is recognized as a rapidly progressing malignancy, constituting 15% of newly diagnosed lung cancer cases, with the majority patients identified at the extensive stage (ES-SCLC) according to Veterans Administration Lung Cancer Study Group.^1,2^ ES-SCLC patients, with dismal prognosis, had constrained therapeutic landscape. Etoposide and platinum remained as first-line standard of strategy over several decades. Recent advancements in immune checkpoint inhibitors (ICIs) have demonstrated survival benefits in SCLC management. Particularly, the integration of programmed death-ligand 1 (PD-L1) blockade with chemotherapy has presented with significant potential in improving survival rates. IMpower133 was the first phase III clinical trial exploring the efficacy of ICIs plus chemotherapy in ES-SCLC, the addition of atezolizumab significantly prolonged survival duration (progression-free survival (PFS): median 5.2 vs. 4.3 months; hazard ratio (HR) = 0.77, 95% confidence intervals (CI): 0.62–0.96; overall survival (OS): median 12.3 vs. 10.3 months; HR = 0.70, 95% CI: 0.54–0.91) compared to chemotherapy alone.^3^ The CASPIAN and CAPSTONE-1 trials, utilizing durvalumab and adebrelimab respectively, have validated the significant clinical benefits of ICIs, providing pivotal evidence for therapeutic strategies in ES-SCLC as well.^4,5^ These landmark trials prompted guideline updates incorporating PD-L1 blockade into standard care strategies of ES-SCLC.

In addition to PD-L1 blockade, several recently reported randomized phase III studies, such as ASTRUM-005, RATIONALE-312, and EXTENTORCH, focusing on anti-programmed death-1(PD-1) antibody, yielded satisfactory results. The ASTRUM-005 trial demonstrated serplulimab’s superior efficacy over standard chemotherapy, with significant improvements in both PFS (median 5.7 vs 4.3 months; HR = 0.48, 95% CI 0.38–0.59) and OS (median 15.4 vs 10.9 months; HR = 0.63, 95% CI 0.49–0.82).^6^ Similarly, the RATIONALE-312 trial revealed tislelizumab’s clinical benefit, showing enhanced PFS (median 4.7 vs 4.3 months; HR = 0.64, 95% CI 0.52–0.78) and OS outcomes (median 15.5 vs 13.5 months; HR = 0.75, 95% CI 0.61–0.93).^7^ Toripalimab presented with promising results, showing prolonged PFS (median 5.8 vs 5.6 months; HR = 0.67, 95% CI 0.54–0.82) and OS (median 14.6 vs 13.3 months; HR = 0.80, 95% CI 0.65–0.98) as well.^8^ These landmark studies proposed anti-PD-1 antibodies as viable therapeutic options in the ES-SCLC treatment paradigm. Though, the advent of ICIs has redefined therapeutic paradigms in SCLC, persistent deficiencies in predictive biomarker for patient stratification continue to hinder precision oncology implementation. This underscores the critical imperative to elucidate the molecular underpinnings of SCLC and identify reliable biomarkers for immunotherapy response.

The quest for reliable biomarkers to predict immunotherapy response in SCLC has evolved through iterative clinical and molecular investigations. Initial efforts focused on extrapolating established biomarkers from non-small cell lung cancer (NSCLC), such as PD-L1 expression and tumor mutational burden (TMB).^9^ Paradoxically, in SCLC, the value of these markers for immunotherapy responsiveness remains controversial. In studies analysing IMpower133^10^ (≥5% in tumor cells or immune cells) and CASPIAN^11^ (1% in tumor cells or immune cells), it was found that although SCLC with high PD-L1 expression showed a better survival in the immune checkpoint blockade group, no statistically significant differences were identified. Similarly, TMB was analyzed in the EXTENTORCH study,^8^ where the results indicated that regardless of whether TMB was high (defined as ≥10 mut/Mb) or low, the PFS and OS benefits of the toripalimab combined with chemotherapy were similar, with no statistically significant differences detected. Emerging transcriptome-based molecular classification of SCLC provides critical insights into patient stratification for immune checkpoint blockade therapeutic strategies. Current frameworks delineate four molecular subtypes (SCLC-A, SCLC-N, SCLC-P, and SCLC-I) defined by transcriptional regulators (ASCL1, NEUROD1, POU2F3, and inflamed gene signatures, respectively). SCLC-I is thought to respond favourably to immunotherapy.^12^ In IMpower133 trial, only 18% of patients stratified as SCLC-I subtype, characterized by inflamed tumor microenvironments, achieved durable clinical benefit (≥12-month progression-free survival) from first-line atezolizumab-carboplatin-etoposide therapy.^12^ While growing evidence suggests the existence of biomarkers in SCLC, universally applicable and clinically relevant biomarkers for predicting immunotherapy efficacy have yet to be identified.

A critical barrier to identify biomarkers for ICIs efficacy in SCLC stems from the disease’s inherent biological complexity and the scarcity of high-quality specimens. To address the obstacle, we administered a phase II investigator-initiated trial (IIT) (ChiCTR2000038354), which evaluated the efficacy, safety, and biomarkers of sintilimab (anti-PD-1 antibody) synergizing with chemotherapy as the first-line regimen for ES-SCLC. This trial incorporated a comprehensive biomarker discovery framework, uniquely integrating multi-omics analyses of paired pretreatment tumor tissues and blood samples. By using whole-exome sequencing (WES), RNA sequencing, multiplexed immunofluorescence (mIF), flow cytometry (FCM), and plasma proteomics assays on pre-treatment tumor samples and blood samples from SCLC, we delineated distinct immunological signatures within the tumor microenvironment that demonstrated significant correlation with clinical responsiveness to ICIs. Furthermore, clinical variables and plasma proteomic parameters were integrated to develop a non-invasive predictive model for immunotherapy response. This model was subsequently validated in an independent external cohort. Our study proposes the novel conceptual framework and translational prospects for understanding the local and systemic immune profiles for differential response to immunotherapy. Based on these findings, a clinically accessible, non-invasive model was proposed to putatively guide risk stratification and immunotherapy strategies for ES-SCLC patients.

## Results

### Study overview

**Fig. 1:**
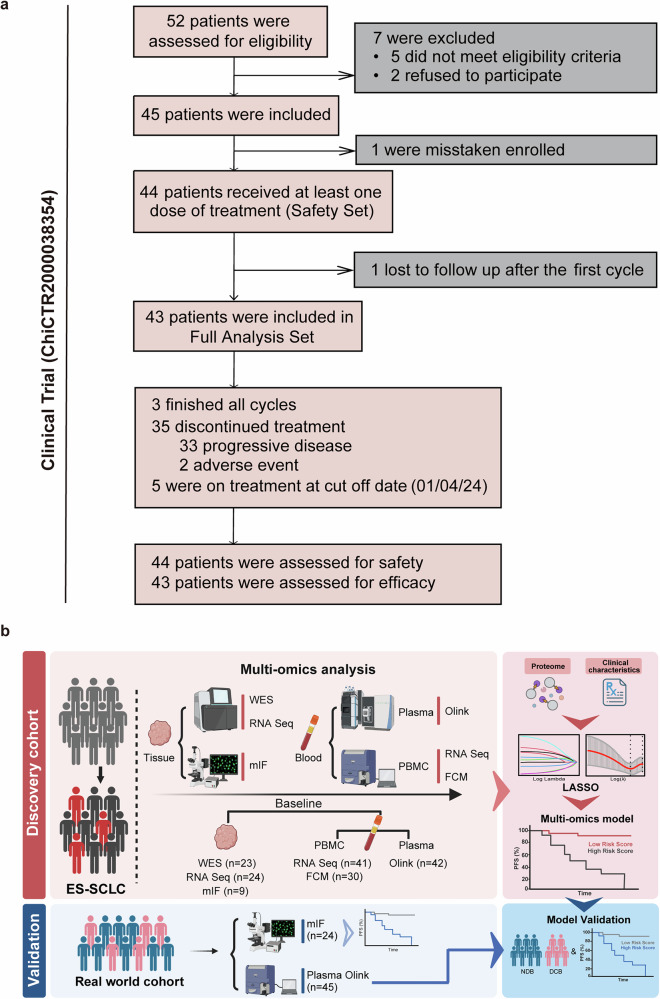
Study design. **a**. Of a total of 52 patients screened for study eligibility, five did not meet the inclusion criteria, two declined to participate and one was mistakenly enrolled. A total of 44 enrolled patients accepted at least one cycle of sintilimab plus chemotherapy and were assessed for safety, while 43 patients were evaluable for radiographic response assessment and efficacy. **b**. Design of the sample collection for biomarker research. Tumor samples and blood were collected at baseline. Validation of findings on the real-world cohort 1 (*n* = 24) and real-world cohort 2 (*n* = 45)

Between December 10, 2021, and September 13, 2023, 52 patients were screened. Of these, 44 were included in the safety analysis (Safety Set). One discontinued follow-up after one cycle and did not undergo an efficacy assessment. Consequently, 43 were eligible for efficacy analysis (Full Analysis Set, FAS) (Fig. 1a).

Clinical-demographic profiles of the FAS cohort are systematically cataloged in Table 1. The cohort demonstrated a median age of 65 years (interquartile range (IQR), 60–69 years). Forty of the patients (93.0%) were male. All exhibited an Eastern Cooperative Oncology Group performance status (ECOG-PS) of 1. All cases received carboplatin. Most (41, 95.3%) were current or former smokers. Thirty-seven (86.0%) had stage IV disease at diagnosis. Seventeen (39.5%) had increased lactate dehydrogenase concentration (LDH). Two (4.7%) patients were with brain metastases, nine (20.9%) with liver metastases and thirteen (30.2%) with bone metastases at baseline. Thirty-one (72.1%) had a PD-L1 expression (22C3) less than 1%.

**Table 1:** Baseline Characteristics of Patients in FAS (*n* = 43)

Characteristic	No. of patient (%)
**Age, years**	
Median age (IQR)	65 (60–69)
Age group	
<65 years	21 (48.8)
≥65 years	22 (51.2)
Sex	
Male	40 (93.0)
Female	3 (7.0)
ECOG performance status	
0	0 (0.0)
1	43 (100.0)
Smoking status	
Never	2 (4.7)
Current or Former	41 (95.3)
AJCC stage^8th^	
IIIc	6 (14.0)
IVa	21 (48.8)
IVb	16 (37.2)
LDH at baseline	
≤ULN	26 (60.5)
>ULN	17 (39.5)
Brain metastasis	
Yes	2 (4.7)
No	41 (95.3)
Liver metastasis	
Yes	9 (20.9)
No	34 (79.1)
Bone metastasis	
Yes	13 (30.2)
No	30 (69.8)
PD-L1	
<1%	31 (72.1)
≥1%	7 (16.3)
Unknown	5 (11.6)

Data cutoff: April 1, 2024

Data are number of patients (% of patients)

*FAS* full analysis set, *IQR* interquartile range, *ECOG* Eastern Cooperative Oncology Group, *AJCC* American Joint Committee on Cancer, *LDH* lactate dehydrogenase, *ULN* upper limit of normal

WES (*n* = 23), RNA sequencing (*n* = 24), and mIF (*n* = 9) were conducted on available baseline tumor tissues. RNA sequencing (*n* = 41) and FCM (*n* = 30) were performed on baseline peripheral blood mononuclear cells (PBMC), and plasma proteomics assays (*n* = 42) on baseline plasma. A multi-omics analysis was conducted. The validation cohorts were all ES-SCLCs who received approved first-line immunotherapy in China. They included: 1) 24 baseline tumor tissues for mIF and 2) 45 baseline plasma for proteomics validation (Fig. 1b). The clinical pathological characteristics of our IIT cohort alongside the mIF validation cohort (real-world cohort 1) and the proteomics validation cohort (real-world cohort 2) were generally comparable (Supplementary Tables 1–2).

### Efficacy and safety

**Fig. 2:**
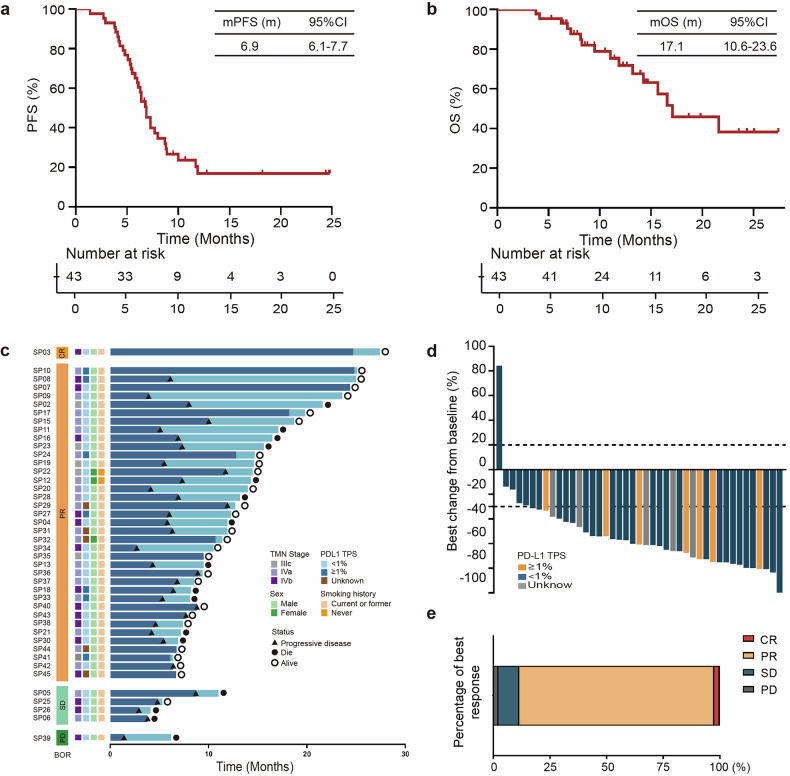
Efficacy of study. **a** Kaplan–Meier curve of PFS for participants (*n* = 43). **b** Kaplan-Meier curve of OS for participants (*n* = 43). **c** Individual clinicopathological features and treatment response. Each participant was represented as a line, with OS (blue) and PFS (dark blue) plotted for 43 individuals. Events were marked with a black triangle for PD and a black dot for death. **d** Waterfall plot of the best response in ES-SCLC. Best change of target lesion size compared with baseline (*n* = 43). The horizontal lines at −30 and 20 represent the thresholds defining an objective response according to RECIST v1.1. **e** Best response of enrolled patients (*n* = 43)

At the data cut-off date (April 1, 2024), the median follow-up time was 14.5 months (IQR, 7.5–15.7 months). Three patients completed all cycles, and five patients were on treatment. Among 35 patients who discontinued, 33 experienced either disease progression or death. According to the investigator, the median PFS was 6.9 months (95% CI, 6.1–7.7 months, Fig. 2a). The PFS rate at 12 months was 16.9%, which met the trial’s primary endpoint. The median OS was 17.1 months (95% CI, 10.6–23.6 months), and the OS rate at 12 months was 71.6% (Fig. 2b).

The overall investigator objective response rate (ORR) was 88.4%, and disease control rate was 97.7% (Fig. 2c–e). Patients were categorized into durable clinical benefit (DCB) or non-durable benefit (NDB) group, depending on whether the PFS of ES-SCLC was greater than 6 months^13^ (Supplementary Fig. 1a–b).

Subgroup analysis showed differences in PFS (HR = 2.3, 95% CI 1.14–4.65, *P* = 0.020) and OS (HR = 3.68, 95% CI 1.35–10.03, *P* = 0.011) between patients with LDH above normal and those with normal LDH. Patients with liver metastasis had significantly reduced PFS (HR = 2.92, 95% CI 1.26–6.74, P = 0.012) and OS (HR = 4.7, 95% CI 1.26–17.59, *P* = 0.021) compared to those without liver metastasis. Patients with bone metastasis also had poorer OS (HR = 3.37, 95% CI 1.23–9.2, *P* = 0.018) (Supplementary Fig. 1c).

The median number of administered treatment cycles was 9 (range, 1–34), 44 patients were included in the safety analysis. At least one treatment-related adverse event (TRAE) was recorded in 43 patients (43/44, 97.7%). Grade 3 or 4 TRAEs occurred in 12 out of 44 patients (27.3%). The most common TRAEs were anemia (93.2%), fatigue (56.8%), decreased appetite (52.3%), constipation (52.3%), nausea (47.7%), and decreased neutrophil count (45.5%). The most prevalent grade 3 or 4 TRAEs were hematologic: 7 patients (15.9%) showed decreased neutrophil count, followed by 6 (13.6%) with decreased white cell count and 3 (6.8%) with decreased platelet count. Other grade 3 or 4 TRAEs included anemia, proteinuria, increased creatinine, hyponatremia, hypophysis, acute kidney injury, and pain in the extremities, all occurring at a frequency of 2.3%. TRAEs led to treatment discontinuation in two cases, and 26 (59.1%) patients had their treatment delayed (Table 2).

**Table 2:** Treatment-related adverse events (*n* = 44)

Adverse events	Any grade *n* (%)	Grade 3–4 *n* (%)
Any grade	43 (97.7)	12 (27.3)
Anemia	41 (93.2)	1 (2.3)
Fatigue	25 (56.8)	0 (0.0)
Decreased appetite	23 (52.3)	0 (0.0)
Constipation	23 (52.3)	0 (0.0)
Nausea	21 (47.7)	0 (0.0)
Neutrophil count decreased	20 (45.5)	7 (15.9)
Diarrhea	20 (45.5)	0 (0.0)
ALT increased	18 (40.9)	0 (0.0)
AST increased	18 (40.9)	0 (0.0)
Platelet count decreased	17 (38.6)	3 (6.8)
Proteinuria	17 (38.6)	1 (2.3)
Bilirubin increased	14 (31.8)	0 (0.0)
Creatinine increased	13 (29.5)	1 (2.3)
White cell count decreased	12 (27.3)	6 (13.6)
Vomiting	12 (27.3)	0 (0.0)
GGT increased	10 (22.7)	0 (0.0)
Hyperglycemia	9 (20.5)	0 (0.0)
LDH increased	9 (20.5)	0 (0.0)
Hematuria	8 (18.2)	0 (0.0)
ALP increased	8 (18.2)	0 (0.0)
Hypokalemia	8 (18.2)	0 (0.0)

Data cutoff: April 1, 2024

Data are number of patients (% of patients)

TRAEs with an incidence of ≥10% of patients are reported

AEs were graded based on CTCAE version 5.0

*ALT* alanine aminotransferase, *AST* aspartate aminotransferase, *GGT* gamma-glutamyl transpeptidase, *LDH* lactate dehydrogenase, *ALP* alkaline phosphatase

### Genomic landscape of ES-SCLC and clinical outcomes

To elucidate the mutational landscape of ES-SCLC at baseline, we performed WES on the available 23 baseline samples. Consistent with the known genomic variations, *TP53* and *RB1* emerged as the predominant genes harboring mutations, with mutation rates of 65.2% and 34.8% respectively. Additionally, mutations ( >15% frequency) in genes involved in *KMT2D*, *ZFHX3*, *EP300*, *AHNAK2*, and *NOTCH1* had been identified, suggesting potential alternative drivers in SCLC pathogenesis (Supplementary Fig.2a).

Given the established role of *RB1* in SCLC biology, we conducted survival analysis based on the mutation status of *RB1*. ES-SCLC with wild type *RB1* showed superior PFS but discrepancy in OS was not significant (PFS: HR = 0.35, 95%CI: 0.13–0.96; OS: HR = 0.48, 95%CI: 0.16–1.47; Supplementary Fig. 2b-c). The association between tissue TMB and survival was also not significant (PFS: HR = 1.22, 95%CI: 0.5–2.95; OS: HR = 1.64, 95%CI: 0.56–4.8) (Supplementary Fig. 2d–e). Furthermore, no statistically significant difference between *TP53/RB1* co-mutations and ICI efficacy was established (Supplementary Fig. 2f-i).

SCLC molecular subtypes were assessed based on previous studies.^12,14,15^ No significant correlation to immune benefit was observed (Supplementary Fig. 3a-c).

### Tumor-associated macrophages and T/B lymphocyte subpopulations delineated the efficacy of immunotherapy in ES-SCLC

To investigate tumor microenvironment (TME) and its impact on immunotherapy efficacy, we conducted RNA sequencing on 24 baseline biopsy samples in our IIT cohort.

CIBERSORT analysis confirmed that CD8^+^ T cells and M2 macrophages were abundant in DCB and NDB groups, respectively (Supplementary Fig. 4a-b), and correlated with PFS (Supplementary Fig. 4c–e). The patients were further stratified based on T cell and M2 macrophage status (Supplementary Fig. 5a–b). Patients with T-cell infiltration showed better survival outcomes, while those with M2 profiles had significantly worse PFS and OS (Supplementary Fig. 5c–e). The differential infiltration of T cells and M2 macrophages were validated in baseline biopsies (*n* = 9) (Supplementary Fig. 5f–g).

**Fig. 3:**
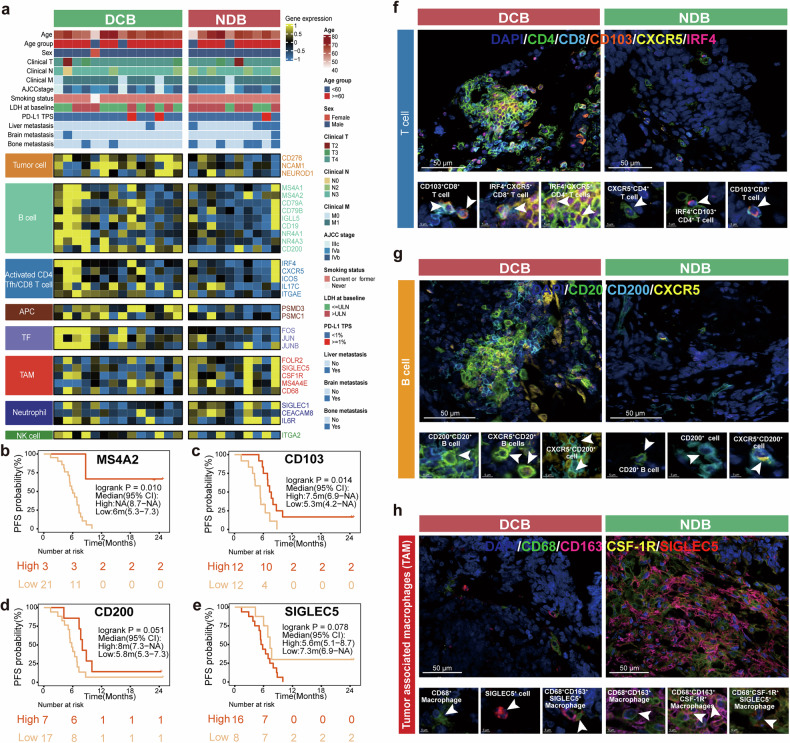
Correlation between baseline tumor TME and prognosis in ES-SCLC**. a** Clinicopathological characteristics and corresponding transcriptomic features of DCB and NDB groups, displayed by different cell types. **b**–**e** Kaplan–Meier curves for PFS. **f–h** Representative images of multiplex immunofluorescence staining in DCB and NDB groups for T cells, B cells and TAMs. Scale bars: 50 μm (upper); 5 μm (lower)

The subtypes of T cells and macrophages were further analyzed through RNA sequencing. The heatmap was organized according to immune cell populations and visualized, which revealed key genes characterizing specific immune cell populations in SCLC TME. B cell markers (*MS4A1, CD19, IGLL5*, and *CD200*), together with markers of follicular T cells (*IRF4* and *CXCR5*) and tissue-resident memory T cells (*ITGAE*, also named *CD103*), were highly expressed in the DCB group, while markers of immune suppressive tumor-associated macrophages (TAM) (*CSF1R, SIGLEC5* and *CD68*) were enriched in the NDB group (Fig. 3a). Furthermore, the expression of key genes and the evaluation of PFS and OS revealed that a higher expression of *MS4A2*, *CD103*, and *CD200* corresponded to better prognosis (*MS4A2* PFS: not reached vs 6 m, *P* = 0.01; *CD103* PFS: 7.5 m vs 5.3 m, *P* = 0.014; *CD200* PFS: 8 m vs 5.8 m, *P* = 0.051, Fig. 3b–d), whereas a higher *SIGLEC5* expression was linked to worse prognosis (*SIGLEC5* PFS: 5.6 m vs 7.3 m, *P* = 0.078; Fig. 3e). Moreover, these genes displayed similar trends in OS (Supplementary Fig. 6a-d).

In parallel, CD4^+^/CD8^+^ CXCR5^+^T follicular helper cells and CD8^+^CD103^+^ tissue-resident memory T cells were observed infiltrating in tumor tissues (Fig. 3f), Moreover, CD200 expressing B (CD20^+^CD200^+^) and T cells (CXCR5^+^CD200^+^) accumulated in the DCB groups (Fig. 3g). Conversely, the NCD group exhibited an enhanced infiltration of TAM with distinct immune phenotype, characterized by the co-commitment expression of CD68, CD163, CSF1R, and SIGLEC5, which was highly concordant with transcriptomic data (Fig. 3h).

Additionally, mIF had been used to validate the results in the real-world cohort of ES-SCLC (*n* = 24) who had first-line atezolizumab plus chemotherapy. The clinicopathological information showed comparability between the validation cohort and the study cohort (Supplementary Table 1). T cell with the expression of CD8/CD103/CXCR5 had been linked to favorable outcomes (HR < 1 and *P* < 0.05), while TAM with expression CD68/CD163/SIGLEC5/CSF1R had been associated with adverse outcomes (HR > 1 and *P* < 0.05) (Supplementary Fig. 7a–c). Moreover, a significant association was identified between the density of these markers and PFS outcomes (Supplementary Fig. 7d–l).

### Prognostic insights from pre-treatment PBMC profiles

**Fig. 4:**
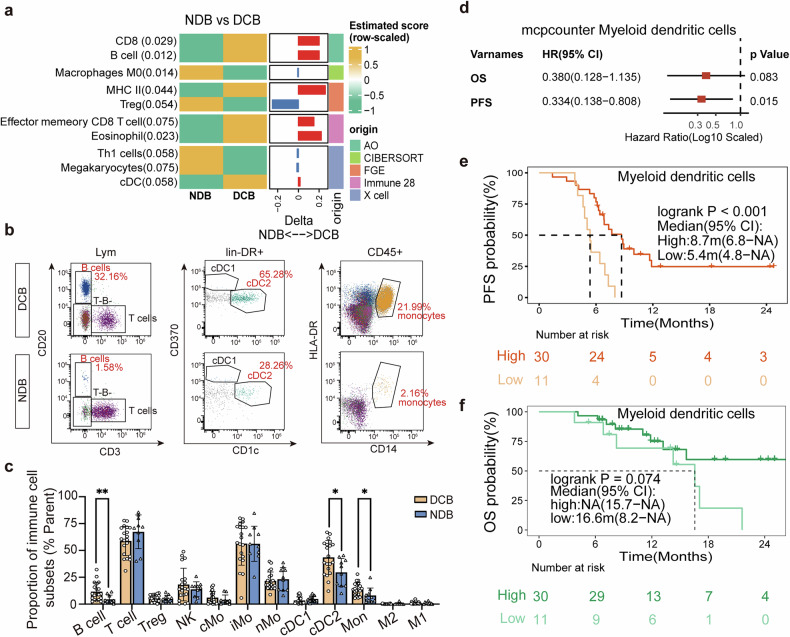
Comprehensive analysis of PBMCs. **a** Transcriptomic profiling of PBMCs distinguished DCB and NDB groups (*n* = 41). **b**, **c** Full spectrum flow cytometry verified the PBMC transcriptome results and found that compared with NDB, the DCB group had higher baseline B cells, cDC2 and monocytes. **d** Univariate COX regression analysis identified a significant association between myeloid dendritic cells and prognosis. **e**, **f** Kaplan–Meier survival curves for PFS and OS of myeloid dendritic cells

To further investigate whether potential biomarkers can be tested via non-invasive approach, we revealed systemic immune profile via a comprehensive transcriptomic analysis of baseline PBMCs (*n* = 41). Distinct transcriptional profiles that effectively distinguished the DCB group from the NDB group were disclosed. The DCB had enriched B cells, CD8^+^ T cells, MHC II, and dendritic cells (DCs), while NDB was enriched with macrophage and regulatory T cells (Treg) (Fig. 4a). Subsequently, a full spectrum flow cytometry panel was then designed to verify the above results (*n* = 30). Notably, patients in the DCB group exhibited elevated circulating DCs, B cells and monocytes consistent with PBMC transcriptomic results (Fig. 4b–c). Patients were stratified based on levels of DCs and B cells. B cell infiltration demonstrated no statistically significant association with prognosis (Supplementary Fig. 8a–c), while higher DC was correlated with improved PFS and a marginal benefit to OS (Low vs High, PFS: 5.4 m vs 8.7 m, *P* < 0.001; OS: 16.6 m vs not reached, *P* = 0.074, Fig. 4d–f). Although PBMC B cells, DCs and monocytes were correlated with ICI benefit, the predictive utility of systemic immune profiles might be constrained by the modest cohort size.

### Plasma proteomics identifies the ES-SCLC immune-benefit population

**Fig. 5:**
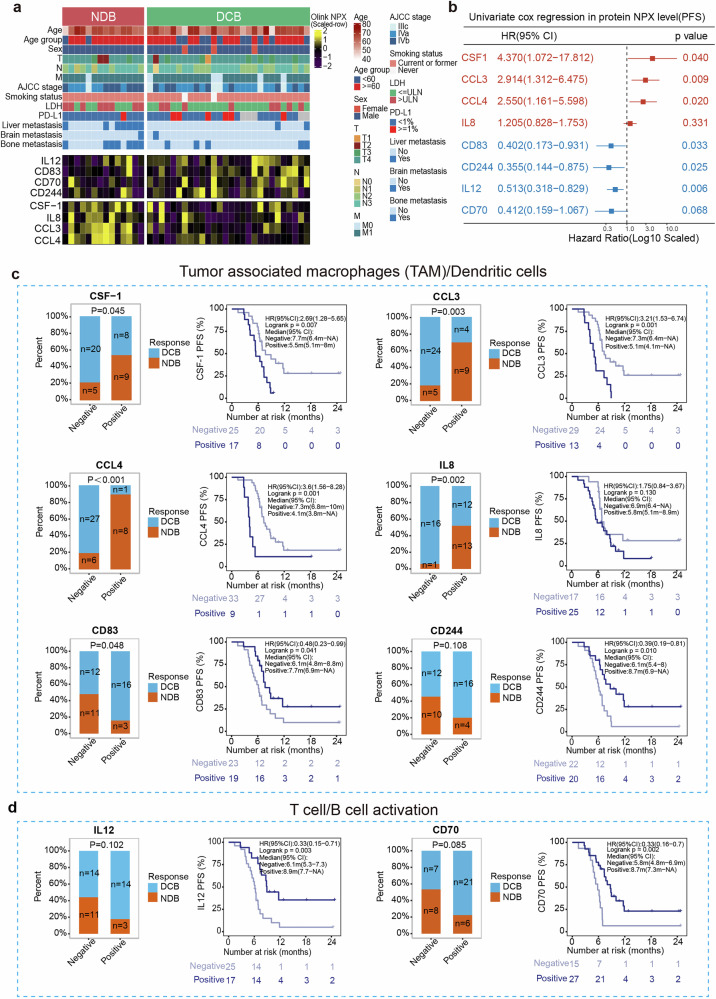
Comprehensive analysis of circulating plasma proteomes. **a** Olink proteomics revealed different factors between the DCB and NDB group. **b** Univariate Cox regression indicated that Olink proteomics associated with prognosis. **c** Circulating protein detection showed that the positive expression of CSF-1, CCL3/4, and IL8 correlates with increased NDB incidence, while CD83 and CD244 correlates with increased DCB. The Kaplan–Meier curves for PFS, based on circulating protein expression levels, distinctly differentiate the DCB from the NDB. **d** IL12 and CD70 are associated with increased DCB incidence. The Kaplan–Meier curves showed a high level of IL12 and CD70 were correlated with longer PFS

Next, we hypothesized that soluble proteins in plasma may represent cell functions in TME. Thus, the proteomics analysis of baseline plasma (*n* = 42) was conducted. The results revealed higher levels of IL-12, CD83, CD70, and CD244 in the DCB group, whereas CSF-1, IL-8, CCL3, and CCL4 were elevated in the NDB group (Fig. 5a). Univariate cox regression showed that higher peripheral circulating protein levels of CSF-1, CCL3 and CCL4 which are markers of M2 macrophages, were linked to a poorer prognosis (CSF-1: HR = 4.370, *P* = 0.04; CCL3: HR = 2.914, *P* = 0.009; CCL4: HR = 2.550, *P* = 0.020) (Fig. 5b). Meanwhile, CSF-1, CCL3, CCL4, and IL-8 expression were correlated with increased NDB incidence (*P* < 0.05) and worse PFS (CSF-1: HR = 2.69, *P* = 0.007; CCL3: HR = 3.21, *P* = 0.001; CCL4: HR = 3.6 *P* = 0.001; IL-8: HR = 1.75, *P* = 0.130) (Fig. 5c). In contrast, CD83 and CD244, markers of DCs, IL-12 and CD70, markers of T/B cell activation, were linked to DCB (CD83: *P* = 0.048; CD244: *P* = 0.108; IL-12: *P* = 0.102; CD70: *P* = 0.085) and longer PFS (CD83: HR = 0.48, *P* = 0.041; CD244: HR = 0.39, *P* = 0.01; IL-12: HR = 0.33, *P* = 0.003; CD70: HR = 0.33, *P* = 0.002) (Fig. 5c, d). Moreover, these eight proteins were linked with similar trends in OS (Supplementary Fig. 9a–h).

### Multi-omics non-invasive model construction and validation

**Fig. 6:**
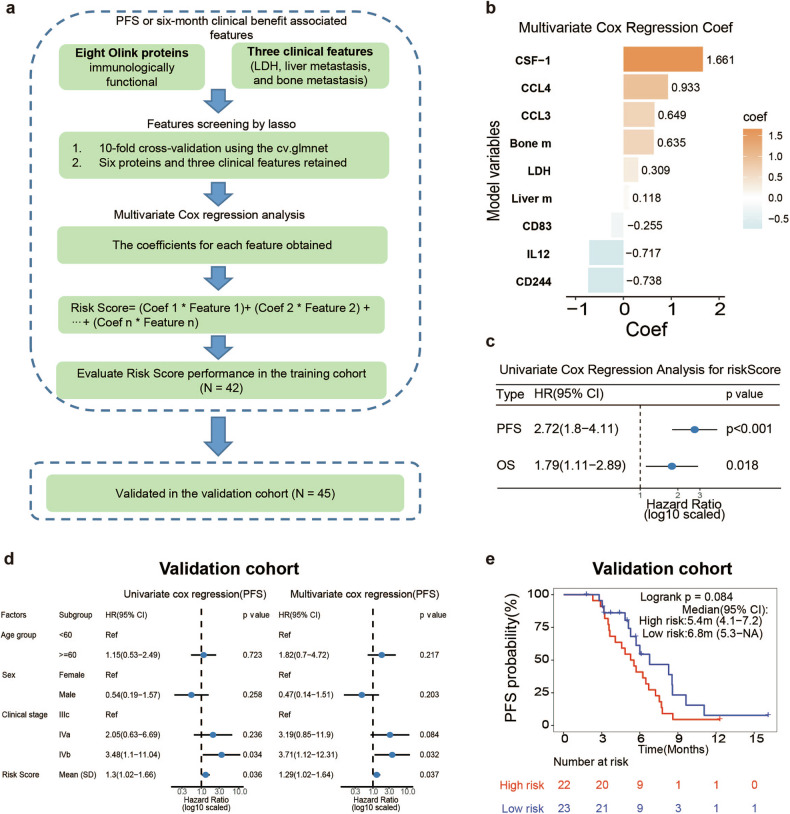
Multi-omics non-invasive model construction and validation**. a** Model diagram of construction and verification of multivariate Cox regression model based on LASSO. **b** Multivariate Cox regression Coef in model. **c** Univariate Cox regression indicated that risk score was associated with PFS and OS. **d** Univariate and multivariate Cox regression of PFS in validation cohort (*n* = 45). **e** Kaplan–Meier curves showed high risk (with median-cut-off value) was correlated with shorter PFS in the validation cohort

Subsequently, plasma proteomics and clinical variables were analyzed using LASSO regression to identify key features, which facilitated the development of a non-invasive predictive model for predicting clinical response to ICIs in ES-SCLC (Fig. 6a, Supplementary Fig. 10a-b). Six proteins (CSF-1, CCL4, CCL3, CD83, IL-12, and CD244) and three clinical characteristics (LDH levels, bone metastasis status, and liver metastasis status) were incorporated into the predictive model (Fig. 6b). Risk score was significantly associated with PFS (HR = 2.72, *P* < 0.001) and OS (HR = 1.79, *P* = 0.018) in the univariate COX regression analysis (Fig. 6c). The high-risk group identified by the model exhibited shorter PFS (High risk vs Low risk, PFS: 5.4 m vs 10.0 m, *P* < 0.001; Supplementary Fig. 10c-d) and OS (High risk vs Low risk, OS: 15.7 m vs not reached, *P* = 0.010; Supplementary Fig. 10e).

Subsequently, we included 45 real-world ES-SCLC patients to conduct an external validation of the aforementioned model (Supplementary Table 2). The results demonstrated that the model exhibited favourable predictive performance (Fig. 6d). The high-risk group identified by the model demonstrated a greater prevalence of patients in the NDB group (*P* = 0.006; Supplementary Fig. 10f), along with a shorter PFS with median cut-off value (High risk vs Low risk, PFS: 5.4 m vs 6.8 m, *P* = 0.084; Fig. 6e). Furthermore, the best cut-off value showed a similar tendency (High risk vs Low risk, PFS: 4.1 m vs 6.8 m, *P* = 0.001; Supplementary Fig. 10g).

## Discussion

In our study, the integration of sintilimab and chemotherapy exhibited a median PFS of 6.9 months, and PFS rate at 12 months was 16.9%, which met the primary endpoint of our design. Of note, the median OS was 17.1 months, with three patients completing the 2-years treatment and one patient achieving CR at the cut-off date, highlighting the potential of integrating sintilimab with chemotherapy as a novel therapeutic strategy. The treatment regimen was also well tolerated. Additionally, by using the multi-omics technique on baseline tumor and blood samples, we proposed novel immunologic characteristics of the immunotherapy response in SCLC.

Our study demonstrated a slightly longer median PFS and OS comparing with the published trials. We speculate that the limited sample size, along with the lower proportion of patients with liver metastases (20.9% in our study compared to 38% in IMpower133, 40.3% in CASPIAN, 25.4% in ASTRUM-005, 32% in CAPSTONE-1 and 28% in RATIONALE-312), may be potential contributing factors to the findings. Intriguingly, a study comparing continuous immunotherapy versus fixed-duration regimens in NSCLC lacked evidence for a statistically significant OS advantage.^16^ This finding partially corroborates our study design of 2-year immunotherapy, highlighting the need to further refine maintenance duration in SCLC.

While combination chemo-immunotherapy with maintenance emerged as cornerstone therapeutic approach for ES-SCLC, sustained survival improvements occur in only a minority of cases. Enhancing immunotherapy outcomes necessitates accurate selection of treatment responders. Our multi-omics biomarker profiling using baseline samples deciphers ES-SCLC’s biological complexity.

The unique immune microenvironment of ES-SCLC modulates therapeutic efficacy to ICIs. Within the local microenvironment, studies have documented the populations of T cells and M2 macrophages possess predictive value for immunotherapy efficacy in ES-SCLC.^17^ More importantly, our study identified subpopulations of T cells and macrophages with distinct roles in anti-tumor immunity, proposing them as potential biomarkers for predicting treatment outcomes. Our study revealed significant enrichment of CXCR5^+^CD4^+^ and CXCR5^+^CD8^+^ T cell in ES-SCLC patients exhibiting robust immunotherapy responses, suggesting their cooperative roles in shaping anti-tumor immunity. Notably, CXCR5^+^CD4^+^ T cells (T follicular helper cells, Tfh),^18^ known to orchestrate B cell maturation and germinal center reactions across multiple malignancies, may synergize with their CXCR5^+^CD8^+^ counterparts (follicular cytotoxic T cells) to establish a coordinated immune niche.^19^ While Tfh cells potentially facilitate tertiary lymphoid structure (TLS) formation and humoral immunity, CXCR5^+^CD8^+^ T cells likely contribute direct tumoricidal effects through cytotoxic granule release and IFN-γ production.^20^ In our study, the presence of CXCR5^+^ cells may reflect enhanced tumor-infiltrating lymphocyte (TIL) activity and the formation of TLS, both of which are associated with robust anti-tumor immunity. These findings align with previous studies demonstrating the role of CXCR5 in promoting immune cell recruitment and coordination within the TME.^21,22^ CD103^+^CD8^+^ T cells, which function as tissue-resident memory T cells, have been determined to predict prognostic value of ICIs in various solid tumors as well.^23^ Analysis revealed elevated infiltration of CD103^+^ CD8^+^ T cells in responders, underscoring their functional contribution to intratumoral immune activity. In addition, B cell related genes were enriched in the tissues of responding patients. An elevated B cell population was observed in baseline tumor tissue and peripheral blood. B cells were already known key components of TLS and positively correlated with patients’ response to immunotherapy.^24^ Furthermore, CD200 was associated with a better prognosis for immunotherapy. Two positive cells, CD200^+^ B cells and CD200^+^ CXCR5^+^T cells were observed in the DCB group. CD200 was known to be expressed in B cells^25,26^, which was used as a B cell marker in our study. CD200 also expressed in T cells, and played a crucial role in immunotherapy by secreting effector molecules, which contribute to improved anti-tumor activity.^27^ Thus, CD200^+^ CXCR5^+^ T cells identified in our study may demonstrate enhanced immunity potentially contributed to the response to immunotherapy.

M2-like macrophages are believed to be associated with poor responses to ICIs,^28^ our study revealed a detailed phenotype of M2-like TAMs. In addition to CD163, a conventional marker of the M2 type, these cells often exhibited higher expression of CSF1R and SIGLEC5. CSF1R, binding to the ligands CSF-1, promotes the accumulation of TAMs with an immunosuppressive phenotype,^29,30^ mediating tumor progression and therapeutic resistance. As a constituent of the sialic acid-binding immunoglobulin-like lectins (SIGLECs) family, SIGLEC5 predominantly localizes to myeloid cell surfaces, notably macrophages. As an inhibitory receptor, SIGLEC5 engagement by sialylated ligands can inhibit pro-inflammatory responses,^31^ promoting a more immunosuppressive environment. This may help tumors evade immune surveillance by reducing macrophage-mediated phagocytosis and cytokine production.^32^ Our findings delineates critical associations between the tumor immune landscape and therapeutic responsiveness in ES-SCLC, highlighting the clinical relevance of immunophenotypic diversity. The marked enrichment of SIGLEC5^+^ and CSF1R^+^ M2-polarized macrophages within the NDB cohort demonstrates their immunosuppressive function. These findings collectively highlight blocking CSF-1/CSF1R axis may be an effective strategy to circumvent immunotherapy resistance in ES-SCLC.^33,34^

Beyond characterizing the local immune microenvironment, baseline elevations in B lymphocytes, DCs and monocytes of PBMC were displayed in DCB patients. These findings proposed a systemic-peripheral immune repertoire that orchestrated antitumor immunity. Thus, building upon previous acknowledgement of immune cell populations with distinct contributions to ICI efficacy, eight plasma proteins engaging immune cell activation, chemotaxis, and differentiation were identified. Furthermore, a non-invasive immune efficacy prediction model for ES-SCLC patients was developed and validated integrating levels of plasma proteins and efficacy-related clinical features. The model included six plasma proteins including CSF-1, CCL4, CCL3, CD83, IL-12, and CD244. Chemokines (CCL3/4) and cytokines (CSF-1) involved in monocyte recruitment and M2-TAM maturation, respectively, were associated with unflavoured response to ICIs.^35,36^ The expression of CD244 has been shown on the surface of tumor-infiltrating monocytes, and it is associated with an immunosuppressive phenotype.^37^ These CD244 expressing monocytes may be recruited to tumor tissues in response to chemokines like CSF-1, CCL3/4. And CD83-mediated activation of lymphocyte and DCs may indicate better response to the ICIs.^38,39^ IL-12, as a highly effective pro-inflammatory cytokine, can enhance immune response, increasing the level of immune infiltration.^40^ The association between the risk score of the model and treatment outcomes, as observed in our study, highlighting the potential of blood-based biomarkers in immuno-oncology.^41^

The study has some limitations. Firstly, the sample was small. And the single-center, single-arm trial, conducted exclusively in Chinese population with a relatively high proportion of male participants, inherently constrains the generalizability of results to broader populations, necessitating ongoing follow-up to validate long-term outcomes. The inherent susceptibility of blood-based biomarkers to confounding variables necessitates rigorous multi-cohort validation to disentangle predictive versus prognostic utility. And development of clinically accessible detection methods to complement the Olink-based plasma protein analysis employed in our study should also be conducted in the future. Moreover, due to the limited number of biopsies and difficulties in obtaining fresh tissue in clinical practice, single-cell sequencing could not be conducted, which was another limitation in this study. The incorporation of single-cell sequencing in future studies is warranted to further investigate the TME and enhance the robustness of our findings. Despite these limitations, our findings lay the groundwork for future research and highlight the need for continued exploration into personalized treatment strategies for SCLC.

Our study demonstrates the potential of sintilimab synergized with platinum-etoposide therapy as a front-line strategy for ES-SCLC, highlighting robust efficacy and manageable safety profiles. Biomarker analysis offers insights into the local and systemic immune landscapes of ES-SCLC. Multi-omics non-invasive model was constructed. Ongoing research is essential to refine biomarker-guided strategies and improve outcomes for patients with this aggressive malignancy.

## Materials and methods

### Study design and participants

This phase II trial (ChiCTR2000038354) employed an open-label, single-center design to quantify the efficacy, safety, and biomarkers associated with first-line sintilimab synergized with platinum-etoposide therapy in ES-SCLC.

Key inclusion criteria: 18–75 years old; treatment-naive ES-SCLC; at least one lesion measurable guided by Response Evaluation Criteria in Solid Tumors version 1.1 (RECIST v1.1); an ECOG-PS of 0–2; predicted life expectancy exceeding 3 months.

This trial adhered to Good Clinical Practice standards and conducted under the Declaration of Helsinki framework.

### Treatment, assessments and outcomes

Treatment was administered in every 21 days. Eligible patients received six cycles of platinum (cisplatin or carboplatin) and etoposide with sintilimab as an induction treatment. Then sintilimab was administered until the point of disease progression, unacceptable toxicity, or along with other protocol-specified discontinuation criteria.

Tumor imaging assessments were conducted at baseline, followed by 6-week intervals ( ± 7 days) during the initial 24-week phase, transitioning to 9-week intervals ( ± 7 days) thereafter. RECIST v1.1 was used to assess the tumor responses. Adverse events were recorded according to the National Cancer Institute Common Terminology Criteria for Adverse Events version 5.0 (NCI-CTCAE v5.0). Survival status was recorded every 90 days until patient death or the data cut-off.

1-year PFS rate was the primary endpoint; secondary outcomes comprised PFS, OS, ORR, and safety. The exploratory endpoint was biomarkers.

### Sample collection

The baseline formalin-fixed paraffin embedded (FFPE) tissue was provided by patients with their agreement. FFPE was used to extract DNA and RNA for sequencing and conduct mIF. Baseline blood samples were centrifuged to separate the plasma and PBMC for plasma proteomics, RNA sequencing and full spectrum FCM.

### Real-world validation cohort

We collected real-world cohorts of ES-SCLC who received first-line chemo-immunotherapy. We obtained two cohorts for validation: 1) real-world cohort 1: baseline tumor tissue samples from 24 patients for mIF, 2) real-world cohort 2: peripheral blood plasma samples from 45 patients for proteomic profiling. The protocol was approved by each center.

Patients diagnosed with ES-SCLC at Shanghai Pulmonary Hospital from June 2020 to April 2021 were retrospectively reviewed for real-world cohort 1. Enrolled criteria: (1) Histologically or cytologically confirmed ES-SCLC. (2) Front-line treatment was atezolizumab plus chemotherapy. (3) Complete clinical data. (4) Accessible baseline tissue specimens.

Patients diagnosed with ES-SCLC at other two medical centers from June 2022 to September 2024 were retrospectively reviewed for real-world cohort 2. The primary inclusion criteria for cohort 2 were similar to those of cohort 1, with the requirement for cohort 2 participants to provide baseline plasma samples rather than tissue.

### Whole-exome sequencing

Genomic DNA was isolated from buffy coat and FFPE specimen blocks using the AmoyDx Fresh/FFPE DNA Kits (Amoy Diagnostics, Xiamen, China). The concentration of extracted genomic DNA from FFPE tumor tissue was quantified. DNA libraries were constructed (Illumina document number: 15037436). The libraries were sequenced. Variants calling, annotation and filtration were conducted.

As for the TMB group, participants with a TMB exceeding the median threshold were classified as high TMB, while those at or below the median were classified as low TMB.

### RNA sequencing

RNA isolation from FFEP specimens and PBMCs was performed using AmoyDx RNA extraction kits (Amoy Diagnostics, China). RNA concentration and fragment length were assessed. Strand-specific libraries were prepared (Cat. #E7760L, NEB). Libraries were subsequently constructed with TruSeq RNA kit (Illumina) and sequenced.

### Gene expression estimation

The alignment process for paired-end RNA sequencing reads utilized the STAR aligner (v2.0.201) against the human reference genome GRCh37 (hg19), incorporating transcriptomic annotations provided by Gencode release 20. Reads from coding regions were converted to TPM and log2 transformed for further analysis.

### Gene expression and functional enrichment analysis

Differential gene expression (DEG) analysis was performed with DESeq2 (v1.38.3) after filtering low-expression genes (total counts ≤ 1 across samples). The DESeq function incorporated size factor normalization and negative binomial modeling to identify DEGs. DEGs were defined based on two criteria: 1) statistical significance (*p*-value < 0.05) and 2) an absolute fold change greater than 1.5.

Functional enrichment of DEGs was assessed via clusterProfiler (adjusted *P* < 0.05), and gene set variation analysis (GSVA) calculated pathway activity scores using log2(TPM + 1) matrices.

### In silico evaluation of tumor microenvironment

Multiple TME signatures, comprising 29 gene expression, 28 immune gene sets, Danaher, and AO, were quantified using GSVA. MCPcounter and xCell score were calculated with R packages.

Additionally, CIBERSORT was employed to assess immune cell infiltrations utilizing the leukocyte gene signature matrix LM22.^42^

### SCLC subtype

For classic subtype analysis, gene expression of ASCL1, NUEROD1, YAP1, and POU2F3 genes were used for hierarchical clustering. Furthermore, the previously reported 1300 NMF-derived signature genes were also used to hierarchically cluster the present gene expression matrix. The hierarchical clustering of our matrix using key transcription factors and APM from newly published research^23^ was also performed for subtyping. The gene expression data were derived from a log2-transformed TPM matrix.

### Plasma proteomics assay

Plasma levels of 92 human protein biomarkers were quantified using Olink Immuno-Oncology I panel. Specifically, paired antibody-conjugated oligonucleotides bind target proteins, triggering DNA hybridization and polymerase-mediated extension to generate PCR-amplifiable sequences, which were quantified by microfluidic PCR.

The assay quantified protein expression levels using normalized log_2_-transformed arbitrary units, with elevated values corresponding to increased protein abundance. Receiver operating characteristic (ROC) analysis was conducted on normalized Olink protein expression values for the 6-month DCB and NDB groups to identify the cut-off that maximizes the Youden index for patient classification.

### Feature selection and model construction

Eight immunologically relevant proteins and three clinical features (LDH, metastasis status of liver and bone), which were associated with PFS survival or six months clinical benefit, were initially included. Feature selection was conducted using least absolute shrinkage and selection operator (LASSO) regression with 10-fold cross-validation. The optimal tuning parameter (λ) was determined based on the minimum partial likelihood deviance. This process retained six proteins and all three clinical features for further analysis.

Selected features were analyzed using multivariate Cox regression to assess prognostic value.

$${\rm{R}}{\rm{i}}{\rm{s}}{\rm{k}}\,{\rm{S}}{\rm{c}}{\rm{o}}{\rm{r}}{\rm{e}}=\mathop{\sum }\limits_{i=1}^{n}{Coe\,f}_{i}\cdot {Feature}_{i}$$

where Coef represents the regression coefficient derived from the Cox model, and Feature corresponds to the standardized value of each selected biomarker or clinical variable. Patients were categorized into different groups based on the median cutoff in discovery cohort.

The prognostic performance was assessed using univariate and multivariate Cox regression. Kaplan–Meier analysis with log-rank tests was performed in discovery cohort. The risk score’s stability was verified through external assessment in a distinct validation cohort.

### Multiplex immunofluorescence

For clinical trial samples, three panels were used. Panel 1 evaluated T cell subpopulations containing CD4, IRF4, CXCR5, CD8, and CD103. Panel 2 evaluated T/B cell populations including CD20, CD200, and CXCR5. Panel 3 evaluated TAM consisting of CSF-1R, SIGLEC5, CD68, and CD163. In the validation cohort, besides panel 3, panel 4 of T and B cells containing CD4, CD8, CD20, CXCR5, and CD103 was also applied.

Tissue sections were prepared. After blocking with commercial buffer, sequential immunostaining was performed using pre-optimized antibody concentrations and sequences (Supplementary Table 3). Nuclei were counterstained with DAPI. Two blinded pathologists independently evaluated all specimens.

### Full spectrum flow cytometry

PBMC immunophenotyping was conducted via spectral FCM (Cytek NL-CLC) using a 19-marker antibody panel designed from PBMC RNA-seq data. Specifically, Fc receptor-block solution was applied, followed by washing of the samples. Fixable Viability Stain 700 was then used on PBMCs to distinguish live from dead cells prior to analyses. PBMCs were incubated with antibodies. After incubation, the samples were washed twice and resuspended. The immune cells were analyzed by manual gating using FlowJo software. The percent of the parent population was used to quantify subsets. The panel was listed in Supplementary Table 4.

### Statistical analysis

The previous study showed that 12-month PFS rate was 5.4% in ES-SCLC receiving chemotherapy. To increase the 12-month PFS rate to 15%, 45 patients was required. The specific assumptions were as follows: 1) α = 0.05 and power = 80%; 2) dropout rate of 10%.

Efficacy assessments included patients who completed at least two treatment cycles and underwent efficacy evaluation, while safety evaluations encompassed all patients receiving a minimum of one therapeutic cycle.

The logistic regression analysis was conducted to evaluate the correlation between continuous biomarker and six-month clinical benefit. Survival outcomes (PFS/OS) were estimated via Kaplan-Meier analysis, with subgroup comparisons using log-rank tests and Cox regression. Continuous variables were analyzed with Mann-Whitney *U* tests; categorical variables were compared through Chi-square test or Fisher’s exact testing. A two-sided *p*-value of <0.05 was considered statistically significant. All CI were reported at the 95% confidence level. Statistical analysis was performed using SPSS version 22.0 (SPSS Inc., Chicago, IL, USA) or R software (v 4.2.2).

## Supplementary information

Supplementary Materials

Study Protocol

## Data Availability

Genomic sequencing data have been submitted to the China National Center for Bioinformation (https://www.cncb.ac.cn/, ID: HRA011054). The other data reported have been deposited in the OMIX, China National Center for Bioinformation / Beijing Institute of Genomics, Chinese Academy of Sciences (https://ngdc.cncb.ac.cn/omix: accession no. OMIX009794, OMIX009796, OMIX009797, and OMIX009798). Clinical data were available upon reasonable request to the corresponding author.
