# Supplementary material for: Harnessing local and system immune profiling delineating differential responders to first-line sintilimab (anti-PD-1 antibody) combined with chemotherapy in extensive-stage small cell lung cancer: an exploratory biomarker analysis of a phase II study
Source: Signal Transduct Target Ther. 2025 May 23;10:168. doi: 10.1038/s41392-025-02252-5 (PMC12098833; doi:10.1038/s41392-025-02252-5)
Supplement: Supplementary file 1 — Supplementary Materials [file 41392_2025_2252_MOESM1_ESM.docx]

**Supplementary Materials**

Harnessing local and system immune profiling delineating differential responders to first-line sintilimab (anti-PD-1 antibody) combined with chemotherapy in extensive-stage small cell lung cancer: an exploratory biomarker analysis of a phase II study

Mengqing Xie*, Minwei Bao*, Xiaorong Dong*, Lin Wu*, Li Liu, Jing Zhao, Xiangling Chu, Yan Wu, Xianxiu Ji, Yujia Fang, Xin Yu, Shiji Zhang, Qi Wang, Tao Hu, Jin Wang, Changbin Zhu, Chunxia Su^#^

Correspondence to: susu_mail@126.com

**This PDF file includes:**

Supplementary Figures 1 to 10

Supplementary Tables 1 to 4

**
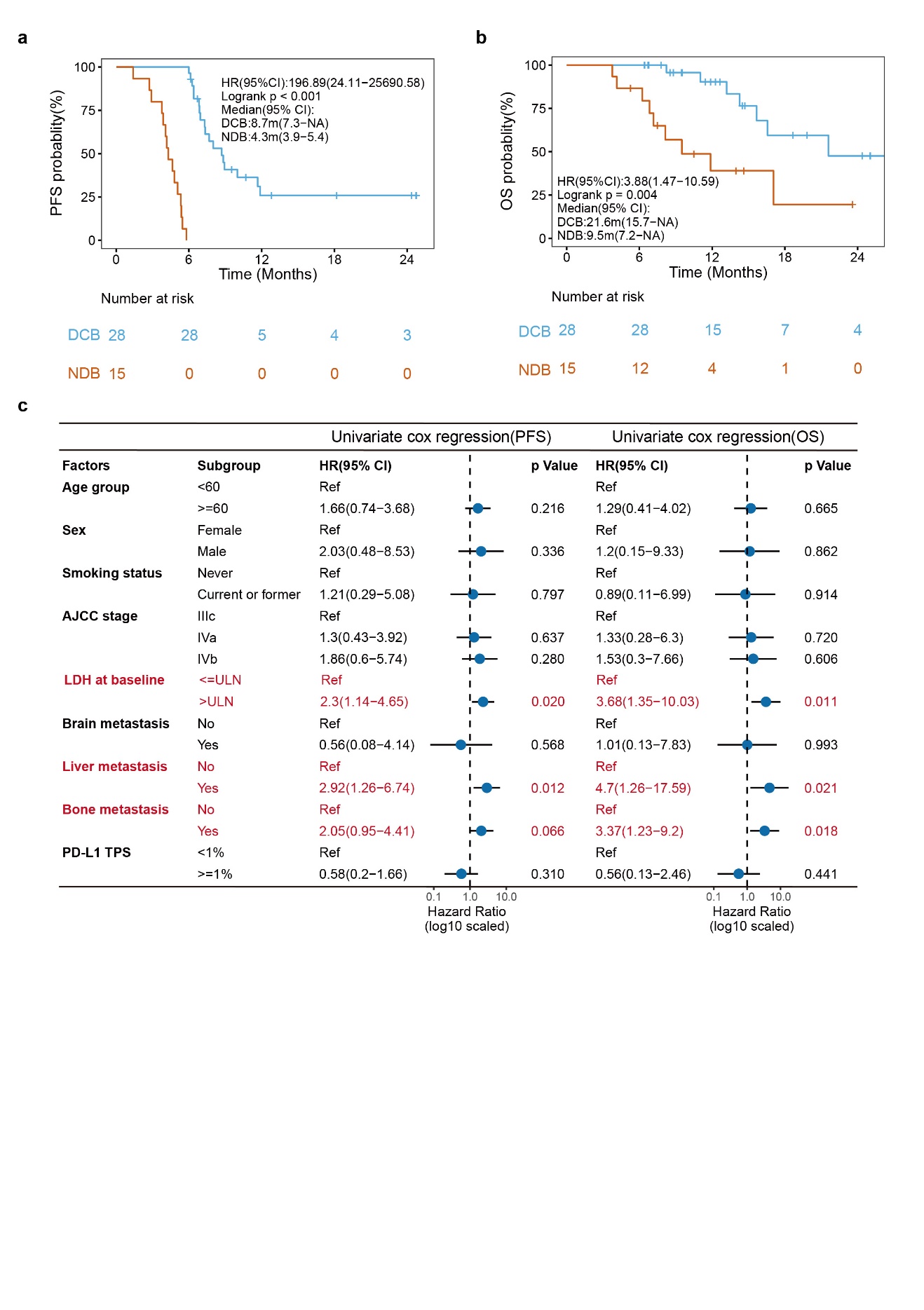
**

**Supplementary Figure 1.** **Analysis of clinical parameters on PFS and OS**

**a-b**, PFS and OS between DCB group and NDB group. **c**, Univariate COX regression analysis of clinical parameters on PFS and OS.

**
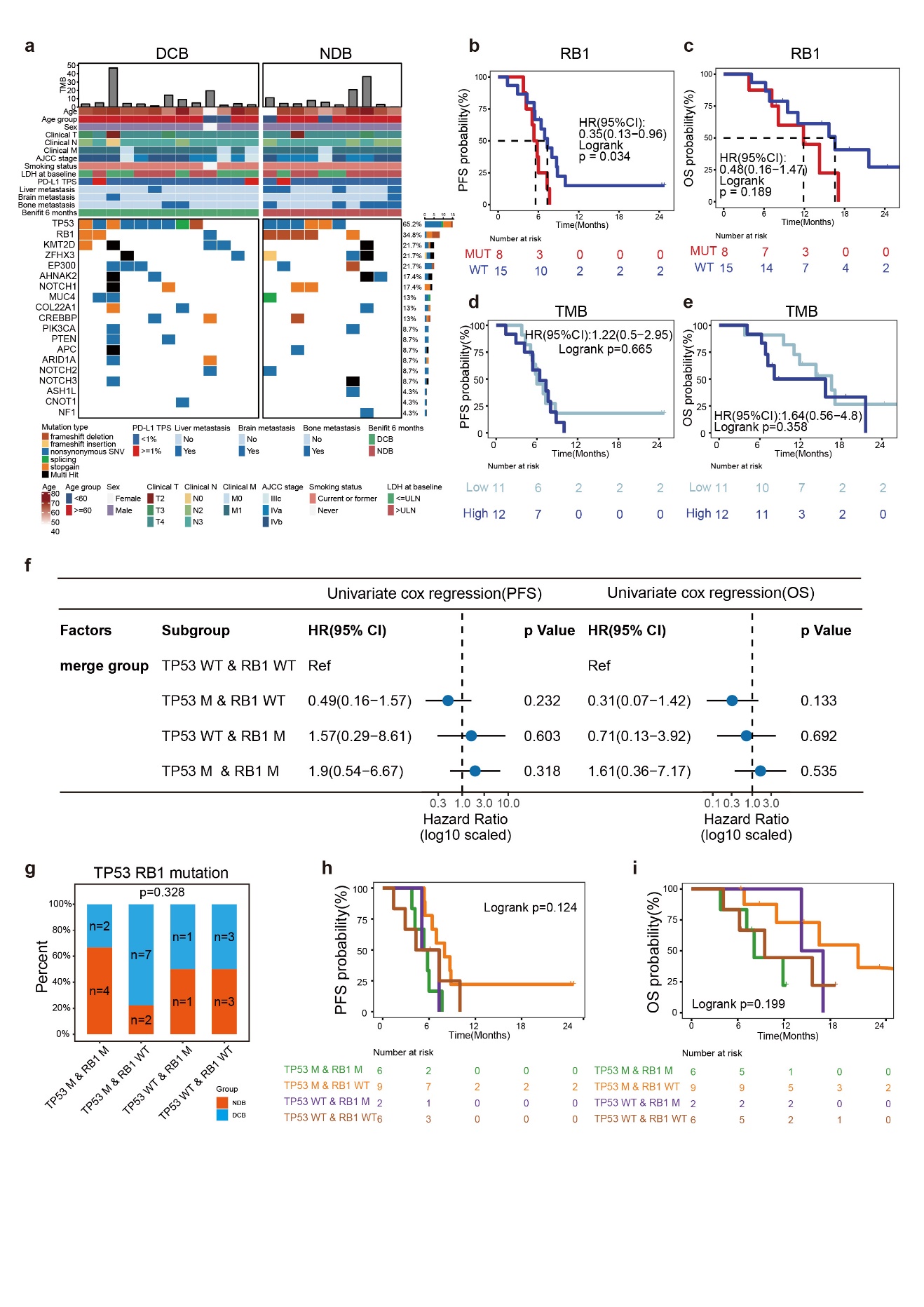
**

**Supplementary Figure 2. The landscape of genomic alterations and prognostics**

**a,** Landscape of genomic alterations in baseline tumor biopsy using WES. **b-c,** Kaplan-Meier curves for PFS and OS by *RB1* mutations. **d-e**, Kaplan-Meier curves for PFS and OS by TMB. **f**, Univariate COX regression analysis of PFS and OS, stratified by *TP53* and *RB1* mutation status. **g,** Analysis of the proportions of DCB and NDB across four mutation clusters, defined by the mutation status of *TP53* and *RB1*. **h-i,** Kaplan-Meier curves for PFS and OS by *RB1*/*TP53* mutations.

**
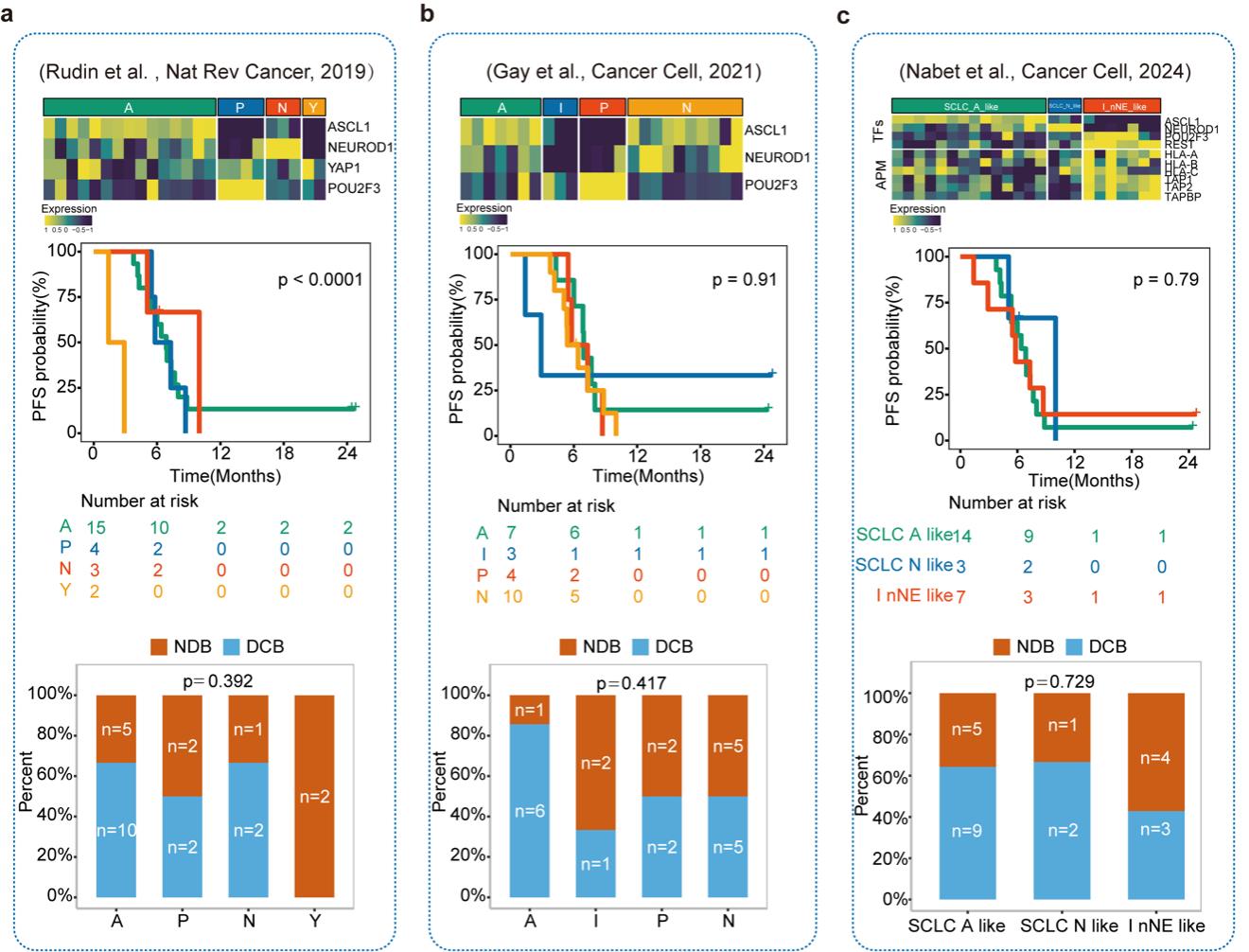
**

**Supplementary Figure 3.** **SCLC molecular subtyping and prognostics**

**a-c,** Validation of SCLC molecular subtypes and prognosis in our cohort according to published studies.

**
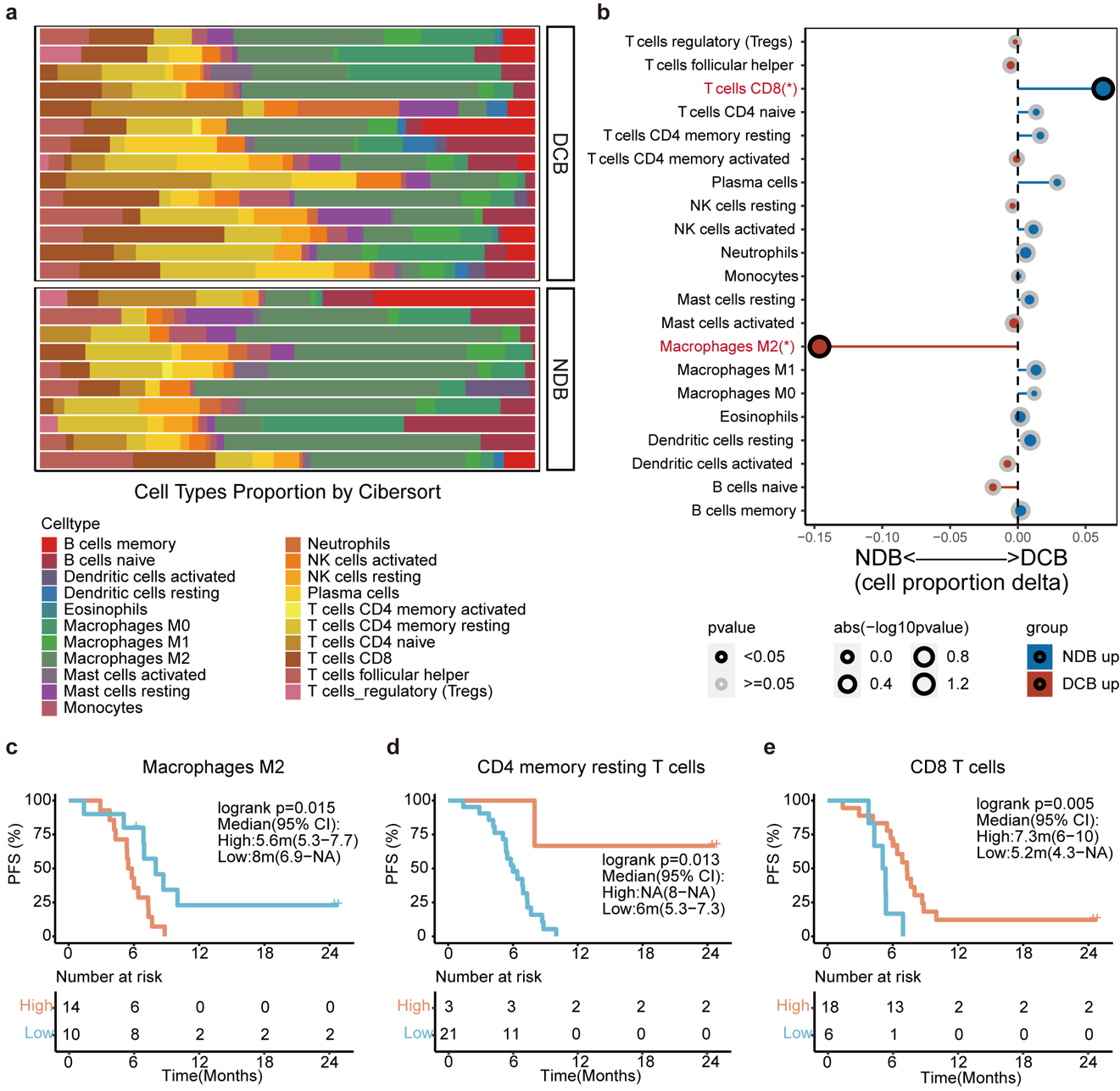
**

**Supplementary Figure 4.** **Significantly differentiated tumor microenvironment components and their correlation with PFS**

**a,** Tumor microenvironment revealed by CIBERSORT analysis**. b,** CIBERSORT analysis showing significant enrichment of CD8^+^ T cells in the DCB group and M2 macrophages in the NDB group. **c-e,** Kaplan-Meier curves of PFS for M2 macrophages, CD4^+^ memory resting T cells, CD8^+^ T cells and prognosis

**
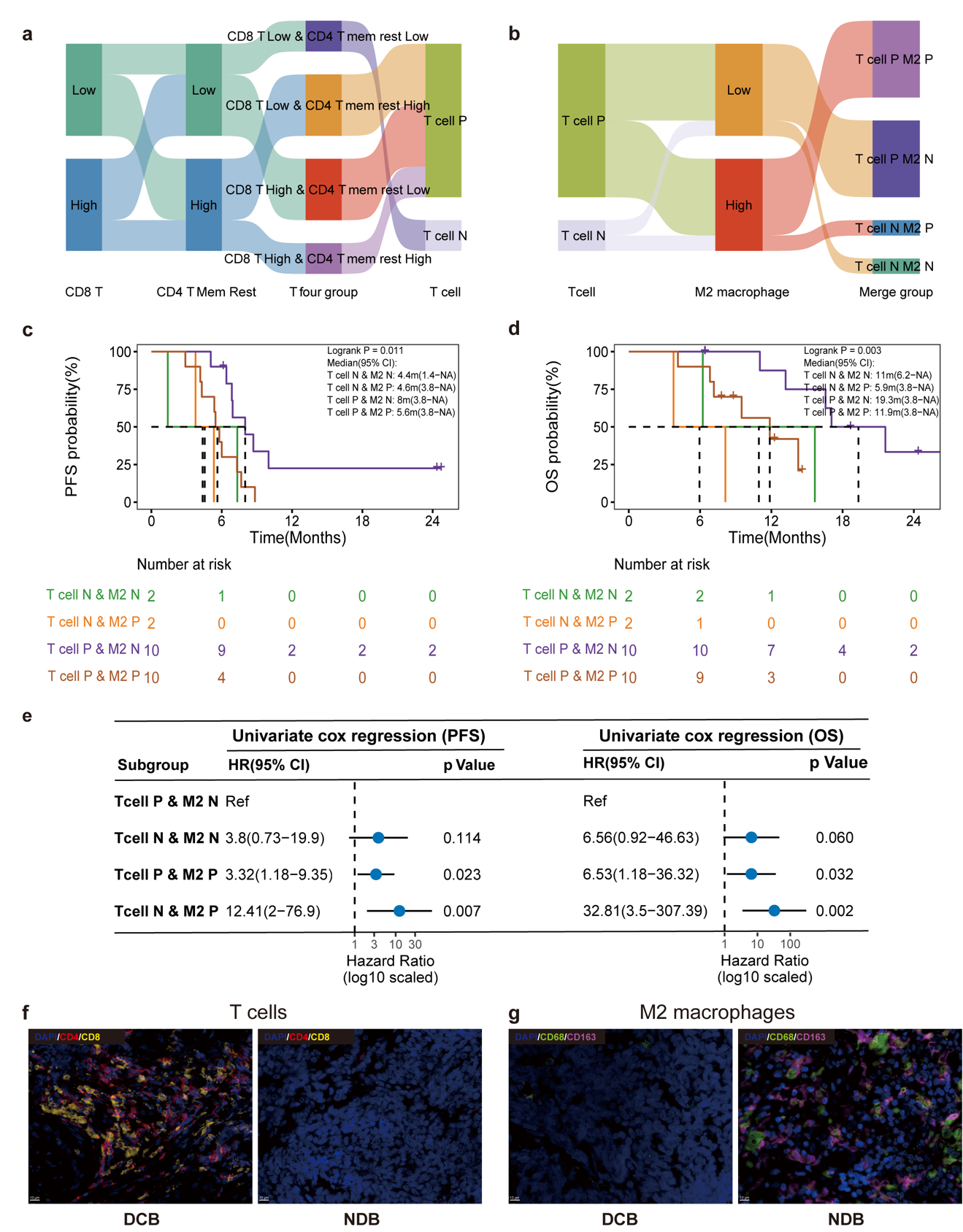
**

**Supplementary Figure 5. T cells combination macrophages signatures impacting immunotherapy efficacy in SCLC**

**a**, Schematic diagram of T cells grouping. **b**, Schematic diagram of macrophages grouping. **c-d,** Kaplan-Meier curves revealing that the TME characterized by T cells positive and macrophage negative were significantly correlated with better PFS and OS. **e,** Univariate COX regression analysis of PFS and OS, stratified by different T cell and M2 macrophage groups. **f-g**, Representative images of mIF staining for T cell and macrophages in DCB and NDB groups; scale bar: 10 μm.

**
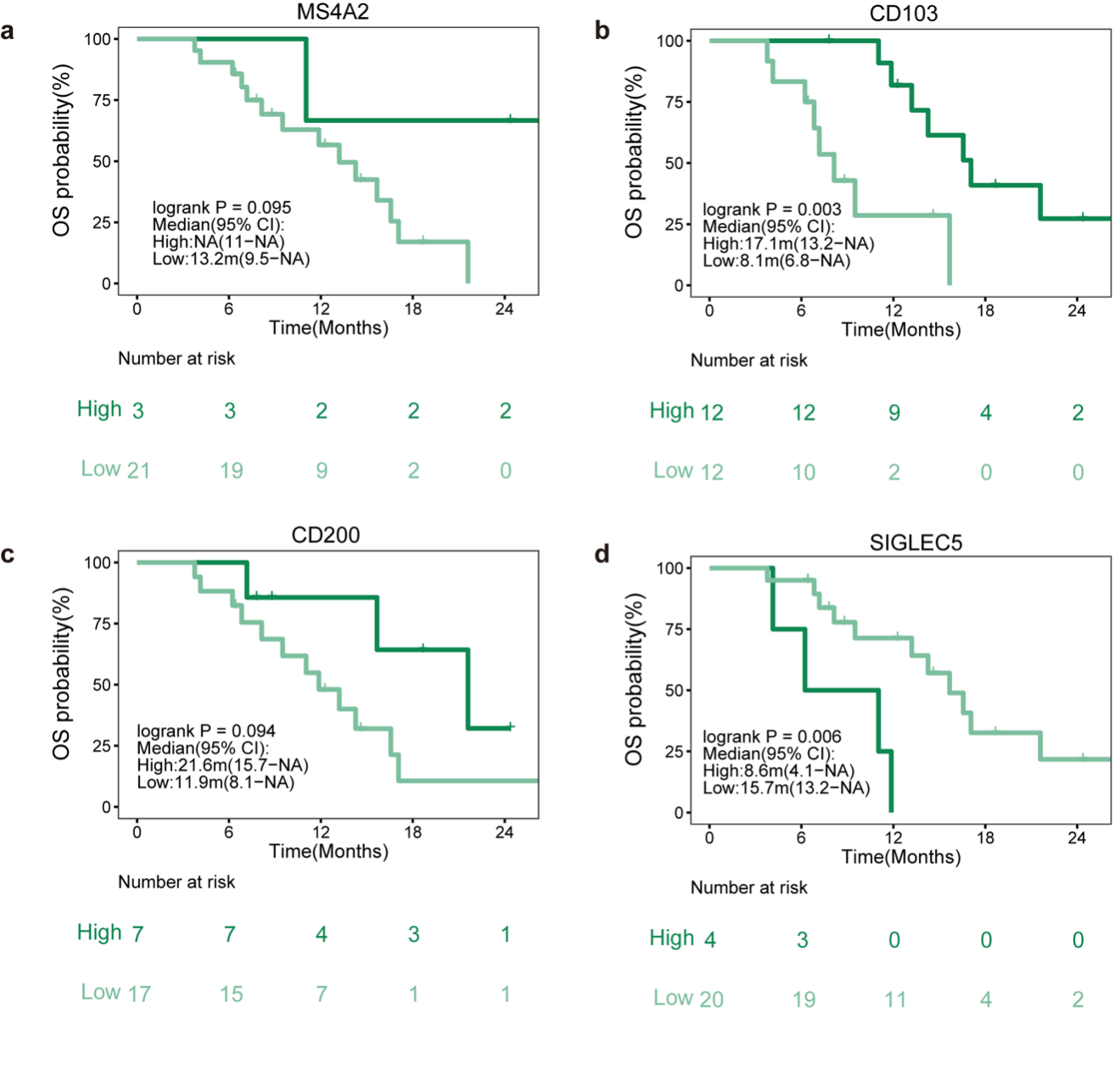
**

**Supplementary Figure 6. Analysis of TME components showed differentiation between DCB and NDB**

**a-d,** Kaplan-Meier curves of OS for *MS4A2*, *CD103*, *CD200* and *SIGLEC5*.

**
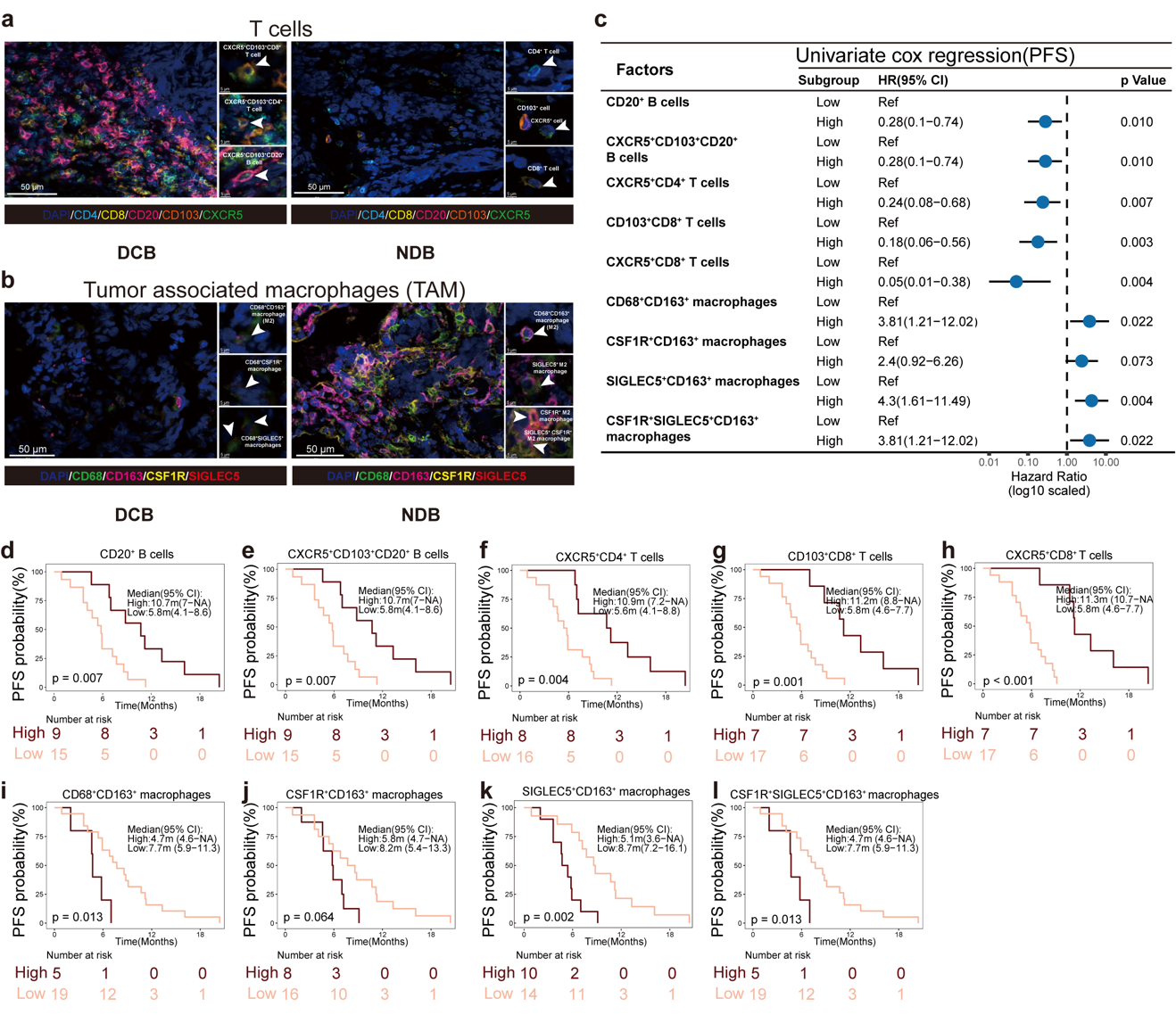
**

**Supplementary Figure 7. Correlation between TME and prognosis in a real-world validation cohort**

**a-b,** Representative images of mIF staining for T cell, and TAMs panel in DCB and NDB groups; scale bars: 50 μm (left); 5 μm (right). **c,** Univariate Cox regression analysis. **d-l,** Kaplan-Meier curves for PFS.


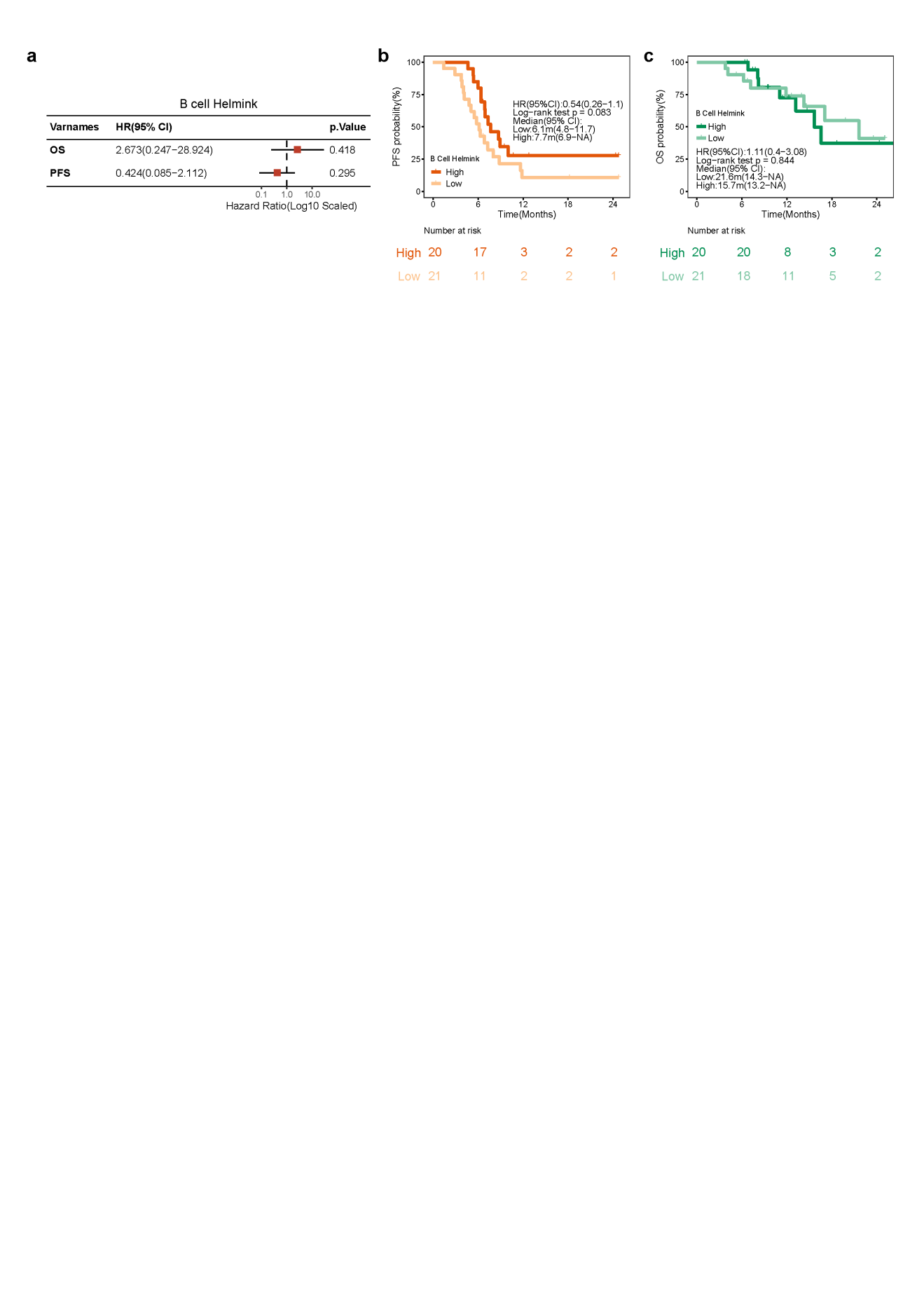


**Supplementary Figure 8. Peripheral blood RNA-seq suggests that B cells are not associated with SCLC prognosis**

**a,** Univariate COX regression analysis identified an association between B cell Helmink and prognosis. **b-c,** Kaplan-Meier curves of PFS and OS for B cell Helmink.

**
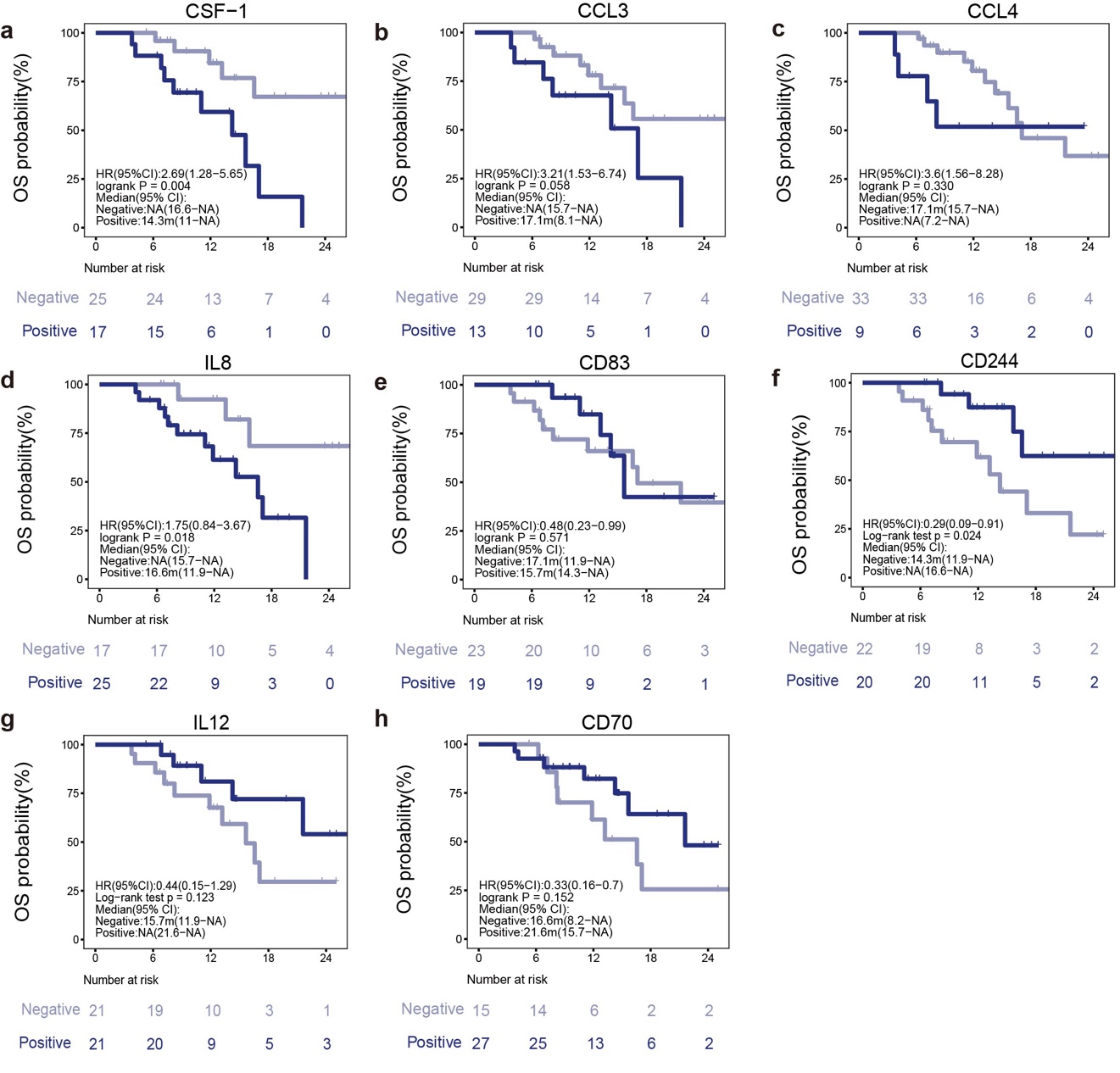
**

**Supplementary Figure 9. Prognostic significance of plasma proteomics**

**a-h,** Kaplan-Meier curves of OS for CSF-1, CCL3, CCL4, IL8, CD83, CD244, IL12, and CD70.


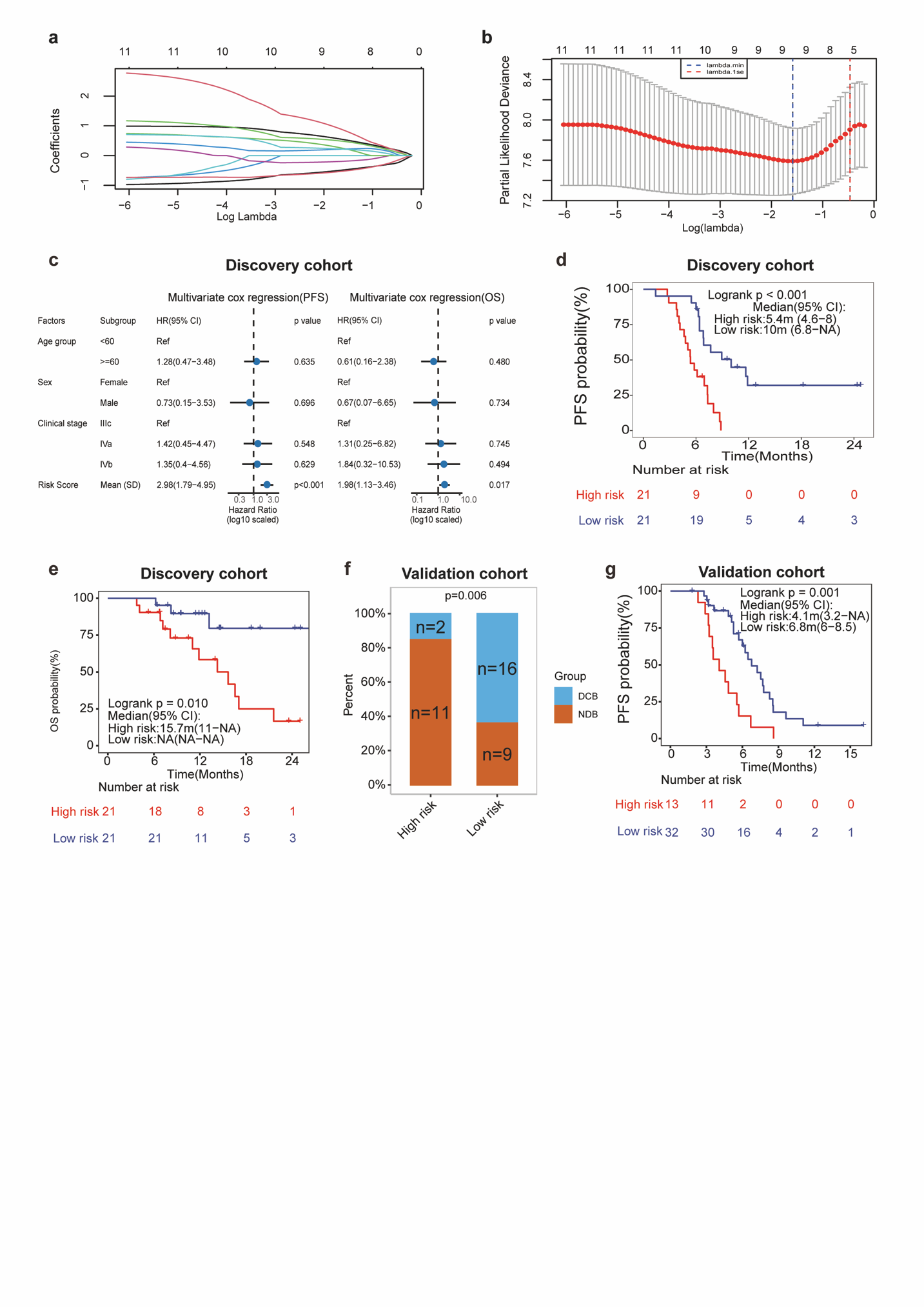


**Supplementary Figure 10. Feature selection using LASSO**

**a-b,** LASSO coefficient profiles and selection of tuning parameter (λ) in the LASSO regression. **c**, Multivariate Cox regression of PFS and OS in discovery cohort (n = 42). **d-e**, Kaplan–Meier curves showed high risk (with median-cut-off value) was correlated with shorter PFS and OS in the discovery cohort. **f**, Analysis of the proportions of DCB and NDB across high risk and low risk in validation cohort. **g**, Kaplan-Meier curves of PFS for model in validation cohort (with best cut-off value).

| **Supplementary Table 1. Comparison of clinicopathological characteristics between IIT cohort and real-world cohort 1** | | | | |
| --- | --- | --- | --- | --- |
| **Characteristic** |  | **IIT cohort (n=43)** | **Real-world cohort 1 (n=24)** | **p** |
| **Age, years** |  |  |  |  |
| Median age (IQR) |  | 65 (60-69) | 65 (59-69) | 0.880 |
| Age group |  |  |  | 0.927 |
|  | < 65 years | 21 (48.8) | 12 (50.0) |  |
|  | ≥ 65 years | 22 (51.2) | 12 (50.0) |  |
| **Sex** |  |  |  | 1.000 |
|  | Male | 40 (93.0) | 22 (91.7) |  |
|  | Female | 3 (7.0) | 2 (8.3) |  |
| **ECOG performance status** |  |  |  | 0.358 |
|  | 0 | 0 (0.0) | 1 (4.2) |  |
|  | 1 | 43 (100.0) | 23 (95.8) |  |
| **Smoking status** |  |  |  | 1.000 |
|  | Never | 2 (4.7) | 1 (4.2) |  |
|  | Current or Former | 41 (95.3) | 23 (95.8) |  |
| **AJCC stage^8th^** |  |  |  | 0.110 |
|  | IIIc | 6 (14.0) | 3(12.5) |  |
|  | IVa | 21 (48.8) | 6 (25.0) |  |
|  | IVb | 16 (37.2) | 15(62.5) |  |
| **LDH at baseline** |  |  |  | 0.396 |
|  | ≤ ULN | 26 (60.5) | 17 (70.8) |  |
|  | > ULN | 17 (39.5) | 7 (29.2) |  |
| **Brain metastasis** |  |  |  | 0.097 |
|  | Yes | 2 (4.7) | 5 (20.8) |  |
|  | No | 41 (95.3) | 19 (79.2) |  |
| **Liver metastasis** |  |  |  | 0.596 |
|  | Yes | 9 (20.9) | 3 (12.5) |  |
|  | No | 34 (79.1) | 21 (87.5) |  |
| **Bone metastasis** |  |  |  | 0.793 |
|  | Yes | 13 (30.2) | 8 (33.3) |  |
|  | No | 30 (69.8) | 16 (66.7) |  |
| **Treatment strategies** |  |  |  | **< 0.001** |
|  | Sintilimab | 43 (100.0) | 0 (0.0) |  |
|  | Atezolizumab | 0 (0.0) | 24 (100.0) |  |
| **mPFS (95%CI)** |  | 6.9 (6.1-7.7) | 6.8 (5.5-8.2) | 0.441 |

Abbreviations: IQR, Interquartile Range; ECOG, Eastern Cooperative Oncology Group; AJCC, American Joint Committee on Cancer; LDH, lactate dehydrogenase; ULN, upper limit of normal; PFS, Progression Free Survival

| \| **Supplementary Table 2. Comparison of clinicopathological characteristics between IIT cohort and real-world cohort 2** \| \| \| \| \| \| --- \| --- \| --- \| --- \| --- \| \| **Characteristic** \|  \| **IIT cohort (n=43)** \| **Real-world cohort 2 (n=45)** \| **p** \| \| **Age, years** \|  \|  \|  \|  \| \| Median age (IQR) \|  \| 65 (60-69) \| 66 (59-69) \| 0.818 \| \| Age group \|  \|  \|  \| 0.680 \| \|  \| < 65 years \| 21 (48.8) \| 20 (44.4) \|  \| \|  \| ≥ 65 years \| 22 (51.2) \| 25 (55.6) \|  \| \| **Sex** \|  \|  \|  \| 0.528 \| \|  \| Male \| 40 (93.0) \| 39 (86.7) \|  \| \|  \| Female \| 3 (7.0) \| 6 (13.3) \|  \| \| **ECOG performance status** \|  \|  \|  \| **< 0.001** \| \|  \| 0 \| 0 (0.0) \| 15 (33.3) \|  \| \|  \| 1 \| 43 (100.0) \| 27 (60.0) \|  \| \|  \| 2 \| 0 (0.0) \| 3 (6.7) \|  \| \| **Smoking status** \|  \|  \|  \| 0.109 \| \|  \| Never \| 2 (4.7) \| 8 (17.8) \|  \| \|  \| Current or Former \| 41 (95.3) \| 37 (82.2) \|  \| \| **AJCC stage^8th^** \|  \|  \|  \| 0.143 \| \|  \| IIIc \| 6 (14.0) \| 7 (15.6) \|  \| \|  \| IVa \| 21 (48.8) \| 13 (28.9) \|  \| \|  \| IVb \| 16 (37.2) \| 25 (55.6) \|  \| \| **LDH at baseline** \|  \|  \|  \| 0.195 \| \|  \| ≤ ULN \| 26 (60.5) \| 21 (46.7) \|  \| \|  \| > ULN \| 17 (39.5) \| 24 (53.3) \|  \| \| **Brain metastasis** \|  \|  \|  \| 0.109 \| \|  \| Yes \| 2 (4.7) \| 8 (17.8) \|  \| \|  \| No \| 41 (95.3) \| 37 (82.2) \|  \| \| **Liver metastasis** \|  \|  \|  \| 0.192 \| \|  \| Yes \| 9 (20.9) \| 15 (33.3) \|  \| \|  \| No \| 34 (79.1) \| 30 (66.7) \|  \| \| **Bone metastasis** \|  \|  \|  \| 0.890 \| \|  \| Yes \| 13 (30.2) \| 13 (28.9) \|  \| \|  \| No \| 30 (69.8) \| 32 (71.1) \|  \| \| **Treatment strategies** \|  \|  \|  \| **< 0.001** \| \|  \| Toripalimab \| 0 (0.0) \| 14 (31.1) \|  \| \|  \| Serplulimab \| 0 (0.0) \| 10 (22.2) \|  \| \|  \| Durvalumab \| 0 (0.0) \| 8 (17.8) \|  \| \|  \| Atezolizumab \| 0 (0.0) \| 7 (15.6) \|  \| \|  \| Adebrelimab \| 0 (0.0) \| 6 (13.3) \|  \| \|  \| Sintilimab \| 43 (100.0) \| 0 (0.0) \|  \| \| **mPFS (95%CI)** \|  \| 6.9 (6.1-7.7) \| 6.0 (5.0-7.0) \| 0.066 \|   Abbreviations: IQR, Interquartile Range; ECOG, Eastern Cooperative Oncology Group; AJCC, American Joint Committee on Cancer; LDH, lactate dehydrogenase; ULN, upper limit of normal; PFS, Progression Free Survival |
| --- | --- | --- | --- | --- | --- | --- | --- | --- | --- | --- | --- | --- | --- | --- | --- | --- | --- | --- | --- | --- | --- | --- | --- | --- | --- | --- | --- | --- | --- | --- | --- | --- | --- | --- | --- | --- | --- | --- | --- | --- | --- | --- | --- | --- | --- | --- | --- | --- | --- | --- | --- | --- | --- | --- | --- | --- | --- | --- | --- | --- | --- | --- | --- | --- | --- | --- | --- | --- | --- | --- | --- | --- | --- | --- | --- | --- | --- | --- | --- | --- | --- | --- | --- | --- | --- | --- | --- | --- | --- | --- | --- | --- | --- | --- | --- | --- | --- | --- | --- | --- | --- | --- | --- | --- | --- | --- | --- | --- | --- | --- | --- | --- | --- | --- | --- | --- | --- | --- | --- | --- | --- | --- | --- | --- | --- | --- | --- | --- | --- | --- | --- | --- | --- | --- | --- | --- | --- | --- | --- | --- | --- | --- | --- | --- | --- | --- | --- | --- | --- | --- | --- | --- | --- | --- | --- | --- | --- | --- | --- | --- | --- | --- | --- | --- | --- | --- | --- | --- | --- | --- | --- | --- | --- | --- | --- | --- | --- | --- | --- | --- | --- | --- | --- | --- | --- | --- | --- | --- | --- | --- | --- | --- | --- | --- | --- | --- | --- | --- | --- | --- | --- | --- | --- | --- | --- |

**Supplementary Table 3. Multiplex immunofluorescence**

| Antibody | Clone | Dilution | Catlog |
| --- | --- | --- | --- |
| CD20 | EP459Y | 1:1000 | Ab78237 |
| CD200 | EPR22412-229 | 1:1000 | Ab254193 |
| CXCR5 | EPR23463-30 | 1:5000 | Ab254415 |
| CD4 | EPR6855 | 1:1000 | Ab133616 |
| CD8 | EPR22483-288 | 1:1000 | Ab245118 |
| CD103 | EPR22590-27 | 1:1000 | Ab224202 |
| IRF4 | EPR28687-87 | 1:200 | Ab315394 |
| CD68 | EPR20545 | 1:8000 | Ab213363 |
| CD163 | EPR19518 | 1:500 | Ab182422 |
| CSF-1R | SP211 | 1:200 | Ab183316 |
| SIGLEC5 | EPR26250-156 | 1:2000 | Ab307434 |

**Supplementary Table 4. Full-spectrum Flow Cytometry Panel**

| Fluorophore | Antibody | Manufacturers | Code | Clone |
| --- | --- | --- | --- | --- |
| PerCP | CD45 | Yuanqi Bio | W042-RB001-1 | HI30 |
| APC-Cy7 | CD3 | Yuanqi Bio | W031-RR001-2 | SK7 |
| RB515 | CD4 | Yuanqi Bio | W019-RB515-1 | SK3 |
| RB740 | CD8 | Yuanqi Bio | W032-RB740-1 | SK1 |
| RV580 | CD45RA | Yuanqi Bio | W027-RV580-1 | HI100 |
| PE-CY7 | CD62L | Yuanqi Bio | W625-RB006-1 | DREG-56 |
| RB594 | CD25 | Yuanqi Bio | W039-RB594-1 | M-A251 |
| PE-CY5 | CD127 | Yuanqi Bio | W049-RB003-1 | A019D5 |
| RV480 | CD20 | Yuanqi Bio | W703-RV480-1 | 2H7 |
| RV750 | CD56 | Yuanqi Bio | W043-RV750-1 | 5.1H11 |
| RV498 | CD16 | Yuanqi Bio | W037-RV498-1 | 3G8 |
| RV710 | HLA-DR | Yuanqi Bio | W026-RV710-1 | L243 |
| Alexa Fluor 647 | CLEC9A(CD370) | BD Bioscience | 564267 | 3A4/Clec9A |
| APC | CD83 | eBioscience | 17-0839-42 | HB15e |
| BV650 | CD1c | Biolegend | 331542 | L161 |
| RV450 | CD14 | Yuanqi Bio | W036-RV450-1 | M5E2 |
| BV510 | CD86 | Biolegend | 305432 | IT2.2 |
| BV605 | CD163 | Biolegend | 333615 | GHI/61 |
| FVS700 | LIVE/DEAD | BD Bioscience | 564997 | - |
