# Supplementary material for: Harnessing local and system immune profiling delineating differential responders to first-line sintilimab (anti-PD-1 antibody) combined with chemotherapy in extensive-stage small cell lung cancer: an exploratory biomarker analysis of a phase II study
Source: Signal Transduct Target Ther. 2025 May 23;10:168. doi: 10.1038/s41392-025-02252-5 (PMC12098833; doi:10.1038/s41392-025-02252-5)
Supplement: Supplementary file 2 — Study Protocol [file 41392_2025_2252_MOESM2_ESM.pdf]

A Phase II Clinical Study of First-Line Immunotherapy Combined with Chemotherapy for Advanced Small Cell Lung Cancer

Study Protocol

Protocol Number:
Version Number: 1.1
Date of Document: January 7, 2021

Clinical Trial Sponsoring Organization: Shanghai Pulmonary Hospital
Clinical Trial Data Statistics Organization:
Clinical Trial Applicant Organization: Shanghai Pulmonary Hospital

Protocol Summary

**Study Title**	A Phase II Clinical Study of First-Line Immunotherapy Combined with Chemotherapy for Advanced Small Cell Lung Cancer
**Study Number**	
**Version Number**	1.1
**Responsible Organization**	Shanghai Pulmonary Hospital
**Principal Investigator**	Chunxia Su
**Study Subjects**	Patients with Advanced (Extensive-Stage) Small Cell Lung Cancer
**Purpose of the Study**	Main Objective:<br>• To evaluate the 1-year Progression-Free Survival (PFS) rate of subjects receiving first-line treatment with PD-1 inhibitor combined with chemotherapy.<br>Secondary Objectives:<br>• To assess Progression-Free Survival (PFS), Overall Survival (OS), and Objective Response Rate (ORR) of subjects according to RECIST 1.1;<br>• To evaluate the quality of life of subjects based on the EORTC QLQ-C30 scoring;<br>• To assess the incidence of adverse events (AEs) in subjects.<br>Exploratory Objectives:<br>• To identify potential predictive biomarkers for the efficacy of immunotherapy in the treatment of advanced (extensive-stage) small cell lung cancer.
**Study Design**	This study is a single-center, prospective, single-arm, open-label Phase II clinical study to observe the efficacy and safety of Sintilimab combined with chemotherapy in previously untreated extensive-stage small cell lung cancer (SCLC) subjects. The trial plans to enroll 45 patients.

	Previously untreated extensive-stage SCLC patients, after signing the informed consent, will be screened and included if they meet the eligibility criteria. They will receive Sintilimab combined with chemotherapy (Carboplatin/Cisplatin + Etoposide) for 4 to 6 cycles, followed by maintenance therapy with Sintilimab until disease progression, death, intolerable toxicity, withdrawal of informed consent, initiation of new antitumor treatment, or other reasons for treatment termination as stipulated in the protocol. The early phase of this study is a safety lead-in period, observing the safety of 3 to 6 patients receiving first-line treatment with Sintilimab combined with chemotherapy (Carboplatin/Cisplatin + Etoposide): initially enrolling 3 patients and observing dose-limiting toxicities (DLTs) within the first cycle of the first medication, assessing the number of events, and if not more than one, the protocol will continue; otherwise, another 3 cases will be observed. During the safety lead-in period, if there are more than 4 DLT events, the dosage of the combined medication will be adjusted, and the study will proceed according to the adjusted dosage. The maximum duration of Sintilimab use is 2 years. After resistance to immunotherapy, the investigator will determine different treatment plans based on different resistance patterns, such as local therapy + immunotherapy.<br><br>The study uses RECIST v1.1 for clinical tumor imaging evaluation, which is performed every 6 weeks (±7 days) according to the patient's clinical condition; after 24 weeks of medication, it can be once every 9 weeks (±7 days) until disease progression, initiation of new antitumor treatment, withdrawal of the Informed Consent Form (ICF) by the subject, or death, or up to 2 years of Sintilimab use. Subjects who experience disease progression for the first time, if their clinical condition is stable and the investigator determines that they can continue to receive medication in this study, will do so. Patients who discontinue treatment for reasons other than disease progression still need to undergo imaging evaluation after stopping treatment according to the protocol until the next event occurs (initiation of new antitumor treatment, disease progression, withdrawal of ICF by the subject, or death).<br><br>After the study treatment is terminated, subjects will undergo safety follow-up (30±3 days after the last medication) and survival follow-up (every 90±7 days).<br><br>The primary endpoint of this study is the 1-year PFS rate, defined as the proportion of patients without disease progression at one year.
**Sample Size**	Based on previous research data, the 1-year PFS rate for patients with extensive-stage SCLC receiving first-line chemotherapy does not

**Estimation**	exceed 5.4%. It is estimated that the use of first-line Sintilimab combined with chemotherapy can increase the 1-year PFS rate of patients to 15%. Through sample size calculation, with α=0.05 (one-sided test), Power=80%, and assuming a dropout rate of 10%, using the One-Sample Logrank Test, a total of 45 patients are required to be enrolled.
**Inclusion Criteria**	(1) Age: ≥18 years and ≤75 years old;<br><br>(2) A confirmed diagnosis of extensive-stage small cell lung cancer (SCLC) by pathological examination (including histology or cytology);<br><br>(3) Previously untreated patients who have not received any systemic antitumor therapy;<br><br>(4) Asymptomatic patients with brain metastases, or those with brain metastases that have been treated and maintained a clinically stable condition for at least one month, and have not used corticosteroids and anticonvulsant medications for at least one month prior to entering the study;<br><br>(5) Measurable lesions (as defined by RECIST 1.1, the long diameter of the tumor lesion on CT scan is ≥10mm, the short diameter of the lymph node lesion on CT scan is ≥15mm, the scan slice thickness is no more than 5mm, and the measurable lesions have not received local treatments such as radiotherapy or cryotherapy);<br><br>(6) ECOG PS: 0-2;<br><br>(7) Estimated life expectancy ≥3 months;<br><br>(8) Adequate hematological function, defined as an absolute neutrophil count of ≥1.5×10^9/L, a platelet count of ≥80×10^9/L, and hemoglobin ≥90g/L (without transfusion or correction with G-CSF or other hematopoietic growth factors within 7 days);<br><br>(9) Adequate liver function, defined as a total bilirubin level ≤1.5 times the upper limit of normal (ULN) and levels of aspartate aminotransferase (AST) and alanine aminotransferase (ALT) ≤2.5 times ULN for all patients;<br><br>(10) Adequate renal function, defined as a creatinine clearance rate ≥50ml/min (Cockcroft-Gault formula);<br><br>(11) Adequate coagulation function, defined as an international normalized ratio (INR) or prothrombin time (PT) ≤1.5 times ULN; if the subject is receiving anticoagulant therapy, as long as the INR/PT is within the range specified for the anticoagulant medication, it is acceptable;

	(12) For female subjects of childbearing age, a negative urine or serum pregnancy test should be presented within 3 days prior to the first administration of the study drug; if the result of the urine pregnancy test cannot be confirmed as negative, a blood pregnancy test is required;<br><br>(13) If there is a risk of conception, male and female patients must use effective contraception (i.e., a method with a failure rate of less than 1% per year) and continue to do so for at least 180 days after discontinuation of the trial treatment; Note: If abstinence is the subject's usual lifestyle and the preferred method of contraception, abstinence can be accepted as a contraceptive method;<br><br>(14) The subject voluntarily participates in this study, signs a written informed consent form before any trial-related procedures are implemented, and has good compliance and cooperation with follow-up.
**Exclusion Criteria**	(1) Currently participating in an interventional clinical study treatment, or having received treatment with other investigational drugs or devices within 4 weeks prior to the first dosing;<br><br>(2) Having previously received the following therapies: anti-PD-1, anti-PD-L1, or anti-PD-L2 agents, or drugs targeting another co-stimulatory or co-inhibitory T-cell receptor (e.g., CTLA4, OX-40, CD137);<br><br>(3) Having received traditional Chinese medicine with anticancer indications or drugs with immunomodulatory effects (such as thymosin, interferons, interleukins, etc.) within 2 weeks prior to the first dosing, or having undergone significant surgical treatment within 3 weeks prior to the first dosing;<br><br>(4) Presence of clinically significant active hemoptysis, active diverticulitis, abdominal abscess, gastrointestinal obstruction, and peritoneal metastasis requiring clinical intervention;<br><br>(5) Having undergone organ or hematopoietic system transplantation;<br><br>(6) New York Heart Association class II-IV congestive heart failure, poorly controlled and clinically significant arrhythmias;<br><br>(7) Known hypersensitivity to the active ingredients of Sintilimab, Etoposide, Cisplatin, or Carboplatin, or any excipients;<br><br>(8) A history of active autoimmune diseases requiring systemic treatment (e.g., the use of disease-modifying drugs, corticosteroids, or immunosuppressants) within 2 years prior to the first dosing. Replacement therapies (such as thyroid hormone, insulin, or

	physiological doses of corticosteroids for adrenal or pituitary insufficiency) are not considered systemic treatment;<br><br>(9) Patients requiring long-term systemic use of corticosteroids. Patients who require intermittent use of bronchodilators, inhaled corticosteroids for COPD, asthma, or local injection of corticosteroids can be enrolled;<br><br>(10) Failure to fully recover from toxicities and/or complications caused by any intervention (i.e., ≤Grade 1 or returned to baseline, excluding fatigue or alopecia);<br><br>(11) A diagnosis of other malignant tumors within 5 years prior to the first dosing, excluding those with radical treatment of skin basal cell carcinoma, skin squamous cell carcinoma, and/or in situ carcinoma that has been radically excised. If other malignant tumors or lung cancer were diagnosed more than 5 years before dosing, pathological or cytological diagnosis of recurrent or metastatic lesions is required;<br><br>(12) A history of non-infectious pneumonia requiring corticosteroid treatment within 1 year prior to the first dosing or the presence of non-infectious pneumonia;<br><br>(13) Active infection requiring treatment or the use of systemic anti-infective drugs within one week prior to the first dosing;<br><br>(14) Known mental illness or drug abuse that may affect compliance with trial requirements;<br><br>(15) Known history of human immunodeficiency virus (HIV) infection (i.e., positive for HIV 1/2 antibodies), known syphilis infection (positive for syphilis antibodies), active pulmonary tuberculosis;<br><br>(16) Untreated active hepatitis B; Note: Hepatitis B subjects who meet the following criteria are also eligible for inclusion: The HBV viral load must be <1000 copies/ml (200 IU/ml) or below the lower limit of detection prior to the first dosing, and subjects should be closely monitored for viral reactivation throughout the entire study. During chemotherapy treatment, subjects receive anti-HBV treatment to prevent viral reactivation. For subjects who are anti-HBc (+), HBsAg (-), anti-HBs (-), and have an undetectable HBV viral load (-), prophylactic anti-HBV treatment is not required, but close monitoring for viral reactivation is necessary.<br><br>(17) Subjects with active HCV infection (positive for HCV antibodies and HCV-RNA levels above the lower limit of detection);<br><br>(18) Have received live vaccinations within 30 days prior to the first dosing; Note: Intramuscular inactivated viral vaccines for seasonal

	influenza are permitted; however, live attenuated influenza vaccines administered nasally are not allowed;<br><br>(19) Have a medical history, disease, treatment, or laboratory abnormalities that may interfere with the trial results, prevent full participation in the study, or that the investigator deems participation in the study is not in the best interest of the subject;<br><br>(20) Have local or systemic diseases caused by non-malignant tumors, or secondary reactions to cancer that could lead to increased medical risks and/or uncertainty in the evaluation of life expectancy.<br><br>(21) The investigator deems the subject unsuitable for inclusion.
**Study Medication and Administration Method**	1. Sintilimab: 100 mg/10 ml, 200 mg, administered via intravenous infusion on Day 1, Q3W (every 3 weeks)<br><br>2. Carboplatin: AUC 5 mg/ml/min, administered via intravenous infusion on Day 1, Q3W<br><br>3. Cisplatin: 20 mg/vial, 75 mg/m^2, administered via intravenous infusion on Day 1, Q3W<br><br>4. Etoposide: 100 mg/5 ml, 100 mg/m^2, administered via intravenous infusion on Days 1-3, Q3W<br><br>Continue until disease progression, death, intolerable toxicity, withdrawal of informed consent, initiation of new antitumor treatment, or termination for other reasons specified in the protocol, with a maximum duration of Sintilimab use of 2 years.
**Evaluation Criteria**	Therapeutic Efficacy Assessment:<br><br>- The investigator will assess therapeutic efficacy according to RECIST 1.1. The evaluation includes progression-free survival (PFS), 1-year PFS rate, overall survival (OS), and objective response rate (ORR).<br><br>Safety Assessment:<br><br>- The incidence, relationship to the study medication, and severity of all adverse events (AEs), treatment-related adverse events (TRAEs), immune-related adverse events (irAEs), and serious adverse events (SAEs);<br><br>- The number of subjects who discontinue the study due to the aforementioned adverse events;<br><br>- Changes in vital signs, physical examination results, and laboratory results before, during, and after the study treatment.

**Statistical Methods**	The statistical analysis primarily employs programming calculations using the SPSS statistical analysis software. Statistical tests utilize one-tailed and two-tailed tests, with a P-value less than or equal to 0.05 considered to indicate a statistically significant difference, and the confidence interval adopts a 95% confidence level.<br><br>Progression-free survival (PFS) and overall survival (OS) are estimated using the Kaplan-Meier method to determine the median values and their 95% CI, and survival curves are plotted. The objective response rate (ORR, which equals complete response [CR] plus partial response [PR]) and its 95% CI are calculated. Quality of life scores are compared with baseline examination values, and paired t-tests or signed rank tests are used to compare within-group differences before and after.<br><br>Safety evaluations are primarily based on descriptive statistical analysis, listing the incidence and severity of adverse events in this trial, and describing abnormal changes in laboratory test indicators, vital signs, and physical examination findings.

Visitation Flowchart

Table 1: Study Visit Process Chart

Phase	Screening Phase	Treatment Phase (Every 21 days as one treatment cycle)			Maintenance Treatment Phase (Every 21 days as one treatment cycle)	Safety Visit [15]	Survival Follow-up Visit [16]
		C1/D1	C2/D1	Cn/D1			
Day	-28~-1	1	22	21（n-1）+1		30 days post-final drug administration	Every 90 days
Time Window (Days)	NA	+3	±3	±3	±3	±7	±7
**General Study Process**							
Informed Consent [1]	X						
Inclusion/Exclusion Criteria	X						
Demographic/Medical History/History of Previous Lung Cancer Treatment [2]	X						
Concomitant Medications	X	X	X	X	X	X	
Vital Signs [3]	X	X	X	X	X	X	
Body Weight/Height [4]	X	X	X	X	X	X	
Physical Examination	X	X	X	X	X	X	
12-Lead Electrocardiogram (ECG) [5]	X		X	X	X	X	
Echocardiogram (Echo)	X						
Cardiac Enzyme Profile	X		X		X		
Survival Status							X
**Laboratory Assessment**							

Phase	Screening Phase	Treatment Phase (Every 21 days as one treatment cycle)			Maintenance Treatment Phase (Every 21 days as one treatment cycle)	Safety Visit [15]	Survival Follow-up Visit [16]
		C1/D1	C2/D1	Cn/D1			
Day	-28~-1	1	22	21（n-1）+1		30 days post-final drug administration	Every 90 days
Time Window (Days)	NA	+3	±3	±3	±3	±7	±7
Complete Blood Count (CBC) [6]	X		X	X	X	X	
Blood Biochemical Tests[6]	X		X	X	X	X	
Urine Routine Examination [6]	X		X	X	X	X	
Pregnancy Test [7]	X						
Thyroid Function [8]	X		X	X	X	X	
Viral Antibody Testing (HIV, HBV, and HCV)[9]	X						
**Safety Monitoring and Survival**							
ECOG PS score							
Adverse Event Assessment [10]	X	X	X	X	X	X	X
Subsequent Antitumor Therapy							X
Quality of Life Questionnaire							X
**Therapeutic Efficacy Assessment**							
Tumor Imaging Assessment [14]	X			X	X	X	
**Drug Administration** [15]							
Sintilimab		X	X	X	X		

Phase	Screening Phase	Treatment Phase (Every 21 days as one treatment cycle)			Maintenance Treatment Phase (Every 21 days as one treatment cycle)	Safety Visit [15]	Survival Follow-up Visit [16]
		C1/D1	C2/D1	Cn/D1			
Day	-28~-1	1	22	21（n-1）+1		30 days post-final drug administration	Every 90 days
Time Window (Days)	NA	+3	±3	±3	±3	±7	±7
Cisplatin/Carboplatin plus Etoposide		X	X	X			
**Biomarker**							
Archived/Fresh Tumor Tissue [13]	X						
Blood [14]		X		X			

Notes:

1. The signing of the Informed Consent Form (ICF) should be conducted before any operations stipulated by the protocol.
2. History of prior lung cancer treatment: All treatments for lung cancer, including chemotherapy, radiation therapy, and surgical treatment.
3. Vital signs include: body temperature, pulse, respiratory rate, and blood pressure.
4. Height measurement is only conducted during the screening phase. The weight of the subject is measured before each dosing. If the subject's weight fluctuates by less than 10% compared to the baseline weight (the day of the first dose of study treatment), the baseline weight is used to calculate the dosage of the chemotherapy drug. Otherwise, the actual dosage is calculated based on the weight on the day of the scheduled dosing.
5. The 12-lead electrocardiogram (ECG) is conducted during the screening phase, treatment phase, and safety follow-up.
6. The complete blood count includes: red blood cell count (RBC), hemoglobin (HGB), hematocrit (HCT), white blood cell count (WBC), platelet count (PLT), and white blood cell differential [lymphocytes, neutrophils, monocytes, eosinophils, basophils]. Blood biochemistry includes: liver function [Total Bilirubin (TBIL), Direct Bilirubin (DBIL), Alanine Aminotransferase (ALT), Aspartate Aminotransferase (AST), Gamma-Glutamyl Transferase (γ-GT), Alkaline Phosphatase (ALP), Albumin (ALB), Total Protein (TP), Lactate Dehydrogenase (LDH), Creatine Kinase (CK)], kidney function [Urea or Blood Urea Nitrogen (BUN), Creatinine (Cr)], blood electrolytes (Na, K, Cl, Mg, Ca, P), amylase, and fasting blood glucose (FBG). Urine routine examination includes: pH, Urinalysis White Blood Cells (UWBC), Urine Protein (UPRO), Urinalysis Red Blood Cells (URBC), Glucose (UGLU), and specific gravity. Subjects with urine routine showing protein ≥2+ during the

screening phase should undergo a 24-hour urine protein quantification. Conducted within 7 days before the first dosing of the study drug in the screening phase, within 3 days before the dosing of the study drug at the beginning of the second cycle, and during the safety follow-up.
7. Women of childbearing age will undergo urine or serum pregnancy tests within 3 days before the first dosing in the screening phase and during the safety follow-up. If the urine pregnancy test result cannot be confirmed as negative, a serum pregnancy test will be conducted, and the serum pregnancy test result will prevail.
8. Thyroid function tests are conducted within 28 days before the first dosing, within 3 days before the dosing of the study drug at the beginning of the second cycle, and during the safety follow-up. In the screening phase, T3/FT3, FT4, and TSH are checked; from the second cycle, only TSH is checked, and if abnormal, other thyroid function indicators may be considered. During the study treatment process, it is not necessary to wait for the results of this test before proceeding with the study drug administration.
9. Include HIV and HCV antibody tests, as well as hepatitis B panel tests (HBsAg, HBsAb, HBcAb, HBeAg, HBeAb), which must be completed within 28 days before the first dosing.
10. AE and laboratory test safety assessments will be conducted according to CTCAE v5.0. The definition, recording, relatedness judgment, severity judgment, reporting timelines, and management of AEs and SAEs refer to the description in Section 8 of the protocol.
11. The quality of life questionnaire includes EORTC QLQ-C30 (V3.0 Chinese version), which is assessed on the day of the first dosing (before dosing), at each imaging assessment, and during safety follow-up. If an unplanned imaging assessment is conducted, the quality of life questionnaire should also be administered concurrently.
12. Tumor assessment includes RECIST 1.1 assessment. It is assessed by the investigator and must include the cervical, thoracic, abdominal, and pelvic regions. According to the RECIST v1.1 criteria, baseline (within 28 days before the first dosing), an assessment is conducted every 6 weeks (±7 days); after 24 weeks of medication, an assessment can be done every 9 weeks (±7 days) until disease progression, initiation of new antitumor treatment, withdrawal of ICF by the subject, or death. Subjects who experience disease progression for the first time, if their clinical condition is stable, the investigator will determine whether they can continue treatment.
13. Subjects are required to provide archived or fresh tumor tissue samples during the screening phase.
14. Subjects are required to provide a 10 ml whole blood sample for the detection of tumor biological markers at the following time points: before the first dosing, during the treatment phase before the next treatment and imaging assessment, and when disease progression is confirmed.
15. Safety follow-up will be conducted 30±3 days after the last dosing or before the initiation of new antitumor treatment, whichever occurs first. All AEs occurring before the safety follow-up visit should be recorded until they are resolved to grade 0-1 or baseline levels, or the investigator deems further follow-up is unnecessary for justified reasons (whichever occurs first). SAEs occurring within 90 days after the last dosing or before the subject starts new anticancer treatment (whichever occurs first) should be followed up and recorded.

16. Survival follow-up: Conducted every 90 days (±7 days) after the safety visit, which can be done via telephone.

Contents

1 RESEARCH BACKGROUND 18

1.1 DISEASE BACKGROUND 18

1.2 RISK AND BENEFIT ASSESSMENT 22

2 RESEARCH PURPOSE 22

2.1 MAIN PURPOSE 22

2.2 SECONDARY PURPOSES 22

2.3 EXPLORATORY PURPOSES 22

3 STUDY DESIGN 22

3.1 OVERALL DESIGN 22

3.1.1 Sample Size Estimation 23

4 STUDY POPULATION 23

4.1 INCLUSION CRITERIA 23

4.2 EXCLUSION CRITERIA 25

4.3 RESTRICTIONS DURING THE STUDY PERIOD 26

4.3.1 Pregnancy 26

4.3.2 Fertility 26

4.3.3 Lactation 27

4.4 CRITERIA FOR SUBJECT DISCONTINUATION OF TREATMENT/WITHDRAWAL FROM THE STUDY 28

4.4.1 Discontinuation of Study Treatment 28

4.4.2 Withdrawal from the Study 28

4.4.3 Clinical Criteria for Early Termination of the Study 29

5 STUDY MEDICATION AND OTHER TREATMENTS 29

5.1 USE OF STUDY MEDICATION 29

5.1.1 Administration of Sintilimab 29

5.1.2 Use of Chemotherapy Medications 30

5.2 DOSE ADJUSTMENTS 32

5.2.1 Dose Adjustments for Sintilimab 32

5.2.2 Management of Sintilimab-Related Infusion Reactions 34

5.2.3 Dose Adjustment of Chemotherapy Drugs: 37

5.3 PRINCIPLES FOR MANAGING TOXICITY OF IMMUNE CHECKPOINT INHIBITORS 39

5.4 Concurrent Therapies 39
5.4.1 Permissible Concurrent Therapies *39*
5.4.2 Prohibited Concurrent Therapies *39*
5.5 Drug Administration 41
5.5.1 Drug Management *41*
5.5.2 Disposal and Destruction of Study Medication *41*
5.6 Documentation of Study Medication 41
5.7 Complaint Handling 41
6 STUDY PROCESS 42
6.1 Subject Screening 42
6.1.1 Subject Screening *42*
6.2 Study Plan and Scheduling 42
6.2.1 Screening Period *42*
6.2.2 Treatment/Maintenance Treatment Visits *43*
6.2.3 Safety Follow-up Visit *44*
6.2.4 Survival Visits *44*
6.2.5 Subsequent Anticancer Treatment Status *45*
6.3 Other Procedures 45
6.3.1 Discontinuation of Treatment/Withdrawal from the Study *45*
6.3.2 Loss to Follow-up *45*
7 STUDY EVALUATIONS 46
7.1 Efficacy Assessment 46
7.1.1 Baseline Tumor Imaging Assessment *46*
7.1.2 Tumor Imaging Assessment During the Study *46*
7.1.3 Tumor Imaging Examinations at the End of Treatment and Follow-up Period 47
7.2 Safety Assessment 47
7.2.1 Physical Examination: *47*
7.2.2 Laboratory Test Assessments: *48*
7.3 Quality of Life Assessment *49*
7.4 Storage and Destruction of Biological Samples *49*
8 SAFETY REPORTING AND ADVERSE EVENT MANAGEMENT 49
8.1 Definition of Adverse Events 49
8.2 Criteria for Assessing the Severity of Adverse Events 49
8.3 Criteria for Assessing the Relationship between Adverse Events and the

INVESTIGATIONAL MEDICATION 50
8.4 SERIOUS ADVERSE EVENTS (SAEs) 52
8.4.1 Pregnancy 52
8.4.2 Disease Progression 53
8.4.3 Undertaking Other Anticancer Treatments 53
8.4.4 Hospitalization 53
8.4.5 Reporting System for Serious Adverse Events 54
8.5 RAPID REPORTING OF SAEs AND PREGNANCY 55
8.6 EVENTS OF ABNORMAL LIVER FUNCTION 56
8.7 MANAGEMENT OF DRUG-RELATED TOXICITY 57
8.7.1 Immune-Related Adverse Events 57
9 STATISTICAL ANALYSIS 57
9.1 SAMPLE SIZE CALCULATION 57
9.2 STATISTICAL ANALYSIS PLAN 58
10 QUALITY ASSURANCE AND QUALITY CONTROL 59
11 ETHICAL, REGULATORY, AND ADMINISTRATIVE PRINCIPLES 59
11.1 ETHICAL PRINCIPLES 59
11.2 LEGAL AND REGULATORY COMPLIANCE 59
11.3 INFORMED CONSENT 60
11.4 DATA PROTECTION 60
11.5 CONFIDENTIALITY AGREEMENT 60
11.6 RECORD KEEPING 60
11.7 EARLY TERMINATION OF THE STUDY 61
11.8 INSPECTIONS BY THE RESPONSIBLE RESEARCH ORGANIZATION AND REGULATORY AUTHORITIES 61
12 STUDY MANAGEMENT 61
12.1 ACCESS TO ORIGINAL DATA/DOCUMENTS 61
12.2 PROTOCOL AMENDMENTS 61
12.3 PUBLICATION 62
13 REFERENCE 62
14 APPENDIX 65
APPENDIX 1: PERFORMANCE STATUS SCORING CRITERIA (ECOG PS) 65

Appendix 2: Formulas for Calculating Creatinine Clearance and Body Surface Area 65
Appendix 3: Response Evaluation Criteria in Solid Tumors (RECIST) Version 1.1 (Excerpt) 66

1 Research Background

1.1 Disease Background

Lung cancer is the most common malignant tumor in our country. Data released by the National Cancer Registration Center in 2016 show that the incidence of lung cancer ranks first among male patients and is second only to breast cancer among female patients [1]. Non-small cell lung cancer (NSCLC) accounts for about 85%, while small cell lung cancer (SCLC) accounts for about 15% of lung cancers. It is a highly invasive neuroendocrine tumor. Early diagnosis of SCLC is difficult, and it is usually widely staged (extensive-stage disease, ESSCLC, where the tumor and its invasion range exceed a radiation therapy field) [2, 3] upon diagnosis. In recent years, immunotherapy drugs, especially PD1/PDL1 inhibitors, have brought about a tremendous transformation in the treatment of malignant tumors, changing the treatment patterns of various tumors including NSCLC. Exploration has also been conducted in the field of SCLC with different drugs, different treatment strategies (monotherapy or combination), different treatment durations (subsequent or first-line), and different treatment populations (unselected or selected).

As early as 2016, the Checkmate032 study [4] showed that the combination of Nivolumab and Ipilimumab had good survival benefits in the treatment of relapsed SCLC, and was recommended by NCCN as a 2A class evidence for the treatment of SCLC relapsed within 6 months in 2017. In 2018, the Checkmate032 study published the subgroup analysis results of Nivolumab monotherapy in the third-line treatment population in the JTO magazine, with a median duration of response (DoR) of 17.9 months, a median PFS of 1.4 months, a 6-month PFS of 17.2%, a median OS of 5.6 months, a 12-month OS rate of 28.3%, and an 18-month OS rate of 20.0% [5]. Based on these results, the FDA approved nivolumab in August 2017 for the treatment of ESSCLC that has been treated with platinum-based chemotherapy and at least one other therapy. This was the first SCLC third-line treatment plan approved in the history of the FDA, breaking the silence of new drug treatment for SCLC for 20 years.

At the 2018 ASCO Annual Meeting, the KEYNOTE-158 study [6] on Pembrolizumab for the treatment of advanced SCLC was announced, confirming that Pembrolizumab has good efficacy and safety in the treatment of relapsed SCLC. Based on this study, NCCN also made a 2A class recommendation for Pembrolizumab in the first edition of the 2019 NCCN guidelines, further enriching the treatment options for relapsed SCLC. In addition, the results of the KEYNOTE028 study [7] were announced at the 2015 ASCO, which mainly evaluated the PD-L1 positive ESSCLC patients who failed the initial chemotherapy and were given Pembrolizumab monotherapy. The results showed that among the 20 treated

patients, 7 patients achieved partial response (PR), with an ORR of 35% and a median duration of response of 8.6 weeks, indicating that it has good efficacy in the treatment of ESSCLC. The combined summary of the KEYNOTE-158 phase II clinical trial and the KEYNOTE-028 phase Ib clinical trial showed that the ORR of Pembrolizumab reached 19.3%, with 2 patients achieving complete response and 14 patients achieving partial response; among the patients who achieved response, 65% of the patients had a response duration of more than 18 months [8]. Based on this result, in June 2019, the US FDA accelerated the approval of Pembrolizumab for the treatment of advanced SCLC patients who have received platinum-based chemotherapy and at least one other previous therapy.

After successfully entering the later-line treatment, immunotherapy has also made significant breakthroughs in the first-line treatment of SCLC. On March 18, 2019, Atezolizumab + Etoposide + Carboplatin was approved by the FDA for the first-line treatment of ED-SCLC. In the IMpower133 study [9], which served as the basis for approval, Atezolizumab + Etoposide + Carboplatin extended the median OS (12.3 vs 10.3 months) and median PFS (5.2 vs 4.3 months) compared to placebo + Etoposide + Carboplatin. In addition, the data released from the CASPIAN study also showed the advantages of immunotherapy in improving the survival of patients, with the OS of patients combined with Durvalumab being significantly extended: the median OS was 13.0 months and 10.3 months (HR=0.73, 95% CI 0.59-0.9, P = 0.0047). These studies show that the prospects for the treatment of extensive or relapsed metastatic SCLC with immune checkpoint inhibitors are broad.

Immunotherapy has brought new breakthroughs to the treatment of SCLC, but there are still many issues that need to be further clarified. First, at present, only inhibitors targeting PD-L1 have shown effectiveness in the first-line treatment of SCLC, and it is not clear whether this is due to the differences in the drugs themselves or the different mechanisms of action of PD-1 and PD-L1 inhibitors. It is unknown whether our country's independent research and development of PD-1/PD-L1 inhibitors, such as Sintilimab, can also achieve the same efficacy? Second, there are currently no immune biomarkers to guide the immunotherapy of SCLC, and it is impossible to accurately distinguish patients who benefit and those who do not.

The expression level of PD-L1 in tumor tissue is the first accompanying diagnostic molecular marker approved by the FDA for Pembrolizumab immunotherapy [10]. Gadgeel and others assessed the correlation between the expression level of PD-L1 in the tumor stroma and the efficacy in patients with advanced SCLC who received Pembrolizumab maintenance therapy after first-line chemotherapy. The results showed that patients with

positive PD-L1 expression in the tumor stroma had significantly longer median PFS and median OS compared to patients with negative PD-L1 expression (median PFS: 6.5 months vs 1.3 months; median OS: 12.8 months vs 7.6 months) [11]. The exploratory analysis results of KEYNOTE-158 also indicated that among the 107 enrolled patients, 39% had PD-L1 ≥ 1%, and the clinical disease control rate was significantly related to PD-L1 expression [6]. However, the CheckMate 032 study suggested that the expression level of tumor PD-L1 in SCLC patients is not related to the clinical benefit of immunotherapy: the ORR in the monotherapy group was 14% vs 9% for PD-L1 < 1% and PD-L1 ≥ 1%, and the combined treatment group was 32% vs 10%, that is, the ORR of patients with PD-L1 < 1% was higher than that of patients with PD-L1 ≥ 1% [12]. It can be seen that the expression level of PD-L1 is a potential biomarker for predicting the efficacy of SCLC, but more research is still needed to clarify this.

Tumor mutation burden (TMB) has been proven to be a biomarker related to the efficacy of immunotherapy in various cancers. In a retrospective analysis of the CheckMate032 study, TMB was assessed by detecting the total number of non-synonymous mutations in tumor tissue through whole exon sequencing (WES), and patients were divided into high TMB (>248), medium TMB (143-247), and low TMB (0-142) groups. The results suggested that patients in the high TMB group had better clinical benefits from the combination therapy of nivolumab and ipilimumab (1-year PFS rate: 30% vs 8% vs 6.2%; 1-year OS rate: 62.4% vs 19.6% vs 23.4%) [13]. However, the results of the IMpower133 study showed that TMB in tumor tissue could not predict the efficacy of Atezolizumab combined with chemotherapy in SCLC patients [9]. Therefore, although TMB is considered a potential biomarker for predicting the response of advanced SCLC patients to immunotherapy, it still has many limitations, such as low predictive efficacy (the efficacy rate of high TMB patients is less than 50%), large differences in detection platforms, non-unified threshold standards, long reporting cycles for test results, and insufficient supporting evidence.

The T-cell inflammation gene expression profile score (T-Cell-Inflamed Gene-Expression Profile, GEP) is a new biomarker related to the efficacy of immunotherapy. Ayers M and others first proposed it in a study of melanoma, where researchers found that the expression of T-cell inflammation-related genes composed of antigen presentation, chemokine expression, cytotoxicity, and adaptive immunity was related to the response to Pembrolizumab. It was further verified in 9 types of tumors such as head and neck cancer and gastric cancer, and finally determined to be composed of 18 T-cell inflammation-related genes [14]. Patrick A Ott and others further verified its predictive effect on longer PFS in

Pembrolizumab treatment in 20 types of tumors, including SCLC [15], but more data is still lacking.

Hardy-Werbin M and others found that the baseline serum cytokine levels (IL-2) in SCLC patients before immunotherapy and the changes in cytokine levels (IL-4) during treatment are related to the prognosis of immunotherapy [16]. They also found that the concentration of autoantibodies in the baseline serum is also related to the efficacy of SCLC immunotherapy [17]. Other predictive biomarkers for the efficacy of SCLC immunotherapy are currently less reported, but may be inspired by the mechanism of immunotherapy and the exploration of related biomarkers in NSCLC. For example, McGranahan N and others reported that the enrichment of cloned neoantigens can enhance the sensitivity of NSCLC to PD-1 and CTLA-4 inhibitors by stimulating the immune response of T cells [18]. The number and phenotype of tumor-infiltrating lymphocytes (TILs) in tumor tissue are also related to the prognosis of NSCLC immunotherapy [19]. The diversity of the T cell receptor (TCR) library of PD-1+CD8+ T cells can also serve as a prognostic marker for NSCLC immunotherapy [20]. It can be seen that the biological markers in this tumor immune ring, from the production of effective new antigens (cloned neoantigens), the infiltration of T cells (TILs), to the effector stage of T cells (TCR), can all affect the final efficacy of immune checkpoint inhibitors. Therefore, the combination of biological markers in this tumor immune ring can become a direction for exploring effective markers for immunotherapy.

The applicant previously screened a combination of 5 autoantibodies in NSCLC patients receiving immunotherapy based on PD-1/PD-L1 inhibitors, verified their baseline concentration's predictive value for the efficacy of immunotherapy, and found that the positivity of this group of autoantibodies also seems to be related to the benefits of SCLC immunotherapy. Based on the above background, this project plans to carry out a phase II study of advanced SCLC patients receiving first-line PD-1 inhibitor combined with chemotherapy, aiming to provide evidence-based medical evidence for the use of domestic PD-1 inhibitors combined with chemotherapy for the first-line treatment of advanced SCLC. At the same time, by detecting autoantibodies and other indicators such as TMB, TILs infiltration, PD-L1 expression in tumor tissue and/or blood samples before and after treatment of enrolled patients, explore potential immune-based treatment plans, and predict the efficacy of biological markers, in order to help clinical practice to screen out the real group of immune benefits, thereby improving the overall survival prognosis of SCLC patients, and providing a basis for future prospective clinical studies guided by markers.

1.2 Risk and Benefit Assessment

The clinical samples collected in this project mainly include tumor tissue and blood specimens from eligible subjects. The possible risks and benefits are as follows:

The collection of clinical specimens may have some very small risks, including brief pain, local bruising, a few people will have mild dizziness, or extremely rare needle infections. The collection of specimens is operated by professional clinical personnel in this project, strictly following the operation specifications to reduce the occurrence of the above risks. Through the analysis of the clinical specimens of the subjects, it is helpful to make a diagnosis of the disease and provide necessary recommendations for the treatment of the subjects.

2 Research Purpose

2.1 Main Purpose

According to the RECIST 1.1 standard, the 1-year PFS rate of immunotherapy combined with first-line treatment for advanced small cell lung cancer is evaluated by the researcher.

2.2 Secondary Purposes

- Evaluate the PFS, OS, and ORR of the subjects according to RECIST 1.1;
- Assess the quality of life of the subjects based on the EORTC QLQ-C30 score;
- Assess the incidence of adverse events (AE) in subjects.

2.3 Exploratory Purposes

- Search for potential predictive biomarkers of efficacy for immunotherapy in advanced (extensive stage) small cell lung cancer.

3 Study Design

3.1 Overall Design

This study is a single-center, prospective, single-arm, open-label Phase II clinical trial to observe the efficacy and safety of sintilimab combined with chemotherapy in subjects with untreated extensive-stage small cell lung cancer (SCLC). The study plans to enroll 45 patients with untreated extensive-stage SCLC. After signing the informed consent form and meeting the inclusion and exclusion criteria, they will receive sintilimab combined with chemotherapy (carboplatin/ cisplatin + etoposide) for 4 or 6 cycles, followed by maintenance therapy with sintilimab until disease progression, death, intolerable toxicity, withdrawal of informed consent, initiation of new antitumor treatment, or termination for other reasons specified in the protocol, with a maximum treatment duration of 2 years for sintilimab. After resistance to immunotherapy, the researcher will judge and provide

different treatment plans according to different resistance patterns, such as local treatment + immunotherapy.

During the study, if a subject experiences clinically unstable disease, intolerable toxicity, receives new antitumor treatment, withdraws informed consent, is lost to follow-up or dies, or other conditions judged by the researcher to stop treatment occur, the study treatment should be stopped, with the first occurrence being decisive. The maximum treatment time for sintilimab is 2 years.

After stopping the study treatment, patients should continue to undergo imaging assessments at the scheduled time points according to the protocol until tumor progression. After tumor progression, all patients should enter the survival follow-up period. If other conditions requiring cessation of medication occur during the treatment period (such as intolerable toxicity), the treatment ends, and the patient enters the post-treatment visit, followed by the survival follow-up period.

Researchers will monitor adverse events (AEs) throughout the trial process and grade the severity of AEs according to the guidelines of the Common Terminology Criteria for Adverse Events (CTCAE) version 5.0 by the National Cancer Institute (NCI). After the end of the treatment, a 30-day safety follow-up will be conducted for the subjects to monitor AEs. If the patient has not received new antitumor treatment within 90 days after the last medication, serious adverse events (SAEs) within 90 days after the last medication will be collected. If the subject has started new anticancer treatment, SAEs before the new anticancer treatment will be collected, with the first occurrence being decisive.

This study will be conducted in accordance with the Good Clinical Practice (GCP) guidelines.

3.1.1 Sample Size Estimation

According to previous research data, the 1-year PFS rate for patients with extensive-stage SCLC receiving first-line chemotherapy does not exceed 5.4%. We estimate that the use of first-line sintilimab combined with chemotherapy can increase the 1-year PFS rate of patients to 15%. Through sample size calculation, with $\alpha=0.05$ (one-sided test), Power=80%, assuming a dropout rate of 10%, and using the One-Sample Logrank Test, a total of 45 patients need to be enrolled.

4 Study Population

4.1 Inclusion Criteria

(1) Age: ≥18 years and ≤75 years old;

(2) A diagnosis of extensive-stage small cell lung cancer confirmed by pathology (including histology or cytology);

(3) Previously untreated patients who have not received any systemic antitumor therapy;

(4) Asymptomatic patients with brain metastases, or those with brain metastases that have been treated and have maintained a clinically stable condition for at least one month, and have not used steroids and anticonvulsant drugs for at least one month before entering the study;

(5) Measurable lesions (according to RECIST 1.1 criteria, the longest diameter of the tumor lesion on CT scan is ≥10mm, the shortest diameter of the lymph node lesion on CT scan is ≥15mm, the scan slice thickness is no more than 5mm, and the measurable lesions have not received local treatments such as radiotherapy or cryotherapy);

(6) ECOG PS: 0-2 points;

(7) Expected survival of ≥3 months;

(8) Adequate hematological function, defined as an absolute neutrophil count of ≥1.5×10^9/L, platelet count of ≥80×10^9/L, and hemoglobin ≥90g/L (no blood transfusion history within 7 days, and not corrected with G-CSF or other hematopoietic growth factors);

(9) Adequate liver function, defined as a total bilirubin level ≤1.5 times the upper limit of normal (ULN) and aspartate aminotransferase (AST) and alanine aminotransferase (ALT) levels ≤2.5 times ULN for all patients;

(10) Adequate renal function, defined as a creatinine clearance rate ≥50ml/min (Cockcroft-Gault formula);

(11) Adequate coagulation function, defined as an international normalized ratio (INR) or prothrombin time (PT) ≤1.5 times ULN; if the subject is undergoing anticoagulant therapy, as long as INR/PT is within the range specified for the anticoagulant medication;

(12) For female subjects of childbearing age, a negative urine or serum pregnancy test should be presented within 3 days before the first administration of the study drug. If the urine pregnancy test result cannot be confirmed as negative, a blood pregnancy test is required;

(13) If there is a risk of conception, male and female patients must use effective contraception (i.e., a method with a failure rate of less than 1% per year) and continue to do so for at least 180 days after stopping the trial treatment; Note: If abstinence is the subject's usual lifestyle and the preferred method of contraception, then abstinence can be accepted as a contraceptive method;

(14) The subject voluntarily participates in this study, signs a written informed consent form before any trial-related procedures are implemented, has good compliance, and cooperates with follow-up visits.

4.2 Exclusion Criteria

(1) Currently participating in interventional clinical research treatment, or having received other investigational drugs or investigational device treatments within 4 weeks before the first administration of the study drug;

(2) Having previously received the following therapies: anti-PD-1, anti-PD-L1, or anti-PD-L2 drugs or drugs targeting another stimulatory or co-inhibitory T-cell receptor (e.g., CTLA4, OX-40, CD137);

(3) Having received traditional Chinese medicine with antitumor indications or drugs with immunomodulatory effects (e.g., thymosin, interferons, interleukins, etc.) within 2 weeks before the first administration of the study drug, or having undergone major surgical treatment within 3 weeks before the first administration of the study drug;

(4) Presence of clinically significant active hemoptysis, active diverticulitis, abdominal abscess, gastrointestinal obstruction, and peritoneal metastasis requiring clinical intervention;

(5) Having undergone organ or hematopoietic system transplantation;

(6) II-IV level congestive heart failure (New York Heart Association classification), poorly controlled and clinically significant cardiac;

(7) Known allergic reactions to the active ingredient of sintilimab or any excipients;

(8) A history of active autoimmune diseases requiring systemic treatment (e.g., the use of disease-modifying drugs, corticosteroids, or immunosuppressants) within 2 years prior to the first administration. Replacement therapies (e.g., thyroid hormone, insulin, or physiological doses of corticosteroids for adrenal or pituitary insufficiency, etc.) are not considered systemic treatment;

(9) Patients requiring long-term systemic use of corticosteroids. Patients who intermittently use bronchodilators, inhaled corticosteroids, or receive local injections of corticosteroids due to COPD or asthma are eligible for inclusion;

(10) Inadequate recovery from any intervention-related toxicity and/or complications before starting treatment (i.e., ≤Grade 1 or baseline, excluding asthenia or alopecia);

(11) A diagnosis of other malignant tumors within 5 years prior to the first administration, excluding those with radical treatment of skin basal cell carcinoma, squamous cell carcinoma, and/or in situ carcinoma that has been radically excised. If other malignant tumors or lung cancer were diagnosed more than 5 years before administration, pathological or cytological diagnosis of recurrent or metastatic lesions is required;

(12) A history of non-infectious pneumonia requiring corticosteroid treatment within 1 year prior to the first administration, or the presence of non-infectious pneumonia;

(13) Active infections requiring treatment or the use of systemic anti-infective drugs within one week prior to the first administration;
(14) Known mental disorders or drug abuse that may affect compliance with trial requirements;
(15) Known history of human immunodeficiency virus (HIV) infection (i.e., positive for HIV 1/2 antibodies), known syphilis infection (positive for syphilis antibodies), active pulmonary tuberculosis;
(16) Untreated active hepatitis B. Note: Hepatitis B subjects meeting the following criteria are also eligible for inclusion: HBV viral load must be <1000 copies/ml (200 IU/ml) or below the lower limit of detection before the first administration, and subjects should receive anti-HBV treatment throughout the study chemotherapy treatment period to prevent viral reactivation. For subjects with positive anti-HBc, negative HBsAg, negative anti-HBs, and negative HBV viral load, no prophylactic anti-HBV treatment is required, but close monitoring for viral reactivation is necessary;
(17) Subjects with active HCV infection (positive for HCV antibodies and HCV-RNA levels above the lower limit of detection);
(18) Received live vaccines within 30 days before the first administration. Note: Injectable inactivated virus vaccines for seasonal influenza are allowed, but live attenuated influenza vaccines administered nasally are not permitted;
(19) Existence of medical history, diseases, treatments, or laboratory abnormalities that may interfere with the trial results, prevent full participation in the study, or the investigator deems participation not to be in the best interest of the subject;
(20) Local or systemic diseases caused by non-malignant tumors, or secondary reactions to cancer, which may lead to high medical risk and/or uncertainty in survival assessment.
(21) Subjects whom the investigator deems unsuitable for inclusion.

4.3 Restrictions during the study period

4.3.1 Pregnancy

It is known that human IgG1 and IgG4 can cross the placental barrier, so medication during pregnancy is not recommended. Women who are pregnant are not eligible for this study.

4.3.2 Fertility

Female subjects of childbearing age who have active sexual life with male partners who have not undergone sterilization surgery, and male subjects who have active sexual life with female partners of childbearing age and have not undergone sterilization, must use one of the acceptable effective contraceptive methods listed in Table 2 from the

screening period until 180 days after the last administration of the medication, and should discuss with a responsible physician about when to stop using contraceptive measures after that time point. Periodic abstinence, safe period contraception, and withdrawal are not acceptable contraceptive methods. A female of childbearing age is defined as a woman who has had menarche, has not undergone sterilization surgery (i.e., bilateral tubal ligation, bilateral salpingectomy, or total hysterectomy), and has not yet reached menopause.

Table 2: Effective Contraceptive Methods (At least one method must be used)

Barrier Methods	Intrauterine Device Methods	Hormonal Methods
**Male condoms with spermicide**	Copper T-shaped IUD	Implant
**Diaphragm with spermicide**	Progestin T-shaped IUD	Hormonal contraceptive injection or depot medroxyprogesterone acetate
**Cervical cap with spermicide**	Levonorgestrel-releasing intrauterine system (e.g., Mirena®)	Combined oral contraceptives
**Contraceptive sponge**		Low-dose oral contraceptive pills
		Contraceptive patch

a. This is also considered a hormonal method

A woman is considered to be in menopause if she has not menstruated for 12 months without an alternative medical reason. The age requirements are as follows:

- If a woman ≥50 years old has not menstruated for 12 months or more after stopping exogenous hormone therapy, and her levels of progesterone and follicle-stimulating hormone are within the recognized postmenopausal range, she may be considered a postmenopausal woman.
- If a woman <50 years old has not menstruated for 12 months or more after stopping all exogenous hormone therapy, if she has had radiation-induced oophorectomy and her last menstruation occurred more than 1 year ago, if chemotherapy-induced amenorrhea has lasted for more than 1 year since her last menstruation, or if she has undergone surgical sterilization (bilateral oophorectomy or hysterectomy), she may be considered a postmenopausal woman.

4.3.3 Lactation

It is unknown whether sintilimab is secreted in breast milk. Considering that many

drugs are present in breast milk, sintilimab may have potential toxicity to infants. Women who are breastfeeding are not eligible for this study.

4.4 Criteria for Subject Discontinuation of Treatment/Withdrawal from the Study

4.4.1 Discontinuation of Study Treatment

Discontinuation of study treatment does not mean withdrawal from the study. Since certain clinical event data after discontinuation of treatment may be very important for the study, this information must be collected up to the subject's last scheduled visit, even if the subject has already stopped treatment.

Subjects may discontinue treatment at any time for any reason, or the researcher may decide whether to discontinue their treatment when any adverse event occurs. In addition, if the subject is not suitable for treatment, violates the study protocol, or for management and/or other safety reasons, the researcher or sponsor may discontinue the subject's treatment.

For any of the following reasons, the subject must discontinue treatment but can continue to be monitored in the study:

- The subject or the subject's legal representative requests to discontinue treatment.
- An adverse event occurs that requires treatment to be stopped as stipulated in the protocol (refer to Section 5.2).
- Another malignant tumor requiring active treatment occurs.
- A concurrent disease occurs that prevents further treatment.
- The researcher decides to withdraw the subject from the study.
- The subject's serum pregnancy test result is positive.
- The subject has poor compliance.
- The researcher and/or the sponsor believes that continuing to administer the study drug will place the subject at unnecessary risk based on the subject's disease condition or personal situation.
- Completion of 2 years of sintilimab treatment.

For subjects who have discontinued treatment but continue to be visited in the study, all visits and procedures listed in the study flowchart should be completed.

4.4.2 Withdrawal from the Study

If the subject or the subject's legal representative withdraws the informed consent to participate in the study, the subject must withdraw from the study. If the subject withdraws from the study, they will no longer receive treatment or scheduled visits. With the subject's consent, the subject can receive survival follow-up after withdrawing from the study. If the subject is lost to follow-up, they must withdraw from the study.

4.4.3 Clinical Criteria for Early Termination of the Study

The study will be prematurely terminated if the following criteria are met:

- The quality and quantity of data recorded are inaccurate or incomplete.
- Compliance with the study protocol and regulatory requirements is poor.

The incidence or severity of adverse drug reactions in this study or other studies suggests potential harm to the health of subjects. If the sponsor decides to no longer provide the study drug, adequate notice will be given to allow for appropriate adjustments to the subject's treatment.

5 Study Medication and Other Treatments

For all other study treatment cycles, the medication may be administered within 3 days before or after the first day of the planned weekly cycle at the discretion of the investigator for management reasons.

5.1 Use of Study Medication

The study medications in this trial are Sintilimab, Etoposide, Carboplatin, or Cisplatin (see Table 3). Other medications used during the study are considered non-study medications. Treatment plan: Sintilimab in combination with chemotherapy (Carboplatin/Cisplatin + Etoposide) for 4 or 6 cycles, followed by maintenance therapy with Sintilimab until disease progression, death, intolerable toxicity, withdrawal of informed consent, initiation of new antineoplastic treatment, or termination for other reasons specified in the protocol (whichever occurs first); eligible subjects may continue to use Sintilimab after the first disease progression (see Section 5.1.2); after resistance to immunotherapy, the investigator will determine and provide different treatment plans according to different resistance patterns, such as local treatment + immunotherapy.

5.1.1 Administration of Sintilimab

Sintilimab's main active ingredient is a recombinant humanized anti-programmed cell death receptor-1 monoclonal antibody with a concentration of 10 mg/mL. The product is a clear, colorless liquid, free of foreign matter, flocculent matter, and precipitate. Excipients include 140 mmol/L Mannitol, 25 mmol/L Histidine, 20 mmol/L Sodium Citrate Dihydrate, 50 mmol/L Sodium Chloride, 0.02 mmol/L Disodium Edetate (Sodium Ethylene Diamine Tetraacetate), 0.2 mg/mL Polysorbate 80, pH 6.0.

The minimum packaging unit of Sintilimab is a box, which contains 2 vials of Sintilimab (IBI308) injection liquid packed in a vial. The box is labeled with the drug name, dosage form, specifications, drug code, batch number, expiration date, storage conditions, and sponsor information. The labels on the vials and the box have the same information, but the vial labels do not have information on dosage form, precautions, and usage and

administration. All box and vial labels are markedFor Clinical Study Use Only. Sintilimab should be stored in the dark at 2~8°C, with a shelf life of 24 months.

If the injection shows turbidity, precipitation, or other quality issues, it should be immediately sealed and the sponsor notified.

The preparation and infusion process of Sintilimab is as follows:

1. Completely withdraw the injection liquid from 2 vials of Sintilimab and add it to a 100mL 0.9% (weight/volume) Sodium Chloride sterile saline intravenous infusion bag, and record the start time of preparation.
2. Gently invert the infusion bag to mix, ensuring the uniformity of the medication in the infusion bag, avoiding vigorous agitation to produce foam. If a large amount of foam is produced, the medication should be left to stand until the foam dissipates.
3. Administer the medication through an inline filter with a pore size of 0.2~1.2μm (the recommended infusion time is controlled within 30-60 minutes), and record the start and end times of the administration. Precautions: Confirm that the Sintilimab injection is transparent and free from turbidity, precipitation, or other quality issues before preparation; Ensure that the time from the first puncture and extraction of the Sintilimab injection to the end of the administration does not exceed 24 hours (the prepared medication should be stored under refrigeration at 2~8℃); Avoid mixing with other medications; Avoid intravenous bolus injection.

5.1.2 Use of Chemotherapy Medications

The standard chemotherapy drugs used in combination are prepared according to the descriptions in the approved product instructions, and the selected doses are as follows:

- Etoposide: 100mg/m^2 d1-3/q3w
- Cisplatin: 75mg/ m^2 d1/q3w
- Carboplatin: AUC 5 (calculated using the Calvert formula) d1/q3w, the dose of Carboplatin must not exceed 750mg.

Calvert Formula:

Total dose (mg) = (Target AUC) × (CrCl + 25)

The estimated CrCl used in the Calvert formula must not exceed 125ml/min.

Maximum Carboplatin dose (mg) = Target AUC 5 (mg·min/ml) × (125 + 25) = 5 × 150 ml/min = 750mg.

To prevent the nephrotoxicity of Cisplatin, adequate hydration is required: Hydration for 3 days is needed when using Cisplatin, along with potassium chloride, mannitol, and furosemide (Lasix), to maintain a daily urine volume of 2000-3000ml. Cisplatin is a highly emetogenic chemotherapeutic drug; it is recommended to use 5-HT3 receptor antagonists,

dexamethasone (or other glucocorticoids), dopamine receptor blockers (metoclopramide), antihistamines (such as diphenhydramine, benzhexol, etc.), and under conditionally, NK-1 receptor antagonists (aprepitant) to prevent chemotherapy-related vomiting.

Table 3 Study Treatment Medications

Drug	Dose/Amount	Administration Frequency	Method of Administration	Treatment Course/Cycle
Sintilimab	200mg	Q3W	Intravenous infusion	Weekly on day 1 of every 21-day cycle, up to 2 years
Carboplatin	AUC 5	Q3W	Intravenous infusion	Weekly on day 1 of every 21-day cycle, maximum of 6 cycles
Cisplatin	75mg/m^2	Q3W	Intravenous infusion	Weekly on day 1 of every 21-day cycle, maximum of 6 cycles
Etoposide	100mg/m^2	Q3W	Intravenous infusion	Weekly on day 1 of every 21-day cycle, maximum of 6 cycles

1. Sintilimab should be administered before the chemotherapy drugs.

2. If the subject's weight fluctuates by less than 10% from the baseline (the first dose of study treatment day), the baseline weight is used to calculate the body surface area (BSA), and the dosage of chemotherapy drugs is calculated accordingly. Conversely, the actual weight on the planned dosing day is used to calculate the chemotherapy drug dosage. The protocol allows a maximum BSA of 2.0 m^2. For subjects with BSA > 2.0 m^2, the research center staff will calculate based on a BSA of 2.0 m^2. For convenience in dosing, the protocol permits a ±5% deviation from the calculated total dose for each infusion.

5.2 Dose Adjustments

5.2.1 Dose Adjustments for Sintilimab

- The dose of Sintilimab is not to be adjusted throughout the study. The principles for withholding and permanently discontinuing Sintilimab are outlined in Table 4. If an administration delay occurs during a 3-week cycle of Sintilimab treatment, all future dosing dates will be postponed to ensure that the dosing intervals between cycles of Sintilimab treatment are within 21 ± 3 days. Sintilimab-related adverse events are typically immune-related AEs. For pauses in administration due to immune-related AEs, based on the type and severity of the AE, corticosteroid treatment can be administered to alleviate to grade 1 or 0, and then the corticosteroids can be tapered over more than 4 weeks, after which the administration can be resumed.

Table 4: Dose Adjustment Scheme for Sintilimab-Related Immune Adverse Events

Immune-Related Adverse Events	Severity	Dose Adjustment
Pneumonia	Grade 2	Hold treatment[a]
	Recurrent Grade 2, Grade 3 or 4	Permanent discontinuation
Diarrhea/Enterocolitis	Grade 2 or 3 Diarrhea or Enterocolitis	Hold treatment[a]
	Grade 4 Diarrhea or Enterocolitis	Permanent discontinuation
Dermatitis	Grade 3 Dermatitis	Hold treatment[a]
	Grade 4 Dermatitis	Permanent discontinuation
Hepatitis	For subjects with normal baseline ALT, AST, or TBIL, Grade 2 elevation of AST, ALT, or TBIL; For subjects with baseline AST, ALT, or TBIL > ULN, an increase of AST, ALT, or TBIL ≥ 50% (meeting Grade 2 requirements) and duration < 7 days	Hold treatment[a]
	For subjects with baseline ALT, AST, or TBIL within	Permanent discontinuation

	normal limits, Grade 3 or Grade 4 elevation of AST, ALT, or TBIL; for subjects with baseline AST, ALT, or TBIL > ULN (Upper Limit of Normal), an increase in AST, ALT, or TBIL by ≥50% (meeting the criteria for Grade 3 or Grade 4) and duration ≥7 days	
pituitary inflammation	Grade 2	Hold treatment
	Grade 3 or Grade 4	Hold treatment or Permanent discontinuation
adrenal insufficiency	Grade 2	Hold treatment
	Grade 3 or Grade 4	Hold treatment or Permanent discontinuation
hyperthyroidism	Grade 3 or Grade 4	Hold treatment or Permanent discontinuation
hypothyroidism	Grade 2-4	Continue treatment
Type 1 diabete	Grade 3 hyperglycemia	Hold treatment
	Grade 4 hyperglycemia	Hold treatment or Permanent discontinuation
renal insufficiency	Grade 2 or Grade 3 creatinine elevation	Hold treatment[a]
	Grade 4 creatinine elevation	Permanent discontinuation
neurotoxicity	Grade 2	Hold treatment[a]
	Grade 3 or Grade 4	Permanent discontinuation
other immune-related AEs (Adverse Events)	intolerable/persistent Grade 2 AE	Hold treatment[a]
	Grade 3 AE	Hold treatment or Permanent discontinuation
	Grade 4 AE or recurrent Grade 3 AE	Permanent discontinuation

a: Treatment can be resumed once symptoms improve to Grade 0-1 or baseline levels.

b: Pituitary inflammation, adrenal insufficiency, hypothyroidism/thyroid dysfunction, and Type 1 diabetes can be reinitiated if adequately controlled and only physiological hormone replacement therapy is required.
c: For abnormal laboratory test results, the decision to hold or permanently terminate treatment should be based on accompanying clinical symptoms/signs and the clinical judgment of the investigator.

The maximum duration for holding Sintilimab treatment is 12 weeks. If the subject cannot recover to a state where Sintilimab can be reused within 12 weeks, the subject will permanently discontinue Sintilimab and enter the follow-up phase. Exceptions include the following two situations:

- If the use of glucocorticoids for treating irAE (immune-related adverse events) causes the hold of Sintilimab to exceed 12 weeks. In this case, it is necessary to discuss with the sponsor's medical manager to decide whether to continue. The imaging assessments for efficacy evaluation should proceed as planned without being affected by the suspension of the medication.
- Continuation of Sintilimab Treatment: In cases where the treatment is paused due to adverse events (AEs) not related to Sintilimab, the imaging assessments for efficacy evaluation should proceed as planned without being affected by the suspension of the medication.

5.2.2 Management of Sintilimab-Related Infusion Reactions

Sintilimab may cause severe or life-threatening infusion reactions, including severe hypersensitivity or allergic reactions. Signs and symptoms typically appear during or shortly after the infusion and can usually be completely resolved within 24 hours after the completion of the infusion. The management guidelines for infusion reactions related to Sintilimab are presented in Table 5.

Table 5: Management Guidelines for Sintilimab Infusion Reactions

NCI CTCAE Grade	Treatment	Premedication for Subsequent Dosing
1<br>Mild reaction; no interruption of infusion is needed; no intervention required.	According to the medical indications of the patient, monitor vital signs until the investigator deems the subject's condition stable.	No specific premedication required.
2<br>Treatment or interruption of	Stop the infusion and monitor symptoms. Other	The subject may receive the following premedication

infusion may be necessary, but symptomatic treatment (such as antihistamines, nonsteroidal anti-inflammatory drugs [NSAIDs], analgesics, intravenous fluid replacement) should be initiated promptly and a response is expected within ≤24 hours; prophylactic medication may be required for up to 24 hours.	appropriate medications may include but are not limited to: intravenous fluids, antihistamines, NSAIDs, acetaminophen, analgesics.<br><br>According to the medical indications of the patient, monitor vital signs until the investigator deems the subject's condition stable.<br><br>If symptoms resolve within one hour after stopping the infusion, the infusion may be resumed at 50% of the original infusion rate (for example, from 100 mL/hr to 50 mL/hr). Otherwise, the medication should be suspended until symptoms resolve, and the subject should receive premedication before the next scheduled dosing. For subjects who still experience grade 2 toxicity after adequate premedication, further study drug treatment should be permanently discontinued.	1.5 hours (±30 minutes) before the infusion of Sintilimab:<br><br>Oral diphenhydramine 50 mg (or an equivalent dose of an antihistamine).<br><br>Oral acetaminophen 500-1000 mg (or an equivalent dose of an antipyretic).
Grade 3 or 4 Reactions:<br>Grade 3:	Stop the infusion.<br>Other appropriate	No further dosing.

Prolonged duration (i.e., failure to respond quickly after symptomatic treatment and/or brief interruption of infusion); symptoms recur after initial improvement; hospitalization required due to other clinical sequelae (such as renal impairment, pulmonary infiltration).<br><br>Grade 4:<br>Life-threatening; requiring pressor agents or ventilatory support.	medications may include but are not limited to:<br>- Epinephrine<br>- Intravenous fluids<br>- Antihistamines<br>- Nonsteroidal anti-inflammatory drugs (NSAIDs)<br>- Acetaminophen<br>- Analgesics<br>- Oxygen<br>- Pressor agents<br>- Corticosteroids<br><br>Closely monitor the patient's vital signs according to medical indications until the investigator deems the subject's condition stable. Hospitalization may be required.<br><br>In the event of an allergic reaction, administer epinephrine immediately.<br>The subject should permanently discontinue further study drug treatment.	
Appropriate emergency equipment should be available in the ward, and a physician should be readily accessible during the administration period. For further information, please refer to the Common Terminology Criteria for Adverse Events (CTCAE) version 5(http://ctep.cancer.gov).		

5.2.3 Dose Adjustment of Chemotherapy Drugs:

If the investigator confirms that the toxic reaction is mainly caused by a single chemotherapy drug, a reduction in the dose of that single drug is acceptable; if it is not possible to clearly attribute the toxic reaction to two or more drugs, a reduction in all drugs considered relevant may be made simultaneously. If the administration of chemotherapy is delayed during a treatment cycle of every 3 weeks, all future dosing dates will be postponed to ensure that the dosing interval between chemotherapy cycles is 21±3 days. The chemotherapy cycle and the Sintilimab cycle should be synchronized as much as possible by adjusting the allowed window period. The maximum allowed period of discontinuation for chemotherapy drugs is 6 weeks from the last chemotherapy interval; if the requirements for chemotherapy administration cannot be met after more than 6 weeks, the chemotherapy should be permanently discontinued.

The following are the different dose levels for chemotherapy drugs. If a further dose reduction is required after reducing by two dose levels, discontinue the chemotherapy drug.

Table 6: Dose Adjustment Levels for Chemotherapy Drugs

	**Original Dose**	**One Dose Level Reduction**	**Two Dose Levels Reduction**
Etoposide	100mg/m^2	75 mg/m^2	50 mg/m^2
Cisplatin	75mg/m^2	56mg/m^2	38mg/m^2
Carboplatin	AUC 5, maximum dose, 750mg	AUC 3.75, maximum dose 562.5mg	AUC 2.5, maximum dose 375mg

Table 7: Hematologic Toxicity Adjustments

**Platelets**	**Neutrophils**	**Dose Adjustment According to Table 6**
≥50×10^9/L and	≥0.5×10^9/L	Original dose
≥50×10^9/L and	<0.5×10^9/L	Reduce one dose level
<50×10^9/L , no bleeding and	any condition	Reduce one dose level
<50×10^9/L,	and any condition	Reduce two dose levels

with bleeding		
Any condition and	<1×10^9/L with fever ≥38.5°C	Reduce one dose level

Table 8: Non-hematologic Toxicity

**Adverse Event**	**CTCAE Grade**	**Cisplatin**	**Carboplatin**
Nausea or Vomiting	Grade 3 or 4	Original dose	Original dose
Diarrhea	Grade 3 or 4	Reduce one dose level	Original dose
Neuropathy	Grade 2	Reduce two dose levels	Original dose
	Grade 3 or 4	Discontinue treatment	Reduce one dose level
Transaminase Elevation	Grade 3	Reduce one dose level	Reduce one dose level
	Grade 4	Discontinue treatment	Discontinue treatment
Other Non-hematologic Toxicity	Grade 3 or 4	Reduce one dose level	Reduce one dose level

Etoposide Dose Adjustment

Dose adjustments and delays are permitted. Adjustments are based on the product information and clinical guidelines, with recommended adjustments as shown in Table 9. If the investigator, considering the benefit/risk ratio of the subject, believes that they cannot follow the operations listed in the table or encounters a situation not listed in the table, and must suspend or resume etoposide, they should discuss and decide with the sponsor's investigator.

Creatinine Clearance (CrCl): CrCl is calculated using the Cockcroft-Gault formula based on the standard body weight (Appendix 2). Before the first administration of chemotherapy drugs, CrCl must be ≥50mL/min.

Table 9: Etoposide Dose Adjustment

**Creatinine Clearance**	**Etoposide Dose**
>50	100%

15-50	75% of the original dose

5.3 Principles for Managing Toxicity of Immune Checkpoint Inhibitors

Adverse events (AEs) related to exposure to Sintilimab may have an immunological etiology. These immune-related AEs (irAEs) may occur shortly after the first dose or months after the last dose of Sintilimab, and can potentially affect more than one body system simultaneously. Therefore, early detection and initiation of treatment are crucial for reducing complications. Based on existing clinical trial data, most irAEs are reversible and can be managed through discontinuation of Sintilimab, administration of glucocorticoids, and/or other supportive therapies. For suspected irAEs, ensuring appropriate assessment to confirm the etiology or exclude other causes is essential. Additional procedures or tests such as bronchoscopy, endoscopy, or skin biopsy may be included as part of this assessment. Depending on the severity of the irAE, treatment with Sintilimab may be paused or permanently discontinued, and glucocorticoids may be administered. Guidelines for dose adjustments and toxicity management for potential irAEs can be found in theManagement Manual for Immune-related Adverse Events provided by the sponsor.

5.4 Concurrent Therapies

5.4.1 Permissible Concurrent Therapies

- Medications deemed to be in accordance with the protocol by the investigator (e.g., for treating disease-related symptoms and various AEs associated with treatment).
- Subjects requiring long-term medication for underlying conditions such as hypertension or diabetes may continue their medication.
- Supportive therapy for alleviating tumor-related symptoms is permitted, such as bisphosphonate treatment for bone metastases.
- The use of local glucocorticoids is allowed, such as topical application to the skin, eye drops, nasal sprays, and inhalation.

5.4.2 Prohibited Concurrent Therapies

Subjects are prohibited from receiving the following treatments during the study treatment period:

- Systemic chemotherapy or biological therapy with antitumor activity (cytokine drugs used to treat adverse events caused by chemotherapy drugs are excluded), as well as traditional Chinese medicine with antitumor activity.
- Medications with immunomodulatory effects, including but not limited to non-specific immunomodulators (such as thymosin, interferons, interleukins, immunoglobulins, and gamma globulins), as well as traditional Chinese medicine with immunomodulatory effects.

- Chemotherapy drugs not specified in this protocol.
- Investigational drugs other than the study medication.
- Radiation Therapy for Tumor Control:Palliative radiation therapy is permitted as long as it is not directed at target lesions, such as for alleviating bone metastasis pain or brain metastasis symptoms.
- Live Vaccines within 30 Days Prior to the First Administration of the Study Drug and During the Study: Live vaccines, including but not limited to measles, mumps, rubella, varicella, yellow fever, rabies, BCG vaccine, and typhoid (oral) vaccine, are not allowed during the study. Inactivated influenza virus vaccines for seasonal influenza are permitted, but live attenuated influenza vaccines administered intranasally are not allowed.
-Corticosteroids: Inhaled corticosteroids are permitted for patients with asthma or chronic obstructive pulmonary disease (COPD).Temporary use of corticosteroids for alleviating respiratory distress is allowed.Corticosteroids are permitted for managing immune-related adverse events (irAEs).The use of physiologic doses of corticosteroids may be approved after consultation with the sponsor.
- Note: Prophylactic corticosteroids to prevent allergic reactions (e.g., premedication before intravenous contrast media or chemotherapy administration) are allowed.

Exclusion Criteria:

Subjects who, according to the investigator's assessment, require any of the above treatments for clinical therapy should be excluded from the trial. Subjects may receive other medications deemed medically necessary by the investigator.

Investigator's Review:

It is crucial for the investigator to review each medication (prescription and over-the-counter) received by the subjects before the start of the study and at each study visit.
- At each visit, subjects must be asked about any new medications they have taken.
- To reduce the risk of adverse drug interactions, all measures must be taken to limit the number of truly necessary concomitant medications.
- During the administration period, medications with hepatotoxicity (i.e., those with warnings of hepatotoxicity in the product information) should be avoided. Investigators are encouraged to review each potential hepatotoxic medication by accessing the website www.livertox.nih.gov.
- The use of medications listed in the exclusion criteria is not allowed.

5.5 Drug Administration

5.5.1 Drug Management

Medications used in the study shall be stored and distributed by designated personnel, ensuring that the transportation temperature of the drugs is within the specified range. After verification, they should be signed for and stored at the prescribed temperature. If temperature anomalies occur during transportation or storage at the research center, the drugs should be promptly transferred to the specified temperature, not administered to subjects temporarily, and reported to the sponsor in a timely manner for handling according to the sponsor's opinion.

All investigational drugs provided by the sponsor are for use in this study only and shall not be used for purposes outside the scope of this protocol. Investigators must commit not to provide study drugs to any individuals not related to this study.

5.5.2 Disposal and Destruction of Study Medication

In this study, used containers of Sintilimab must be recycled, and containers of chemotherapy drugs may be destroyed on-site according to the applicable guidelines and operating procedures established by the research center and local institutions. If some research centers have difficulty recycling Sintilimab containers, with the sponsor's consent, it is permissible to destroy them on-site following the same procedures as for chemotherapy drug containers.

All unused study medications must be collected and returned to the sponsor for centralized destruction after the completion/termination of the study or after the expiration date. The collection of study medication is arranged by the clinical monitor designated by the sponsor.

5.6 Documentation of Study Medication

Designated personnel at the research center must keep timely records of the receipt, distribution, use, inventory, destruction, recycling, and disposal of study medication in accordance with relevant regulations and guidelines.

5.7 Complaint Handling

To ensure the safety of study participants and the quality of monitoring, and to assist in the improvement of processes and medicinal products, the sponsor will collect product complaints related to the study medication used in clinical trials.

Complaints related to concomitant medications will be reported directly to the manufacturer according to the product description.

The investigator or their designated personnel are responsible for completing the following product complaint process in accordance with the relevant provisions of this study:

- Use the study-specific complaint form to record the reported product complaints and a complete description.
- Fax or email the completed product complaint form to the sponsor or their designated personnel within 24 hours.

If the investigator is required to return the product for investigation, the investigator should return the product along with a copy of the product complaint form.

6 Study Process

Before starting the study, patients must read and sign the informed consent form approved by the current Ethics Committee (EC).

Various examinations and test procedures should be carried out according to the schedule of the study process, regardless of the duration of drug withdrawal. However, adjustments within the window period of each examination item are allowed due to holidays, public holidays, or other administrative reasons.

6.1 Subject Screening

6.1.1 Subject Screening

The investigator will enroll subjects in the following steps:

1. Subjects sign the informed consent form before any study-related procedures are conducted.
2. The principal investigator or a designated person trained accordingly reviews the inclusion/exclusion criteria and officially determines the eligibility of the subject for inclusion.

Patients who do not meet the eligibility criteria for this study (screening failure) may be rescreened. If rescreening of a patient is considered, the investigator must contact the sponsor's medical manager. Each patient may be rescreened once. When rescreening, the patient must sign a new Informed Consent Form (ICF) and will be assigned a new identification number.

6.2 Study Plan and Scheduling

6.2.1 Screening Period

During the screening period (Days -28 to -1), the following study procedures must be completed to ensure that the subject is eligible for this study:

- Signature of the Informed Consent Form
- Verification of inclusion/exclusion criteria
- Documentation of demographic data, medical history, and prior treatment history for lung cancer
- Concomitant medications
- Recording of vital signs

- Height and weight
- Physical examination
- 12-lead electrocardiogram (ECG)
- Echocardiogram
- Cardiac enzyme profile
- Complete blood count/blood chemistry/urinalysis (within 7 days prior to the first dosing)
- Pregnancy test (within 3 days prior to the first dosing)
- Thyroid function tests (within 28 days prior to the first dosing)
- Virology antibody tests (HIV, HBV, and HCV) (within 28 days prior to the first dosing)
- ECOG Performance Status (PS) score
- Assessment of adverse events
- Tumor imaging assessment (within 28 days prior to the first dosing)
- Archived or fresh tumor tissue samples

For detailed descriptions of the physical examination, tumor imaging assessment, and safety assessment, refer to sections 7.1 and 7.2.

6.2.1.1 Baseline (Before Dosing on Day 1 of Cycle 1)

- Concomitant medications
- Recording of vital signs
- Weight
- Physical examination
- ECOG PS Score
- Adverse Event Assessment
- Quality of Life Questionnaire
- Biomarker Blood Specimen Collection

6.2.2 Treatment/Maintenance Treatment Visits

- Concomitant medications
- Recording of vital signs and body weight
- Physical examination
- ECOG Performance Status (PS) score
- 12-lead electrocardiogram (ECG)
- Echocardiogram
- Cardiac enzyme profile
- Complete blood count/blood chemistry/urinalysis
- Thyroid function tests

- Adverse event assessment
- Tumor imaging assessment
- Administration of study medication
- Quality of life questionnaire
- Collection of biomarker whole blood samples

Refer to Table 1 for the treatment/maintenance treatment visit flowchart. Detailed descriptions of tumor imaging assessments and safety assessments can be found in sections 7.1-7.3.

6.2.3 Safety Follow-up Visit

A safety follow-up visit will be conducted 30 (±3 days) after the last drug administration, or before the start of a new antitumor treatment, whichever occurs first. It includes the following:

- Concomitant medications
- Recording of vital signs
- Body weight
- Physical examination
- 12-lead electrocardiogram (ECG)
- Complete blood count/blood chemistry/urinalysis
- Thyroid function tests
- Pregnancy test
- ECOG Performance Status (PS) score
- Adverse event assessment
- Record Subsequent Anticancer Treatments (if applicable)
- Quality of life questionnaire

All adverse events (AEs) occurring prior to the safety follow-up visit should be recorded until they resolve to grade 0-1 or the baseline level, or the investigator deems further follow-up unnecessary for valid reasons (e.g., not recoverable or has improved), whichever comes first.

6.2.4 Survival Visits

After the safety follow-up, contact the subject every 90 days (±7 days) (telephone visits are acceptable), to obtain as much information as possible related to survival, as well as any subsequent systemic anticancer treatments and disease progression (for subjects without radiographic progression). Long-term follow-up will continue until the subject's death or the end of the study.

6.2.5 Subsequent Anticancer Treatment Status

All new anticancer treatments initiated after the last drug administration should be reviewed by the investigator or a qualified designated personnel. If a subject starts a new anticancer treatment within 30 days after the last administration of the trial treatment, a safety follow-up visit must be conducted before the first administration of the new treatment.

After starting a new anticancer treatment, the subject will enter the survival follow-up. For detailed instructions on survival status follow-up, please refer to Section 6.2.4 - Survival Visits.

6.3 Other Procedures

6.3.1 Discontinuation of Treatment/Withdrawal from the Study

Subjects who discontinue treatment/withdraw from the study before completion should be encouraged to continue follow-up and complete all remaining study visits.

When a subject discontinues treatment/withdraws from the study, all procedures applicable to the end-of-treatment visit should be conducted. Any adverse events present at the time of discontinuation/withdrawal should be followed up in accordance with the safety requirements outlined in Section 8.4 (Recording of Adverse Events). If a patient discontinues treatment/withdraws from the study for reasons other than objective disease progression, patients should undergo imaging assessments at the end of treatment. For subjects who have completed 24 months of Sintilimab treatment, treatment may be discontinued. After discontinuing treatment following 24 months of Sintilimab therapy, subjects should return to the research center for a safety follow-up visit, and then enter the follow-up phase of this study.

6.3.2 Loss to Follow-up

If a subject fails to return to the clinic for required study visits and/or if the research center is unable to contact the subject, the following procedures will be implemented:

- The research center must attempt to contact the subject and reschedule the missed visits. If contact is made, the importance of maintaining the visit schedule as stipulated in the protocol should be communicated.
- For each missed visit, the investigator or designated personnel must make every effort to reestablish contact with the subject (e.g., by telephone and/or by sending a registered letter to the last known mailing address of the subject or equivalent local methods). These attempts at contact should be documented in the subject's medical record.

Note: A subject is not considered lost to follow-up until after the last scheduled visit for the individual subject has been reached.

The amount of missing data from subjects should be managed in accordance with

predefined guidelines for data processing and analysis.

7 Study Evaluations

7.1 Efficacy Assessment

7.1.1 Baseline Tumor Imaging Assessment

The initial tumor imaging examination at screening should be conducted within 28 days prior to the first administration of the study drug. Prior to the first administration, the investigator at the research center confirms that the subject has measurable lesions in accordance with RECIST 1.1 criteria and records the baseline tumor status.

The method used for tumor burden assessment at baseline must be consistent with the method used for each subsequent follow-up assessment (CT/MRI). Imaging examinations of other suspected involved areas (such as the skull) may be performed based on the subject's clinical symptoms and signs. Subjects known or suspected to have brain metastases at screening should undergo baseline skull CT/MRI before the start of study treatment. During the study, subjects with this condition should undergo skull CT/MRI assessments, with brain metastases lesions being evaluated as non-target lesions.

7.1.2 Tumor Imaging Assessment During the Study

Tumor imaging evaluations will be conducted every 6 weeks (±7 days) following the first dose of the study medication, and after 24 weeks of treatment, evaluations may be conducted every 9 weeks (±7 days) until disease progression, initiation of new anticancer treatment, subject withdrawal of ICF, or death. Patients who first record radiographic disease progression and meet specific conditions may continue treatment after disease progression.

If during the study, patients exhibit clinically unstable disease, unplanned imaging assessments can be conducted at any time.

The definition of clinically unstable disease is as follows:

- The appearance of clinically significant symptoms and signs indicating disease progression (including the deterioration of laboratory test values)
- A decrease in the ECOG PS score
- Rapid disease progression
- Tumor progression in important anatomical locations requiring other urgent medical interventions (such as spinal cord compression)

For patients who discontinue treatment for reasons other than radiographic disease progression, imaging assessments should be performed at the end of treatment, and then continue with imaging evaluations at the scheduled time points according to the protocol, until any of the following events occur: initiation of new anticancer treatment, objective

disease progression, subject withdrawal of ICF, loss to follow-up, or death.

If the investigator is unable to determine whether the disease has progressed, especially if there is uncertainty regarding non-target lesions and new lesions, subjects may continue treatment and undergo repeat imaging assessments when clinical symptoms occur or at the next scheduled assessment time. If disease progression is confirmed, the date of progression should be the initial date of discovery.

Tumor imaging assessments in this study are based on the investigator's use of RECIST 1.1, and the assessment method is detailed in Appendix 3.

7.1.3 Tumor Imaging Examinations at the End of Treatment and Follow-up Period

For subjects who complete treatment or discontinue treatment for reasons other than objective disease progression, a tumor imaging assessment should be performed at the time of treatment completion/cessation. Subsequent imaging assessments should continue according to the schedule specified in the protocol until one of the following occurs: initiation of a new anticancer treatment, objective disease progression, death, or study completion, whichever comes first.

7.2 Safety Assessment

Investigators or qualified designated personnel assess adverse events in each subject during the study and follow-up period as required by the study flowchart, grade and record adverse events according to NCI CTCAE (version 5.0).

Determination of Adverse Event Characteristics:

Adverse events are characterized based on severity, causality, toxicity grading, and actions taken in response to the trial treatment.

All adverse events of unknown etiology occurring during the study treatment must be evaluated to determine if they are immune-related adverse events.

For detailed instructions on the assessment and recording of adverse events, please refer to Section 8.

7.2.1 Physical Examination:

A complete physical examination includes general condition, respiratory system, cardiovascular system, abdomen, skin, head and neck (including ears, eyes, nose, and throat), lymph nodes, thyroid, musculoskeletal system (including spine and extremities), genital/anal, and neurological assessment.

7.2.1.1 Height, Weight, and Vital Signs:

Height is measured only during the screening period. Weight is measured as needed prior to each scheduled dosing during the study. Investigators or qualified designated personnel will record vital signs as indicated in the study flowchart. Vital signs include body

temperature, pulse rate, respiratory rate, and blood pressure. Investigators may decide to perform additional vital sign assessments at their discretion according to standard clinical practice or based on clinical needs. Additional vital sign recordings may be taken in the medical record when an adverse event/serious adverse event occurs (if applicable). The date and time of collection and measurement will be recorded in the appropriate section of the medical record.

7.2.1.2 12-Lead Electrocardiogram (ECG):

A standard 12-lead ECG is recorded once during the screening period following local standard procedures. Clinically significant abnormal results should be documented. ECG examinations may be performed at other time points based on clinical needs.

7.2.1.3 Eastern Cooperative Oncology Group (ECOG) Performance Status Score:

Investigators or qualified designated personnel will assess the ECOG performance status as indicated in the study flowchart at screening, prior to dosing on Day 1 of each treatment cycle, at treatment discontinuation, and during the safety follow-up period.

7.2.2 Laboratory Test Assessments:

The laboratory tests are listed below. The procedures for the collection of body fluid specimens are described in the Study Procedures Manual. The timing of the tests is referenced in the laboratory assessment section of the study flowchart.

7.2.2.1 Laboratory Safety Evaluation (Complete Blood Count, Coagulation Profile, Urinalysis, Blood Biochemistry, etc.)

Laboratory test items for analyses such as complete blood count, urinalysis, and blood biochemistry are presented in Table 10.

Table 10 Routine Laboratory Safety Assessment

Complete Blood Count	RBC, HGB, WBC, PLT, LYM, ANC
Blood Biochemistry	TBIL, ALT, AST, γGT, ALP, ALB, TP, LDH, BUN, Cr, Na, K, Cl, Mg, Ca, P, Amylase, and FBG
Urinalysis	PH, UALB, UPRO, URBC, and UGLU

7.2.2.2 Pregnancy Test

All females considering participation in the trial who have not undergone surgical sterilization or are not postmenopausal must undergo a pregnancy test within 3 days prior

to the first administration of the study drug. If the urine test is indeterminately negative, a serum test is required. Subjects must be excluded/terminated in cases of a positive or borderline positive test result.

7.3 Quality of Life Assessment

The quality of life assessment utilizes the EORTC QLQ-C30 questionnaire, which is administered on the day of the first dosing, at each imaging assessment, and at the first safety follow-up visit. If an unplanned imaging assessment is conducted, the quality of life questionnaire should also be administered concurrently.

The EORTC QLQ-C30 is a core questionnaire for all cancer patients, comprising 30 items across 15 dimensions, including 5 functional scales (physical, role, cognitive, emotional, and social functioning), 3 symptom scales (fatigue, pain, and nausea/vomiting), one global health/status of life quality dimension, and 6 single items.

7.4 Storage and Destruction of Biological Samples

Samples will be disposed of or destroyed and subjected to a process of combined anonymization. Additional analyses may be conducted on the anonymized, combined samples to further evaluate and validate the analytical methods. Any results obtained from these analyses may be reported separately from the CSR.

Sample reproducibility analyses (if performed) will be conducted in parallel with the bioanalysis of the trial samples. The results of these assessments will not be reported in the clinical study report but will be presented separately in a bioanalytical report.

8 Safety Reporting and Adverse Event Management

8.1 Definition of Adverse Events

An Adverse Event (AE) is defined as any unfavorable and unintended medical occurrence in a subject of a clinical investigation that takes place from the time of signing the informed consent form, whether or not considered related to the investigational product. AEs include, but are not limited to, the following situations:

Investigators should record in detail any adverse events that occur to a subject, including: description of the adverse event and all related symptoms, time of onset, severity, cause of the adverse event, relationship to the trial medication, duration, measures taken, and final outcome and resolution.

8.2 Criteria for Assessing the Severity of Adverse Events

CTCAE Version 5.0 Grading

- Grade 1: Mild; asymptomatic or mild symptoms; only observed clinically or diagnostically; no treatment required
- Grade 2: Moderate; requires minimal, local, or non-invasive treatment; limitation of instrumental activities of daily living appropriate for age
- Grade 3: Severe or medically significant but not immediately life-threatening; hospitalization or prolongation of hospital stay required; disability; limitation of self-care activities of daily living
- Grade 4: Life-threatening; requires urgent treatment
- Grade 5: Death related to the AE

8.3 Criteria for Assessing the Relationship between Adverse Events and the Investigational Medication

Adverse events include all unexpected clinical manifestations that should be reported as AEs, regardless of whether they are related to the trial medication, whether the subject is assigned to the medication group, or even whether the medication is administered. All adverse events must be reported in the form of a clinical report. Any discomfort complained of by the subject during the treatment period, or any abnormal changes in objective laboratory test indicators, should be truthfully recorded, along with the severity, duration, treatment measures, and outcome of the adverse event. The clinical physician should combine medical knowledge, clinical experience, and the safety characteristics of the medication to conduct a comprehensive analysis and judgment of the possible relationship between adverse events and the trial medication, using a five-level classification ofdefinitely related, likely related, possibly related, possibly unrelated, and unrelated.Definitely related,likely related, andpossibly related are all classified as adverse drug reactions. When calculating the incidence of adverse reactions, these three categories are combined as the numerator, and the total number of subjects evaluated for safety is used as the denominator.

Table 11 Criteria for Assessing the Relationship between Adverse Events and the Investigational Medication

Causality	Criteria for Assessment
**Related**	• The occurrence of the adverse event is temporally related to the administration of the medication.<br>• The investigational medication is a more plausible explanation for the adverse event than other causes

	(e.g., the subject's pre-existing conditions, environmental or toxic factors, or other treatments the subject has received).<br>• The adverse event resolves or decreases after discontinuation or dose reduction of the medication.<br>• It is consistent with known AE types of the suspected drug or its pharmacological class.<br>• The adverse event reappears upon re-administration of the medication.
**Possibly Related**	• The occurrence of the adverse event is temporally related to the administration of the medication.<br>• The investigational medication shares equal plausibility with other causes (e.g., the subject's pre-existing conditions, environmental or toxic factors, or other treatments the subject has received) in explaining the adverse event.<br>• The adverse event resolves or decreases after discontinuation or dose reduction of the medication, if applicable.
**Unlikely Related**	• Other causes (e.g., the subject's pre-existing conditions, environmental or toxic factors, or other treatments the subject has received) are more plausible explanations for the adverse event than the investigational medication.<br>• The adverse event does not resolve or decrease after discontinuation or dose

	reduction of the medication, if applicable, or the situation is unclear.<br>• The adverse event does not reappear upon re-administration of the medication, or the situation is unclear.
**Definitely Unrelated**	• The occurrence of the adverse event is not temporally related to the administration of the medication, or<br>• The adverse event has another obvious cause (e.g., the subject's pre-existing conditions, environmental or toxic factors, or other treatments the subject has received).
**Unable to Determine**	• The information above is insufficient, and based on the available information, the investigator believes it is impossible to determine, and the investigator cannot obtain further follow-up information.

8.4 Serious Adverse Events (SAEs)

Serious adverse events are medical events that occur during the clinical trial process and require hospitalization or prolongation of hospital stay, disability, affect the ability to work, are life-threatening, or result in death, congenital malformations, etc. These include the following unexpected medical events:

- Events that result in death;
- Life-threatening events (defined as events where the subject is at risk of death at the time of occurrence);
- Events that require hospitalization or prolongation of hospital stay;
- Events that result in permanent or significant disability/incapacity/affecting the ability to work;
- Congenital anomalies or birth defects.

8.4.1 Pregnancy

Pregnancies occurring during the clinical trial should be reported as serious adverse events, along with a specializedPregnancy Report Form.

8.4.2 Disease Progression

Disease progression (including symptoms and signs of progression) should not be reported as serious adverse events. However, if death occurs due to disease progression within the trial or safety reporting period, it should be reported as a serious adverse event. Hospitalization due to symptoms and signs of disease progression should not be reported as a serious adverse event. If the final outcome of cancer is death within the trial or safety reporting period, the event leading to death must be reported as a serious adverse event.

8.4.3 Undertaking Other Anticancer Treatments

Recording of adverse events begins from the start of medication administration until 30 days after discontinuation. If subjects start other anticancer treatments after 30 days, there is no need to continue recording and tracking non-fatal adverse events. If death occurs within the serious adverse event reporting period after the end of study treatment, it must be reported regardless of whether the patient has received other treatments.

8.4.4 Hospitalization

Adverse events that result in hospitalization or prolongation of hospital stay during clinical research should be considered serious adverse events. Any initial admission to a medical institution (even if less than 24 hours) meets this criterion.

Hospitalization does not include the following situations:

- Rehabilitation institutions
- Convalescent homes
- Routine emergency room admissions
- Same-day surgeries (e.g., outpatient/same-day/non-hospitalized surgeries)

Hospitalization or prolongation of hospital stay not related to the worsening of adverse events (AEs) itself is not a serious adverse event (SAE). For example:

- Hospitalization due to a pre-existing condition without the occurrence of new AEs or exacerbation of the existing condition (e.g., for the examination of laboratory test abnormalities that have persisted since before the trial)
- Hospitalization for administrative reasons (e.g., routine annual medical examinations)
- Hospitalization stipulated in the clinical trial protocol (e.g., operations required by the protocol)
- Elective hospitalization not related to the worsening of AEs (e.g., elective cosmetic surgery)
- Scheduled treatments or surgeries should be recorded in the entire trial protocol and/or the subject's baseline information
- Hospitalization solely due to the use of blood products

Diagnostic or therapeutic invasive (e.g., surgical) and non-invasive procedures should not be reported as AEs. However, if the disease condition that leads to such a procedure which meets the definition of an adverse event, should be reported. For example, acute appendicitis occurring during the AE reporting period should be reported as an AE, and the subsequent appendectomy should be recorded as a treatment method for that AE.

Death should not be reported as the term for serious adverse events. The medical term for the SAE that leads to death (if there is a diagnostic name, the diagnostic name should be used) is the reporting term for serious adverse events, while death is the outcome of the adverse event.

8.4.5 Reporting System for Serious Adverse Events

The reporting period for serious adverse events should begin when the subject signs the informed consent form and continue until 30 calendar days after the last use of the study medication (including the 30th day). If a serious adverse event occurs, both the initial report and follow-up reports should be made by completing theNew Drug Clinical Study Serious Adverse Event (SAE) Report Form, signing, and dating it, and then immediately reporting it by fax to the responsible research unit, the leading unit, the research unit's ethics committee, the National Medical Products Administration (NMPA, formerly CFDA), and the food and drug administration of the researcher's local area (province or city).

Serious adverse events occurring after the last administration of the drug within 30 days should generally not be reported unless they are suspected to be related to the study medication.

Serious adverse events should be detailed in terms of symptoms, severity, relevance to the trial medication, occurrence time, processing time, measures taken, follow-up time and method, and outcome. If the investigator believes that a serious adverse event is unrelated to the trial medication but potentially related to the research conditions (e.g., termination of original treatment, or comorbidities during the trial process), this relationship should be detailed in the narrative part of the serious adverse event page of the medical record report form. If the intensity of an ongoing serious adverse event or its relationship with the trial medication changes, the follow-up report of the serious adverse event should be immediately submitted to the sponsor. All serious adverse events should be followed up until resolution or stabilization.

Pre-existing Medical Conditions

Symptoms/signs present in subjects during the trial screening period should be recorded and reported as adverse events (AEs) only if there is an increase in severity, frequency, or nature after entering the trial (excluding the worsening of the disease under

study). Records should reflect changes relative to the previous state, such asincreased frequency of headaches.

Disease Progression

Disease progression is defined as a deterioration in the subject's condition caused by the primary tumor targeted by the investigational medication, with the appearance of new lesions related to the primary tumor or the progression of existing lesions considered as disease progression. Expected disease progression is not reported as an AE. Deaths, life-threatening conditions, hospitalizations or prolongation of hospital stays, permanent or severe disabilities/loss of abilities, congenital abnormalities/birth defects, and other significant medical events caused by expected disease progression symptoms and signs are not accelerated as serious adverse events (SAEs) for reporting.

New Anticancer Treatments

Within 90 days after the last administration, if a subject begins new anticancer treatments, only serious adverse events considered related to the study medication are recorded and reported.

8.5 Rapid Reporting of SAEs and Pregnancy

SAE Reporting:

The reporting period for SAEs is from the signing of the informed consent form to 90 days (inclusive) after the last administration of the medication. In the event of an SAE, whether it is the initial report or a follow-up report, the investigator must immediately complete the sponsor'sSerious Adverse Event Report Form and report to the sponsor: drugsafety@innoventbio.com within 24 hours of becoming aware of the event, and report to the national regulatory authority and ethics committee as required by Chinese regulations.

If a serious adverse event occurring outside the aforementioned period is determined to be related to the study medication, it should also be reported.

Investigators must report the completed SAE report form to the sponsor as soon as possible within 24 hours of becoming aware of the SAE. For deaths and life-threatening serious adverse events, investigators should urgently follow up on missing information and provide a complete SAE report. At the same time, investigators report to the national regulatory authority and ethics committee as required by regulations.

Pregnancy

Given the safety risk of embryotoxicity associated with similar drugs, all subjects of childbearing potential participating in the clinical trial must use effective contraceptive

measures.

Should a female subject become pregnant during the clinical trial period when there is drug exposure, the subject will be withdrawn from the study. The investigator must report the pregnancy to the sponsor within 24 hours of becoming aware and complete theIntas Clinical Trial Pregnancy Report/Follow-up Form.

Should a male subject's partner become pregnant during the clinical trial period when there is drug exposure, the subject will continue in the clinical trial. The investigator must report the pregnancy to the sponsor within 24 hours of becoming aware and complete theIntas Clinical Trial Pregnancy Report/Follow-up Form.

The investigator must continuously monitor subjects who experience pregnancy and follow up on the outcomes of pregnancy, continuing follow-up until 8 weeks after the mother gives birth, and report the results to the sponsor.

If the pregnancy results in stillbirth, spontaneous abortion, fetal malformation (any congenital abnormality/birth defect), or medical abortion, it is considered a Serious Adverse Event (SAE) and must be reported following the SAE process and timelines.

If a subject experiences an SAE during pregnancy, it should be reported according to the SAE reporting procedures.

8.6 Events of Abnormal Liver Function

If AST and/or ALT levels are abnormally elevated in conjunction with a significant increase in total bilirubin levels, and there are no other causes of liver injury, it will be considered drug-induced liver injury. Such cases should always be regarded as significant medical events.

Table 12 Liver Function Injury Requiring Reporting as an SAE

Baseline	Normal (AST/ALT and Total Bilirubin)	Abnormal (AST/ALT and Total Bilirubin
Treatment Period	ALT or AST ≥ 3× ULN with Total Bilirubin ≥ 2× ULN and Alkaline Phosphatase ≤ 2× ULN and no hemolysis	AST or ALT ≥ 8× ULN with an increase in Total Bilirubin ≥ 1× ULN or a value ≥ 3× ULN

Subjects should return to the research center for assessment as soon as possible after learning of the abnormal results (preferably within 48 hours).The assessment should include laboratory tests, a detailed medical history inquiry, and a physical evaluation, and the possibility of liver tumors (primary or secondary) should be considered.

In addition to repeating the testing of AST and ALT, the laboratory tests that should be conducted should also include albumin, creatine kinase, total bilirubin, direct bilirubin,

gamma-glutamyl transferase (γ-GT), prothrombin time/international normalized ratio, and alkaline phosphatase. A detailed medical history collection should include: history of alcohol consumption, acetaminophen, soft drugs, various supplements, traditional Chinese medicine, exposure to chemical agents, family medical history, occupational exposure, sexual history, travel history, contact with individuals with jaundice, surgeries, blood transfusions, history of liver disease or allergic diseases, heart disease, and immune disorders should be obtained. Further examinations may also include testing for acute hepatitis A, B, C, and E, liver imaging studies (such as biliary tract), autoantibody tests, and echocardiograms. If repeated tests confirm the laboratory criteria defined in Table 10, and there are no other causes for the abnormal liver function tests, the possibility of potential drug-induced liver injury should be considered without waiting for all the results of etiological tests for liver function abnormalities. Such potential cases of drug-induced liver injury should be reported as SAEs and aLiver Function Abnormality Monitoring and Follow-up Report Form should be submitted to the sponsor.

8.7 Management of Drug-Related Toxicity

The sponsor conducts regular safety audits at the trial level during the conduct of the trial. Detailed information including the frequency of audits and the types of data reviewed will be recorded in a separate trial-level safety audit plan.

8.7.1 Immune-Related Adverse Events

Given the mechanism of action of Sintilimab, which induces T-cell activation and proliferation, immune-related adverse events (irAEs) may be observed during this study. Signs and symptoms of irAEs in subjects should be monitored. If there is no clear alternative etiology (e.g., infection), signs or symptoms of diseases occurring during the trial in subjects should be considered related to the immune system.

Dose adjustments for Sintilimab and principles for adverse event management are detailed in Sections 5.2 and 5.3 of the protocol.

9 Statistical Analysis

9.1 Sample Size Calculation

Based on previous study data, the 1-year PFS rate for patients with extensive-stage small cell lung cancer (SCLC) receiving first-line chemotherapy does not exceed 5.4%. We estimate that the use of first-line Sintilimab combined with chemotherapy can increase the 1-year PFS rate of patients to 15%. Through sample size calculation, with α=0.05 (one-sided test), Power=80%, and assuming a dropout rate of 10%, using the One-Sample Logrank Test, a total of 45 patients need to be enrolled.

9.2 Statistical Analysis Plan

The statistical analysis endpoints for this study include:

Primary endpoint:

- To evaluate the 1-year PFS rate of subjects receiving first-line treatment with a PD-1 inhibitor combined with chemotherapy.

Secondary Endpoints:

- To evaluate the progression-free survival (PFS), overall survival (OS), and objective response rate (ORR) of subjects according to RECIST 1.1;
- To assess the quality of life of subjects based on the EORTC QLQ-C30 scoring;
- To evaluate the incidence of adverse events (AEs) in subjects.

Exploratory Endpoints:

- To identify potential predictive biomarkers of efficacy for immunotherapy in late-stage (extensive-stage) small cell lung cancer.

Statistical analysis will primarily utilize SPSS statistical software for programming and computation. Statistical tests will employ one-tailed and two-tailed tests; a P-value less than or equal to 0.05 will be considered to indicate a statistically significant difference, and confidence intervals will be reported at a 95% confidence level.

Patient Baseline Characteristics:

Calculate the mean, standard deviation, median, maximum, and minimum values for quantitative data such as age, height, and weight; list the frequency and percentage for qualitative data such as gender, ECOG score, etc.

Efficacy Analysis:

PFS and OS will be estimated using the Kaplan-Meier method to determine median values and their 95% CIs, and survival curves will be plotted. The objective response rate (ORR, which equals complete response [CR] plus partial response [PR]) and its 95% CI will be calculated. Quality of life scores will be compared with baseline values using paired t-tests or Wilcoxon signed-rank tests for within-group comparisons before and after treatment.

Safety Analysis:

Descriptive statistical analysis will primarily be used, with tables describing adverse events and adverse reactions (defined asadverse events 'definitely related/likely related/possibly

related' to the study drug) that occurred during the trial. Laboratory test results will describe conditions that were normal before the trial but abnormal after treatment, as well as the relationship between these changes and the study drug. Calculate the mean, standard deviation, median, minimum, and maximum values for vital signs and laboratory indicators before and after medication, using paired t-tests for before-and-after comparisons.

Compliance Analysis:
Summarize by group the proportion and frequency of subjects who violated the expected dosing regimen, the proportion of subjects whose study drug dosage was within 80% to 120% of the protocol-specified dose, and the proportion of subjects who completed the study and different treatment cycles.

10 Quality Assurance and Quality Control

Research personnel must be physicians who have received clinical trial training and work under the guidance of senior professionals.

Pre-trial inspections of clinical wards must meet standardized requirements to ensure that resuscitation equipment is complete.

It is recommended that professional nursing staff administer medication to subjects, understand in detail the medication intake, and ensure subject compliance.

Research centers must strictly follow the study protocol and accurately record case report forms.

Monitors should follow standard operating procedures, supervise the conduct of clinical trials, confirm that all data recording and reporting are correct and complete, all case report forms are entered correctly, and are consistent with the original data, ensuring that the trial is conducted in accordance with the clinical study protocol.

In the event of a Serious Adverse Event (SAE), it must be promptly reported to all research units, and if necessary, the study should be temporarily suspended.

11 Ethical, Regulatory, and Administrative Principles

11.1 Ethical Principles

This study will be conducted in accordance with the principles established by the 18th World Medical Association Joint Conference (Helsinki, 1964) and all subsequent revisions.

11.2 Legal and Regulatory Compliance

This study will be conducted in compliance with all applicable laws and regulations.

11.3 Informed Consent

Before any study procedures begin, the potential subject will be informed of the risks and benefits of this study using an informed consent form. The language of the informed consent should be simple and understandable. The informed consent form should clearly state that it is voluntarily signed and specify the risks and benefits associated with participation in this study.

The investigator is responsible for explaining the content of the informed consent to the subject and obtaining a signed and dated informed consent form from the subject or his/her legal representative before the study begins.

11.4 Data Protection

Personal data of patients and investigators in the database of the responsible research unit (Shanghai Pulmonary Hospital) should be processed in accordance with all applicable local laws and regulations.

When archiving or processing personal data related to investigators and/or patients, the responsible research unit (Shanghai Pulmonary Hospital) should take all appropriate measures to protect and prevent unauthorized third parties from obtaining such information.

11.5 Confidentiality Agreement

All materials, information (oral or written), and unpublished documents provided to the investigators (or any actions taken by the responsible research unit on behalf of the investigators), including this protocol and CRF, are the property of Tongji University Affiliated Shanghai Pulmonary Hospital.

Without prior formal written consent from the principal investigator of the responsible research unit, investigators or any member of their team shall not disclose such materials or information to unauthorized individuals.

Investigators should keep confidential any information received, obtained, or derived during the course of this study, except for information permitted to be disclosed by regulations.All information must be kept confidential, and all necessary steps should be taken to ensure that no disclosure occurs.

11.6 Record Keeping

Investigators should arrange for the storage of study documents until the study is completed. In addition, regarding the storage of patient records, investigators should comply with specific local regulations/guidelines.

Unless otherwise stated in the investigator's agreement, it is recommended that investigators keep study documents for at least five years after the completion or termination of the study, in accordance with other standards and/or local laws.

11.7 Early Termination of the Study

The principal investigator of the responsible research organization may decide to terminate this study at any time and for any reason; the decision to terminate the study will be communicated to the participating investigators in writing.

If applicable, the ethics committee (IRB) and health regulatory authorities should be notified in accordance with local regulations.

11.8 Inspections by the Responsible Research Organization and Regulatory Authorities

Investigators agree to allow inspectors from the responsible research organization/inspectors from regulatory authorities direct access to the study records of subjects for review, and understand that these individuals are bound by professional confidentiality principles, and therefore will not disclose any personal identity or personal medical information of patients.

Investigators will make every effort to assist with inspections and visits, enabling inspectors/visitors to have access to all necessary equipment, data, and documents.

During these inspections, the confidentiality of the data and the protection of the subjects should be respected.

Investigators should immediately communicate the results and information provided after regulatory authority inspections with the responsible research organization.

Investigators should take appropriate corrective measures for any issues identified during audits or inspections, as required by the responsible research organization.

12 Study Management

12.1 Access to Original Data/Documents

The data mentioned may not be used without permission from the principal investigator of the responsible research organization.

The principal investigator of the responsible research organization has full access to the final data to enable appropriate academic analysis and reporting of the study results.

12.2 Protocol Amendments

Any amendments to the protocol will be documented in written amendments, signed by the investigators and the responsible research organization. Signed amendments will be attached to this protocol.

Amendments to this protocol may need to be submitted in accordance with local regulations.

12.3 Publication

All participating investigators hereby grant the principal investigator of the responsible research organization full authority to make the first public announcement and/or the initial publication of the study results. No other publications are allowed before the initial publication. Any subsequent announcements or publications by research participants (including sub-investigators) must be approved by the principal investigator of the responsible research organization and must reference this study and the initial publication. The principal investigator of the responsible research organization has the final decision on any manuscripts, abstracts, or presentations. All manuscripts, abstracts, and presentations must be submitted for internal review by the responsible research organization at least forty-five (45) calendar days prior to submission.

13 Reference

1. Chen W, Zheng R, Baade PD, et al.Cancer statistics in China, 2015. CA: a cancer journal for clinicians 2016, 66(2): 115-132.

2. Siegel RL, Miller KD, Jemal A.Cancer statistics, 2017. CA: a cancer journal for clinicians 2017, 67(1): 7-30.

3. Pesch B, Kendzia B, Gustavsson P, et al.Cigarette smoking and lung cancer—relative risk estimates for the major histological types from a pooled analysis of case-control studies. International journal of cancer 2012, 131: 1210-1219.

4. Antonia SJ, López-Martin JA, Bendell J, et al.Nivolumab alone and nivolumab plus ipilimumab in recurrent small-cell lung cancer (CheckMate 032): a multicentre, open-label, phase 1/2 trial. Lancet Oncology 2016, 17: 883–895.

5. Ready N, Farago AF, de Braud F, et al.Third-Line Nivolumab Monotherapy in Recurrent SCLC: CheckMate 032. Journal of Thoracic Oncology 2019, 14(2): 237-244.

6. Chung HC, Lopez-Martin JA, Kao SCH, et al.Phase 2 study of pembrolizumab in advanced small-cell lung cancer (SCLC): KEYNOTE-158. Journal of Clinical Oncology: official journal of the American Society of Clinical Oncology 2018, 36: Abstract 8506.

7. Alley EW, Lopez J, Santoro A, et al.Clinical safety and activity of pembrolizumab in patients with malignant pleural mesothelioma (KEYNOTE-028): preliminary results from a non-randomised, open-label, phase 1b trial. Lancet Oncology 2017, 18: 623–630.

8. Chung HC, Piha-Paul SA, Lopez-Martin J, et al.Pembrolizumab After Two or More Lines of Previous Therapy in Patients With Recurrent or Metastatic Small-Cell Lung Cancer: Results From the KEYNOTE-028 and KEYNOTE-158 Studies. Journal of Thoracic Oncology: official publication of the International Association for the Study of Lung Cancer 2019.

9. Horn L, Mansfield AS, Szczęsna A, et al.First-line atezolizumab plus chemotherapy in extensive-stage small-cell lung cancer. The New England Journal of Medicine 2018, 379: 2220-2229.
10. Sul J, Blumenthal GM, Jiang X, et al.FDA Approval Summary: Pembrolizumab for the Treatment of Patients With Metastatic Non-Small Cell Lung Cancer Whose Tumors Express Programmed Death-Ligand 1. The Oncologist 2016, 21(5): 643-650.
11. Gadgeel SM, Pennell NA, Fidler MJ, et al.Phase II Study of Maintenance Pembrolizumab in Patients with Extensive-Stage Small Cell Lung Cancer (SCLC). Journal of Thoracic Oncology: official publication of the International Association for the Study of Lung Cancer 2018.
12. Hellmann MD, Callahan MK, Awad MM, et al.Tumor Mutational Burden and Efficacy of Nivolumab Monotherapy and in Combination with Ipilimumab in Small-Cell Lung Cancer. Cancer Cell 2018, 33(5): 853-861 e854.
13. Hellmann MD, Callahan MK, Awad MM, et al.Tumor Mutational Burden and Efficacy of Nivolumab Monotherapy and in Combination with Ipilimumab in Small-Cell Lung Cancer [published correction appears in Cancer Cell. 2019 Feb 11;35(2):329]. Cancer Cell 2018; 33(5): 853–861.e4.
14. Ayers M, Lunceford J, Nebozhyn M, et al.IFN-γ-related mRNA profile predicts clinical response to PD-1 blockade. J Clin Invest. 2017; 127(8): 2930–2940.
15. Ott PA, Bang YJ, Piha-Paul SA, et al.T-Cell-Inflamed Gene-Expression Profile, Programmed Death Ligand 1 Expression, and Tumor Mutational Burden Predict Efficacy in Patients Treated With Pembrolizumab Across 20 Cancers: KEYNOTE-028. J Clin Oncol. 2019; 37(4): 318–327.
16. Hardy-Werbin M, Rocha P, Arpi O, et al.Serum cytokine levels as predictive biomarkers of benefit from ipilimumab in small cell lung cancer. Oncoimmunology. 2019; 8(6): e1593810. Published 2019 Mar 27.
17. Hardy-Werbin M, Arpí O, Taus A, et al.Assessment of neuronal autoantibodies in patients with small cell lung cancer treated with chemotherapy with or without ipilimumab. Oncoimmunology. 2017; 7(2): e1395125. Published 2017 Nov 27.
18. McGranahan N, Furness AJ, Rosenthal R, et al.Clonal neoantigens elicit T cell immunoreactivity and sensitivity to immune checkpoint blockade. Science. 2016; 351(6280): 1463–1469.
19. Gettinger SN, Choi J, Mani N, et al.A dormant TIL phenotype defines non-small cell lung carcinomas sensitive to immune checkpoint blockers. Nat Commun. 2018; 9(1): 3196. Published 2018 Aug 10.

20. Han J, Duan J, Bai H, et al.TCR Repertoire Diversity of Peripheral PD-1+CD8+ T Cells Predicts Clinical Outcomes after Immunotherapy in Patients with Non-Small Cell Lung Cancer. Cancer Immunol Res. 2020; 8(1): 146–154.

14 Appendix

Appendix 1: Performance Status Scoring Criteria (ECOG PS)

**Activity Score**	**Description**
0	Asymptomatic, fully active, and able to perform unrestricted activities.
1	Symptomatic, fully ambulatory, but limited to heavy physical activity, capable of light or sedentary work, such as light household work, office work.
2	Symptomatic, capable of walking, self-care, but unable to perform any physical activity, more than 50% of waking time is spent awake (daytime bed rest time < 50%).
3	Symptomatic, limited self-care capability, more than 50% of waking hours in bed or seated, but not yet bedbound.
4	Completely disabled, unable to care for self, bedbound.
5	Death.

Appendix 2: Formulas for Calculating Creatinine Clearance and Body Surface Area

Creatinine Clearance is calculated using the Cockcroft-Gault formula.
For serum creatinine concentration in units of (mg/dL), the calculation formula is:

Male Creatinine Clearance (mL/min)
$=(140-\text{Age})\times(\text{Weight})^{a}/(72\times\text{Serum Creatinine})$

Female Creatinine Clearance (mL/min) = $0.85\times(140-\text{Age})\times(\text{Weight})^{a}$
$/(72\times\text{Serum Creatinine})0.85\times(140-\text{Age})\times(\text{Weight})a/(72\times\text{Serum Creatinine})$

For serum creatinine concentration in units of (μmol/L), the calculation formula is:

Male Creatinine Clearance (mL/min) = $(140-\text{Age})\times(\text{Weight})^{a}$
$/(0.81\times\text{Serum Creatinine})(140-\text{Age})\times(\text{Weight})a/(0.81\times\text{Serum Creatinine})$

Female Creatinine Clearance (mL/min) = 0.85×(140−Age)×(Weight) [a] /(0.81×Serum Creatinine)0.85×(140−Age)×(Weight)*a*/(0.81×Serum Creatinine)

a: Age is in years, and weight is in kg.

Body Surface Area Calculation Formula
Body Surface Area (m^2) = 0.00616 × Height (cm) + 0.01286 × Weight (kg) - 0.1529

Appendix 3: Response Evaluation Criteria in Solid Tumors (RECIST) Version 1.1 (Excerpt)

1. Measurability of Tumors at Baseline
1.1 Definition
At the baseline, tumor lesions/lymph nodes will be categorized into measurable and non-measurable according to the following definitions:
1.1.1 Measurable Lesions
Tumor lesions: Must have at least one dimension that can be accurately measured (recorded as the longest diameter), with the minimum length as follows:
- CT scan 10 mm (CT scan slice thickness no greater than 5 mm)
- Clinical routine examination instruments 10 mm (if the tumor lesion cannot be accurately measured with a measuring instrument, it should be recorded as non-measurable)
- Chest X-ray 20 mm
Malignant lymph nodes: Pathologically enlarged and measurable, the short diameter of a single lymph node on CT scan must be ≥15 mm (it is recommended that the CT scan slice thickness does not exceed 5 mm). At baseline and follow-up, only the short diameter is measured and followed up.

1.1.2 Non-measurable Lesions
All other lesions, including small lesions (longest diameter <10 mm or pathological lymph node short diameter ≥10 mm to <15 mm) and lesions that cannot be measured. Non-measurable lesions include: meningeal disease, ascites, pleural or pericardial effusion, inflammatory breast cancer, cutaneous/pulmonary cancer lymphangitis, abdominal masses that cannot be

confirmed and followed up by imaging, and cystic lesions.

1.1.3 Special Considerations for Lesion Measurement

Bone lesions, cystic lesions, and lesions that have previously received local treatment need special notation:

Bone lesions:

1) Bone scans, PET scans, or plain films are not suitable for measuring bone lesions but can be used to confirm the presence or disappearance of bone lesions;

2) Lytic or mixed lytic/blastic bone lesions with a definite soft tissue component, and the soft tissue meet the aforementioned definitions of measurability, and if these lesions can be assessed using sectional imaging techniques such as CT or MRI, then these lesions can be considered measurable lesions.

3) Osteoblastic lesions are considered non-measurable lesions.

Cystic lesions:

1) Lesions that meet the radiographic definition of a simple cyst should not be considered malignant lesions simply because they are defined as simple cysts; they are neither measurable nor non-measurable lesions.

2) If they are cystic metastatic lesions and meet the above definition of measurability, they can be considered measurable lesions. However, if there are non-cystic lesions in the same patient, non-cystic lesions should be prioritized as target lesions.

Locally treated lesions:

Lesions located in areas that have undergone radiotherapy or other local regional treatments are generally considered non-measurable lesions unless there is clear progression of the lesion. The study protocol should describe in detail the conditions under which these lesions are considered measurable lesions.

1.2 Measurement Method Description

1.2.1 Lesion Measurement

In clinical evaluation, all tumor measurements should be recorded in the metric system. All baseline assessments of tumor lesion size should be completed as

close to the start of treatment as possible. This study requires that they must be completed within 28 days before the start of treatment.

1.2.2 Evaluation Methods

The same techniques and methods should be used for the baseline assessment of lesions and subsequent measurements. Except for lesions that cannot be evaluated by imaging and can only be evaluated by clinical examination, all lesions must be evaluated using imaging.

Clinical lesions: Clinical lesions are only considered measurable if they are superficial and have a diameter of ≥10 mm when measured (such as skin nodules). For patients with skin lesions, it is recommended to use color photographs containing a ruler to measure the size of the lesions for archiving. When lesions are evaluated using both imaging and clinical examination, imaging should be preferred whenever possible due to its objectivity and the ability to review it repeatedly at the end of the study.

Chest X-ray: When tumor progression is an important study endpoint, chest CT should be prioritized because CT is more sensitive than X-ray, especially for new lesions. Chest X-ray detection is only applicable when the measured lesion boundaries are clear and lung ventilation is good.

CT, MRI: CT is currently the best available reproducible method for efficacy evaluation. The definition of measurability in this guideline is based on a CT scan slice thickness of ≤5 mm. If the CT slice thickness is greater than 5 mm, the smallest measurable lesion should be twice the slice thickness. MRI may also be acceptable in some cases (such as whole-body scanning).

Ultrasound: Ultrasound should not be used as a measurement method for lesion size. Due to the operator-dependence of ultrasound examinations, there is no repeatability after measurement, and the consistency of technique and measurement between different measurements cannot be guaranteed. If new lesions are detected using ultrasound during the trial period, they should be confirmed using CT or MRI. If consideration is given to the radiation exposure from CT, MRI can be used as a substitute.

Endoscopic and Laparoscopic Examinations: These techniques are not recommended for the objective evaluation of tumors, but they can be used to confirm a Complete Response (CR) when biopsy specimens are obtained. They may also be used to confirm recurrence in trials where the study endpoint is recurrence after CR or surgical resection.

2. Assessment of Tumor Response

2.1 Target Lesion Assessment

- Complete Response (CR): All target lesions disappear, and the short-axis diameter of all pathological lymph nodes (including target and non-target nodules) must be reduced to less than 10 mm.
- Partial Response (PR): The sum of the diameters of target lesions decreases by at least 30% compared to the baseline level.
- Progressive Disease (PD): Compared to the smallest sum of the diameters of all measured target lesions during the entire study, there is an increase of at least 20% relative to this value (if the smallest value at baseline, then use the baseline value as a reference); in addition, there must be an absolute increase of at least 5 mm in the sum of diameters (the appearance of one or more new lesions is also considered as disease progression).
- Stable Disease (SD): The degree of reduction in target lesions does not reach PR, and the degree of increase does not reach PD levels, falling between the two, and the smallest sum of diameters during the study can be used as a reference.

2.2 Notes on Target Lesion Assessment

- Lymph Nodes: Even if the short-axis diameter of lymph nodes identified as target lesions is reduced to less than 10 mm, the actual short-axis diameter corresponding to the baseline should still be recorded during each measurement (consistent with the anatomical plane at baseline measurement). This means that if the lymph node is a target lesion, even if it meets the criteria for complete response, it cannot be said that the lesion has completely disappeared because the short-axis diameter of a normal lymph node is defined as less than 10 mm. In the CRF form or other recording methods, target lymph

node lesions should be specifically recorded at a designated location: for CR, all lymph node short diameters must be less than 10 mm; for PR, SD, and PD, the actual measured values of the target lymph node short diameters will be included in the sum of the target lesion diameters.

- Small to Non-measurable Target Lesions: In clinical studies, all lesions (nodular or non-nodular) recorded at baseline should be recorded again with the actual measurement values in subsequent assessments, even if the lesions are very small (e.g., 2 mm). However, sometimes they may be too small to produce a clear image on CT scans, making it difficult for radiologists to define an exact value and possibly reporting them as "too small to measure." In such cases, it is crucial to record the last known value on the eCRF form. If the radiologist believes the lesion may have disappeared, it should be recorded as 0 mm.

If a lesion is indeed present but is vague and cannot provide an exact measurement, it may be defaulted to 5 mm. (Note: This situation is unlikely for lymph nodes, as they generally have measurable dimensions under normal conditions, or are often surrounded by fat tissue, such as in the retroperitoneal space; however, if such a situation arises where a measurement cannot be given, it is also defaulted to 5 mm). The default value of 5 mm originates from the slice thickness of CT scans (this value does not change with different slice thicknesses of CT). Since the likelihood of the same measurement being repeated is low, providing this default value will reduce the risk of incorrect assessment. However, it must be reiterated that if the radiologist can provide an exact value for the size of the lesion, even if the lesion diameter is less than 5 mm, the actual value must be recorded.

Separated or combined lesions: When non-nodular lesions split into fragmented shapes, the longest diameter of each separated part is added together to calculate the sum of the lesion diameters. Similarly, for combined lesions, they can be distinguished by planes between each combined part, and then their respective maximum diameters are calculated. However, if they are inseparable, the longest diameter should be taken as the longest diameter of the entire fused lesion.

2.3 Assessment of Non-target Lesions

This section defines the response criteria for non-target lesion tumors. Although some non-target lesions are actually measurable, they do not need to be measured; only a qualitative assessment is required at the time points specified in the protocol.

- Complete Response (CR): All non-target lesions disappear, and tumor markers return to normal levels. All lymph nodes are non-pathological in size (short-axis diameter <10 mm).
- Partial Response/Non-progressive Disease: The presence of one or more non-target lesions and/or persistent levels of tumor markers above normal levels.
- Progressive Disease: A clear progression of existing non-target lesions. Note: The appearance of one or more new lesions is also considered progressive disease.

2.4 Special Notes on the Assessment of Non-target Lesion Progression

Supplementary explanations regarding the definition of progression of non-target lesions are as follows: When a patient has measurable non-target lesions, even if the target lesion assessment is stable or partially responsive, to define clear progression based on non-target lesions, the overall deterioration of non-target lesions must reach a level that necessitates termination of treatment. A general increase in the size of one or more non-target lesions is often not sufficient to meet the criteria for progression; therefore, defining overall tumor progression based solely on changes in non-target lesions when target lesions are stable or partially responsive is almost rare.

When a patient's non-target lesions are all non-measurable: This situation occurs in some Phase III trials where the inclusion criteria do not specify the presence of measurable lesions. The overall assessment still refers to the standards mentioned above, but since there are no measurable data for lesions in this case. The deterioration of non-target lesions is not easy to assess (by definition: all non-target lesions must indeed be unmeasurable), so when changes in non-target lesions lead to an overall increase in disease burden equivalent to that of disease progression in target lesions, defining clear progression based on non-target lesions requires the establishment of an

effective detection method for assessment. For example, an increase in tumor burden equivalent to a 73% increase in volume (equivalent to a 20% increase in the diameter of measurable lesions). Other examples include the progression of peritoneal exudate from "minimal" to "large amount"; lymphatic disease from "localized" to "widespread dissemination"; or described in the protocol as "sufficient to change the treatment method." Examples include the progression of pleural effusion from trace to large amounts, lymphatic involvement spreading from the primary site to distant sites, or possibly described in the protocol as "necessitating a change in the treatment approach." If clear progression is identified, the patient should be considered to have overall disease progression at that point. It is best to have objective criteria applicable to the assessment of non-measurable lesions, noting that the criteria for increase must be reliable.

2.5 New Lesions

The appearance of new malignant lesions indicates disease progression; therefore, evaluation of new lesions is very important. Currently, there are no specific standards for imaging detection of lesions, yet the discovery of a new lesion should be clear. For instance, progression should not be attributed to differences in imaging technology, changes in imaging morphology, or other lesions outside of the tumor (e.g., some so-called new bone lesions are merely healing or recurrence of the original lesion).

This is particularly important when a patient's baseline lesions show partial or complete response. For example, necrosis of a liver lesion might be reported as a new cystic lesion on a CT scan, which it is not.

Lesions detected during follow-up that were not found during the baseline examination are considered new lesions and indicate disease progression. For example, a patient found to have visceral lesions at baseline, when undergoing a head CT or MRI examination, discovers metastatic foci; such intracranial metastatic lesions are considered evidence of disease progression, even if the patient did not have a head examination at baseline.

If a new lesion is ambiguous, such as due to its small size, further treatment

and follow-up evaluation are needed to confirm whether it is a new lesion. If repeated examinations confirm that it is a new lesion, then the time of disease progression should be counted from the time of its initial discovery.

Lesion assessment by FDG-PET generally requires additional testing for supplementary confirmation. It is reasonable to evaluate the progression in combination with FDG-PET and supplementary CT examination results (especially for new suspicious diseases). New lesions can be clearly identified by FDG-PET assessment, carried out according to the following procedure:

- If the baseline FDG-PET examination result is negative, and the subsequent follow-up FDG-PET examination is positive, it indicates disease progression.
- If no baseline FDG-PET examination was performed, and the subsequent FDG-PET examination result is positive:
- If the follow-up FDG-PET positive examination identifies new lesions that are consistent with the CT examination results, it proves disease progression.

If the positive findings from a follow-up FDG-PET scan cannot be confirmed by CT scan results, a further CT scan is required for confirmation (if confirmed, the progression date is counted from the abnormal findings of the previous FDG-PET scan).

If the positive findings from a follow-up FDG-PET scan correspond to lesions already identified by CT scan, and there is no progression of these lesions on imaging, then there is no disease progression.

2.6 Explanation of Assessment Missing and Non-evaluable

If imaging or measurement of a lesion cannot be performed at a specific time point, the patient is considered unevaluable at that time point. If only a portion of the lesions can be assessed during an evaluation, this situation is generally considered unevaluable at that time point, unless there is evidence to confirm that the missing lesions will not affect the efficacy response assessment at the designated time point.

2.7 Special Notes on Efficacy Assessment

When nodular lesions are included in the overall target lesion assessment and the size of these nodules decreases to a "normal" size (<10mm), they will still have a lesion size scan report. To avoid overestimating the situation reflected by the increase in nodule size, even if the nodule is normal, the measurement results will be recorded. As previously mentioned, this means that subjects with a complete response will not be recorded as 0 on the eCRF form.

If efficacy confirmation is required during the trial process, repeated "unevaluable" time points will complicate the best efficacy assessment. The trial's analysis plan must clarify how these missing data/assessments can be interpreted when determining efficacy. For example, in most trials, a subject's response of PR-NE-PR can be considered as having received efficacy confirmation.

When a subject experiences an overall deterioration in health that requires cessation of drug treatment, but without objective evidence, it should be reported as symptomatic progression. Even after treatment is discontinued, efforts should be made to assess the objective progression.

Symptomatic deterioration is not an assessment description of objective response; it is the reason for stopping treatment. The objective response of such subjects will be assessed through the target and non-target lesion conditions shown in Appendices 1 to 3.

Early progression, early death, and unevaluable situations are special cases of the study and should be clearly described in each protocol (depending on the treatment interval and treatment cycle).

In some cases, it is difficult to distinguish local lesions from normal tissue. When the assessment of complete response is based on such a definition, we recommend performing a biopsy before the efficacy assessment of complete response of local lesions. When some subjects' abnormal imaging results of local lesions are considered to represent lesion fibrosis or scar formation, FDG-PET is used as an assessment standard similar to biopsy for confirming complete response. In such cases, the application of FDG-PET should be

described prospectively in the protocol, and specifically for this situation.

The reporting of medical literature is used as support. However, it must be recognized that the limitations of FDG-PET and biopsy themselves (including the resolution and sensitivity of both) will lead to false-positive results in the assessment of complete response.

Appendix 1: Response at Time Point - Subjects with Target Lesions (Including or Excluding Non-target Lesions)

Target Lesions	Non-target Lesions	New Lesions	Overall Response
CR	CR	None	CR
CR	Non-CR/Non-PD	None	PR
CR	Not evaluable	None	PR
PR	Non-progression or not fully evaluable	None	PR
SD	Non-progression or not fully evaluable	None	SD
Not evaluable	Non-progression	None	NE
PD	Any condition	Any condition	PD

Note: CR = Complete Response, PR = Partial Response, SD = Stable Disease, PD = Progressive Disease, NE = Not Evaluable.

Appendix 2: Response at Time Point - Subjects with Only Non-target Lesions

Non-target Lesions	New Lesions	Overall Response
CR	None	CR
Non-CR or Non-PD	None	Non-CR or Non-PD
Not evaluable	None	Not evaluable
Indeterminate PD	Yes or No	PD

Note: For non-target lesions, "Non-CR/Non-PD" refers to an effect better than SD. Since SD is increasingly used as an endpoint for evaluating efficacy, the efficacy of Non-CR/Non-PD is established to target cases where no lesions are measurable.

For unclear findings of progression (such as very small uncertain new lesions;

cystic changes or necrotic changes in existing lesions), treatment can continue until the next assessment. If progression is confirmed at the next assessment, the progression date should be the date when suspected progression first appeared.

Appendix 3: Best Overall Response Requiring Confirmation for CR and PR Efficacy

First Time Point Overall Response	Subsequent Time Point Overall Response	Best Overall Response
CR	CR	CR
CR	PR or SD or PD	SD if SD lasts long enough, otherwise PD
CR	PD	If SD lasts for a sufficient duration, then SD; otherwise, PD
CR	NE	If SD lasts for a sufficient duration, then SD; otherwise, NE
PR	CR	PR
PR	PR	PR
PR	SD	SD
PR	PD	If SD lasts for a sufficient duration, then SD; otherwise, PD
PR	NE	If SD lasts for a sufficient duration, then SD; otherwise, NE
NE	NE	NE

Note: CR stands for Complete Response, PR for Partial Response, SD for Stable Disease, PD for Progressive Disease, and NE for Not Evaluable. Superscript "a": If a true CR occurs at the first time point, and any disease progression occurs at subsequent time points, then even if the subject's response meets the PR criteria relative to baseline, the response evaluation at later time points is still PD (because the disease will reappear after CR). The best response depends on whether SD occurs within the shortest treatment

interval. However, sometimes the first evaluation is CR, but subsequent scans suggest that small lesions still appear, thus the subject's response at the first time point should actually be PR rather than CR. In this case, the initial CR judgment should be changed to PR, and the best response is PR.

2.8 Efficacy Assessment/Confirmation of Response Duration

2.8.1 Efficacy Confirmation

For non-randomized clinical studies with tumor response as the primary endpoint, it is necessary to confirm the efficacy of PR and CR to ensure that the efficacy is not the result of an evaluation error. In studies with stable disease or disease progression as the primary endpoints, efficacy confirmation is no longer required, as it does not have value for the interpretation of the trial results. In the case of SD, at least one measurement that meets the SD criteria specified in the protocol should be made within the shortest time interval after the start of the trial (generally not less than 6 to 8 weeks).

2.8.2 Overall Response Duration

The overall response duration is the time from the first measurement that meets the CR or PR criteria (whichever is measured first) to the first true record of disease recurrence or progression (using the smallest measurement value recorded in the trial as the reference for disease progression). The overall complete response duration is the time from the first measurement that meets the CR criteria to the first true record of disease recurrence or progression.

2.8.3 Stable Disease Duration

The duration from the start of treatment to disease progression, using the smallest sum in the trial as the reference (if the baseline sum is the smallest, it serves as the reference for calculating PD). The clinical relevance of the stable disease duration varies depending on different studies and different diseases. If the study endpoint is to maintain the proportion of patients with the shortest duration of stable disease, the protocol should specify the minimum time interval between the two measurements defined for SD (Stable Disease).

Note: The duration of response, stable period, and PFS (Progression-Free

Survival) are influenced by the frequency of follow-up after baseline evaluation. The definition of standard follow-up frequency is not within the scope of this guideline. The frequency of follow-up should take into account many factors, such as the type and stage of the disease, the treatment cycle, and standard protocols. However, if comparisons between trials are to be made, the limitations of the accuracy of these measurement endpoints should be considered.

Appendix 4: Quality of Life Scoring

EORTC QLQ-C30 (Version 3.0 Chinese)

We are very interested in learning about you and your physical condition. Please answer the following questions personally and circle the answer that best suits you. There are no "right" or "wrong" answers; simply circle the one that applies to you.

The information you provide will be kept strictly confidential.

Patient's Signature:

Date of Birth: /

Date of Completion: /

1： *Not at all* 2： *A little* 3： *Quite a bit* 4： *Very much*

1. Do you have difficulty doing some strenuous activities, such as lifting heavy shopping bags or suitcases? 1 2 3 4
2. Do you have difficulty walking long distances? 1 2 3 4
3. Do you have difficulty walking short distances outside? 1 2 3 4
4. Do you need to lie in bed or stay in a wheelchair during the day? 1 2 3 4
5. Do you need help with eating, dressing, washing, or using the toilet?

In the past week

6. Have you been limited in doing your work or other daily activities? 1 2 3 4
7. Have you been limited in pursuing your hobbies or other leisure activities? 1 2 3 4

8. Do you experience shortness of breath? 1 2 3 4
9. Have you had pain? 1 2 3 4
10. Do you need to rest? 1 2 3 4
11. Do you have trouble sleeping? 1 2 3 4
12. Do you feel weak? 1 2 3 4
13. Do you lack appetite? 1 2 3 4
14. Do you feel nauseated? 1 2 3 4
15. Have you vomited? 1 2 3 4
16. Have you had constipation? 1 2 3 4
17. Have you had diarrhea? 1 2 3 4
18. Do you feel fatigued? 1 2 3 4
19. Does pain interfere with your daily activities? 1 2 3 4
20. Do you have difficulty concentrating on things, such as reading newspapers or watching TV? 1 2 3 4
21. Do you feel nervous? 1 2 3 4
22. Do you feel anxious? 1 2 3 4
23. Do you get irritable easily? 1 2 3 4
24. Do you feel depressed? 1 2 3 4
25. Have you had any memory difficulties? 1 2 3 4
26. Has your physical condition or medical treatment interfered with your family life? 1 2 3 4
27. Has your physical condition or medical treatment interfered with your social life? 1 2 3 4
28. Has your physical condition or medical treatment caused you financial difficulties? 1 2 3 4

For the following questions, circle the number from 1 to 7 that best matches your answer.

29. How would you rate your overall health status last week?
1 2 3 4 5 6 7
Very poor - Very good

30. How would you rate your overall quality of life last week?
1 2 3 4 5 6 7

Very poor - Very good

1. Quality of Life Scoring Instructions:

The EORTC's QLQ-C30 (Version 3.0) is a core questionnaire for all cancer patients, with a total of 30 items.Items 29 and 30 are divided into seven levels, which are scored from 1 to 7 based on the response options; the other items are divided into 4 levels: Not at all, A little, Quite a bit, and Very much, and are directly scored from 1 to 4 points.

2. Calculation of EORTC QLQ-C30 Domain (Dimension) Scores (Raw Score):

For the convenience of statistical analysis and application, the questionnaire is often divided into certain domains. A domain is an aspect of the quality of life constitution, also known as a dimension, and is treated as an independent variable during analysis.

The 30 items of the EORTC QLQ-C30 (Version 3.0) can be divided into 15 domains, including 5 functional domains (physical, role, cognitive, emotional, and social functioning), 3 symptom domains (fatigue, pain, and nausea/vomiting), 1 overall health status/quality of life domain, and 6 single items (each as a domain). The classification is shown in the table below.

To obtain the score (raw score RS) for a domain, add up the scores of the items included in the domain and divide by the number of items included, i.e., RS = (Q1 + Q2 + ... + Qn) / n.

EORTC QLQ-C30 Domain Classification

Physical Functioning	5	1-5
Role Functioning	2	6-7
Emotional Functioning	4	21 - 24
Cognitive Functioning	2	20 - 25
Social Functioning	2	26 - 27
Global Health Status	2	29 - 30
Fatigue	3	10, 12, 18
Nausea and Vomiting	2	14 - 15
Pain	2	9, 19
Dyspnea	1	8
Insomnia	1	11

Appetite Loss	1	13
Constipation	1	16
Diarrhea	1	17
Financial Difficulties	1	28

3. Calculation of EORTC QLQ-C30 Standardized Scores

To enable comparison of scores across domains, a linear transformation using the range method is further applied to convert raw scores into standardized scores ranging from 0 to 100. Additionally, the transformation serves another purpose, which is to change the direction of the scores. Since the QLQ-C30 scale, except for items 29 and 30, consists of reverse items (the higher the score, the worse the quality of life), the scoring rules explicitly state: for functional domains and the global health status domain, a higher score indicates better functioning and quality of life, while for symptom domains, a higher score indicates more symptoms or problems (worse quality of life). Therefore, when calculating the standardized scores for functional domains, the direction must also be changed. Specifically, the calculations are as follows (where R represents the score range for each domain or item):

Functional Domains: $SS=[1-(RS-1)/R]\times100SS=[1-(RS-1)/R]\times100$

Symptom Domains and Global Health Status Domain:
$SS=[(RS-1)/R]\times100SS=[(RS-1)/R]\times100$
